# Pt/CeO_2_ and Pt/CeSnO_x_ Catalysts for Low-Temperature CO Oxidation Prepared by Plasma-Arc Technique

**DOI:** 10.3389/fchem.2019.00114

**Published:** 2019-03-12

**Authors:** Tatyana Y. Kardash, Elizaveta A. Derevyannikova, Elena M. Slavinskaya, Andrey I. Stadnichenko, Vasiliy A. Maltsev, Alexey V. Zaikovskii, Sergey A. Novopashin, Andrei I. Boronin, Konstantin M. Neyman

**Affiliations:** ^1^Boreskov Institute of Catalysis, Siberian Branch of the Russian Academy of Sciences, Novosibirsk, Russia; ^2^Novosibirsk State University, Novosibirsk, Russia; ^3^Kutateladze Institute of Thermophysics, Siberian Branch of the Russian Academy of Sciences, Novosibirsk, Russia; ^4^Departament de Ciència dels Materials i Química Física, Universitat de Barcelona, Barcelona, Spain; ^5^Institut de Química Teòrica i Computacional, Universitat de Barcelona, Barcelona, Spain; ^6^ICREA (Institució Catalana de Recerca i Estudis Avançats), Barcelona, Spain

**Keywords:** platinum, ceria, tin, CO oxidation, plasma arc synthesis, DFT calculations, Pt/CeO_2_ catalyst

## Abstract

We applied a method of plasma arc synthesis to study effects of modification of the fluorite phase of ceria by tin ions. By sputtering active components (Pt, Ce, Sn) together with carbon from a graphite electrode in a helium ambient we prepared samples of complex highly defective composite PtCeC and PtCeSnC oxide particles stabilized in a matrix of carbon. Subsequent high-temperature annealing of the samples in oxygen removes the carbon matrix and causes the formation of active catalysts Pt/CeO_x_ and Pt/CeSnO_x_ for CO oxidation. In the presence of Sn, X-Ray Diffraction (XRD) and High-Resolution Transmission Electron Microscopy (HRTEM) show formation of a mixed phase CeSnO_x_ and stabilization of more dispersed species with a fluorite-type structure. These factors are essential for the observed high activity and thermic stability of the catalyst modified by Sn. X-Ray Photoelectron Spectroscopy (XPS) reveals the presence of both Pt^2+^ and Pt^4+^ ions in the catalyst Pt/CeO_x_, whereas only the state Pt^2+^ of platinum could be detected in the Sn-modified catalyst Pt/CeSnO_x_. Insertion of Sn ions into the Pt/CeO_x_ lattice destabilizes/reduces Pt^4+^ cations in the Pt/CeSnO_x_ catalyst and induces formation of strikingly high concentration (up to 50% at.) of lattice Ce^3+^ ions. Our DFT calculations corroborate destabilization of Pt^4+^ ions by incorporation of cationic Sn in Pt/CeO_x_. The presented results show that modification of the fluorite lattice of ceria by tin induces substantial amount of mobile reactive oxygen partly due to affecting geometric parameters of ceria by tin ions.

## Introduction

Pollution of biosphere with toxic emissions is becoming increasingly urgent due to the rapid increase in electric and thermal power production, the production growth in metallurgical and chemical industries as well as growing number of vehicles. Among the most effective methods of solving this problem is efficient catalytic oxidation of CO and hydrocarbons (Farrauto and Bartholomew, 1997; Muraki and Zhang, 2000). The increasing restrictions of the standards for the automotive emissions require either improvement of existing catalysts or creation of fundamentally new ones (Trovarelli, 1996; Montini et al., 2016; Trovarelli and Llorca, 2017).

Broadening temperature ranges of catalysts action and maintaining their activity are important challenges therefore. In this regard, the use of conventional three-component formulations based on PGM-CeO_2_-MO_x_ (where PGM are platinum group metals and MO_x_ are modifying metal oxides) is still promising for catalytic oxidation of CO and hydrocarbons. Palladium is the most commonly used PGM. It is characterized as a PGM very strongly interacting with CeO_2_. According to Fornasiero et al. (Cargnello et al., 2012; Monai et al., 2015), PdO nanoparticles decorated with cerium oxide (core-shell structures Pd@CeO_2_) are promising materials for the efficient oxidation of methane and other hydrocarbons. Such Pd@CeO_2_ structures are capable of oxidizing methane at 200–250°C. However, Farrauto (Farrauto, 2012) pointed out that due to the phase transition PdO-Pd at ~800°C the creation of thermally stable (T > 800°C) catalysts on the basis of this Pd form is difficult. Such a conclusion emphasizes the importance of the strong metal-support interaction (SMSI) effect for palladium, platinum and other PGMs for creation of thermally stable catalysts (Figueroba et al., 2016, 2017; Lykhach et al., 2017a).

The realization of the SMSI effect in PGM-CeO_2_ systems depends crucially on the synthesis procedure. Different synthetic approaches are used to obtain PGM-CeO_2_ catalysts with desired improved characteristics (Boronin et al., 2009; Gulyaev et al., 2012, 2014b; Slavinskaya et al., 2015, 2016, 2018; Vasilchenko et al., 2016; Derevyannikova et al., 2017; Kardash et al., 2017; Kibis et al., 2017). Novel physical preparation methods that utilize the action of thermal and electrical fields, such as laser ablation, magnetron sputtering, “solution-combustion” and plasma methods attracted attention of many research groups (Bera et al., 2003; Baidya et al., 2009; Hegde et al., 2009; Gupta and Hegde, 2010; Hinokuma et al., 2014; Slavinskaya et al., 2016, 2018; Vasilchenko et al., 2016; Stadnichenko et al., 2018, 2019). These methods allow obtaining of highly dispersed PGM forms (ions and clusters) on the surface and inside ceria, which ensures the maximal SMSI effect and appearance of new highly active surface species.

Recently we have developed and applied a plasma-arc method (PAS) for synthesis of Pd/CeO_2_ catalysts (Gulyaev et al., 2014b; Kardash et al., 2017). The PAS method is based on the sputtering of a specific anode material in a vacuum chamber filled with an inert gas under action of a plasma arc discharge. The anode material consists of a graphite rod, in the center of which a mixture of dispersed carbon and PGM metal powders, cerium and/or other components is placed. Sputtering of the anode leads to the appearance of atomic components of carbon and atomically dispersed metals in the arc. Diffusion and convection of the components in a buffer gas leads to their heterogeneous condensation. The condensation products are deposited on the cooled screen. The synthesized material consists of nanoparticles embedded in a carbon matrix. Subsequent high-temperature annealing in oxygen converts the synthesized carbon-based composite into an active oxide catalyst for low-temperature CO oxidation (LTO CO).

The aim of this work was to establish, weather the PAS method also allows obtaining joint phase of Pt and CeO_2_, where Pt is in a highly dispersed ionic state similar to that of Pd in the Pd-doped CeO_2_ joint phase Pd_x_Ce_1−x_O_2−δ_ (Primavera et al., 1998; Priolkar et al., 2002; Scanlon et al., 2011; Gulyaev et al., 2014a; Neitzel et al., 2016). Assuming that stable Pt^2+^ and Pt^4+^ (Bruix et al., 2014) species are formed in the PAS synthesis, it was important to determine concentration of these in the prepared samples and their influence on the catalytic activity

In order to increase the thermal stability and redox ability of the catalysts, Sn was used as a modifying element of the catalysts. Sn^4+^ is assumed to substitute Pt^4+^ and Ce^4+^ in the fluorite structure (Matolín et al., 2008; Zeng et al., 2012; Ayastuy et al., 2014). The formation of the joint Ce_1−x_Sn_x_O_y_ phase was expected to promote the generation of defects and oxygen vacancies in the prepared samples of catalysts, thus increasing their oxygen storage capacity (OSC) and the catalytic activity in LTO CO (Tolla et al., 1999; Nguyen et al., 2003; Gupta et al., 2010). Our previous studied of the Pd/CeO_2_-SnO_2−x_ catalysts showed that introduction of Sn into PdCeC formulations during the PAS synthesis formed nano-heterogeneous composites after calcination of the PdCeSnC composite. The obtained catalysts were stable against sintering and crystallization to large particles (Kardash et al., 2017). As a result, LTO CO activity was preserved even after calcination at 1,000°C. One of the objectives of this study was to determine the thermal stability of composite Pt/CeO_2_-SnO_2_ catalysts and to compare their catalytic properties with those of the unmodified by Sn Pt/CeO_2_ catalysts prepared under the same PAS conditions.

Also in this paper we present density functional (DFT) calculations of platinum species stabilized either by pure ceria nanoparticles or ceria nanoparticles modified by tin ions. The calculations of these models allowed establishing the most stable structures and the effect of tin substitution on the properties of Pt/CeO_2_ nanoparticles. Our DFT calculations corroborate the effect of embedded tin on the state of platinum ions in the CeO_2_ lattice.

Obtained experimental and theoretical data showed that the PAS method allows obtaining highly defective ceria particles and ionic platinum species. Modification with tin leads to increased thermal stability of platinum catalysts, higher amount of reactive oxygen in the catalysts and enhanced catalytic activity of the latter in the low-temperature regime of CO oxidation.

## Experimental

### Catalysts Synthesis

PAS method was applied to obtain PtCeC and PtCeSnC composite materials. We refer to the detailed description of the procedure in our previous work (Kardash et al., 2017). To obtain oxide catalysts, the synthesized PtCe(Sn)C composites were calcined in air at 600, 800, and 900°C. Table 1 presents the chemical composition and characteristics of the obtained Pt/Ce and Pt/CeSn catalysts.

**Table 1:** Chemical compositions and BET specific surface area of the prepared samples.

**Sample**	Chemical composition, % wt^*^			**S**_****BET****_**, m**^****2****^**/g**			
	**Pt**	**CeO_**2**_**	**SnO_**2**_**	**600°C**	**700°C**	**800°C**	**900°C**
Pt/Ce	4.6	88	–	110	71	48	10
Pt/CeSn	2.4	12	79	60	39	27	16

* *The rest is carbon and water*.

### Methods

#### The Specific Surface

The specific surface (SBET) of the samples was determined by BET method (Lowell et al., 2006), using argon thermal desorption with a Sorbtometr-M adsorption analyzer.

#### Analysis of the Chemical Composition

Analysis of the chemical composition was performed by X-ray fluorescence (XRF) method on an ARL PERFORM analyzer with a Rh anode of an X-ray tube. Chemical compositions of tin and cerium oxides were determined using calibrations; the determination of Pt was carried out using the external standard method.

#### X-Ray Diffraction (XRD)

X-ray diffraction (XRD) patterns were recorded on a Bruker D8 Advance instrument using CuKα radiation and the Bragg-Brentano focusing geometry. The aperture of the Soller slits on the primary and reflected beams was 2.5°. LynxEye (Bruker) multi strip detector was employed for intensity measurements. Data acquisition was performed in the 2θ range of 15–100°, at a 0.05° step and counting time of 2 s. Phase analysis was carried out using the ICDD PDF 2 database (Powder Diffraction File PDF-2. International Center for Diffraction Data. USA. 2009). Structural data were taken from the structural database ICSD (Hellenbrandt, 2004). Rietveld refinement for quantitative analysis was carried out with the help of the software package Topas V.4.3 (Coelho, 2005). The instrumental broadening was described with metallic silicon as a reference material. The diffraction line profiles were analyzed using the fundamental parameter approach. The lengths of coherent scattering domain were calculated from LVol-IB values (i.e., volume weighted mean column lengths based on integral breadth).

#### Electron Microscopy

Electron microscopy investigation was performed using JEM-2010 (JEOL Ltd., Japan) and JEM-2200FS (JEOL Ltd., Japan) electron microscopes operated at 200 kV for obtaining High Resolution Transmission Electron Microscopy (HRTEM) images. High-Angle Annular Dark-Field mode in a Scanning Transmission Electron Microscope (STEM HAADF) was employed together with Energy Dispersive X-ray (EDX) spectroscopy. Samples for the TEM study were prepared on the perforated carbon film mounted on a copper grid.

#### Temperature Programmed Reaction With CO (TPR-CO)

The reaction mixture containing 1.0 vol.% CO, 0.5 vol.% Ne in flowing He was fed at a rate of 100 cm^3^/min to the catalyst sample (0.2 g) preliminary cooled in the reactor to −10°C. As the steady-state concentrations of CO and CO_2_ were established, the sample was heated from −10 to 450°C at 10°C/min heating rate. The concentrations of CO, CO_2_, O_2_, H_2_, and H_2_O were measured during the reaction. Before each TPR-CO experiment the catalysts were pretreated by 20%O_2_/He gas mixture at 450°C during 2 h with subsequent cooling in this gas mixture. After the cooling the catalysts were purged in helium.

#### Investigation of Catalytic Properties

Investigation of catalytic properties of the synthesized samples was performed in an automated setup with a flow reactor and mass-spectrometric analysis of the gas mixture using the temperature-programmed reaction (TPR-CO-O_2_). A sample with the particle sizes of 0.25–0.5 mm was mounted in a stainless steel reactor. The reaction mixture containing 0.2 vol.% CO, 1.0 vol.% O_2_, 0.5 vol.% Ne in flowing He was fed at a rate of 1,000 cm^3^/min to the initial catalyst cooled to −10°C. For the Pt/Ce catalysts calcined at 600, 700, 800, and 900°C weight of samples employed for catalytic testing was 0.3, 0.36, 0.4, and 0.62 g, respectively; for the Pt/CeSn catalysts calcined at 600, 700, 800, and 900°C −0.3, 0.34, 0.35, and 0.34 g. The catalyst was heated in the reaction mixture from −10 to 450°C at a rate of 10°C/min with subsequent cooling and repeated heating in the reaction mixture. The concentrations of CO, O_2_, and CO_2_ were monitored in the course of reaction at a frequency of 0.34 Hz.

#### X-Ray Photoelectron Spectroscopy (XPS)

X-ray photoelectron spectroscopy (XPS) measurements used an ES-300 (KRATOS Analytical) photoelectron spectrometer equipped with a MgKα (hν = 1253.6 eV) radiation source. The spectrometer was calibrated using the Au4f_7/2_ (84.0 eV) and Cu2p_3/2_ (932.7 eV) lines of pure metallic surfaces of Au and Cu, respectively. The U^*′′′*^ component of the Ce3d spectral line (E_b_ = 916.7 eV) served as a standard for calibration of XPS spectra. The program of processing spectral data created by us, XPS-Calc, was used for the mathematical treatment of the XPS spectra. This software has previously been applied for different systems, including Pt/CeO_2_ and Pt/Al_2_O_3_ catalysts (Ivanova et al., 2010; Svintsitskiy et al., 2015; Stadnichenko et al., 2017, 2019). The Shirley model and the Gauss–Lorentz functions were used for background subtraction and curve fitting, respectively.

#### DFT Calculations

DFT calculations were carried out employing PW91 exchange-correlation functional (Perdew et al., 1992) in the plane-wave VASP code (Kresse and Hafner, 1993) for periodic boundary conditions. Plane-wave basis sets with a cut-off energy of 415 eV were used. Core-valence electron interactions were described by projector-augmented wave method (Kresse and Joubert, 1999). In order to properly localize Ce4f electrons in partially reduced Ce^3+^ ions, an on-site Coulombic correction (U_eff_ = 4.0 eV) (Anisimov et al., 1997; Dudarev et al., 1998; Loschen et al., 2007) was applied to 4f electrons in all Ce atoms. Our Pt- and Sn-containing models (see Supplementary Material) were based on a model stoichiometric ceria nanoparticle Ce_40_O_80_ ca. 1.5 nm large (Migani et al., 2010). Our previous calculations of this type of models resulted in several predictions subsequently confirmed experimentally (Bruix and Neyman, 2016, 2019). Each model nanoparticle was positioned in a rectangular periodically repeated cell of 2.2 × 1.9 × 1.9 nm. Spin-polarized Γ-point calculations of all model nanoparticles under scrutiny were performed with the single-point total energy convergence tolerance of 2 × 10^−5^ eV. Optimization of positions of all atoms constituting a given model was continued until the maximum forces acting on each atom were < 0.02 eV/Å.

## Results

### TEM Study of the As-Prepared by Plasma-Arc Synthesis PtCeC and PtCeSnC Composites

**Figure 1:**
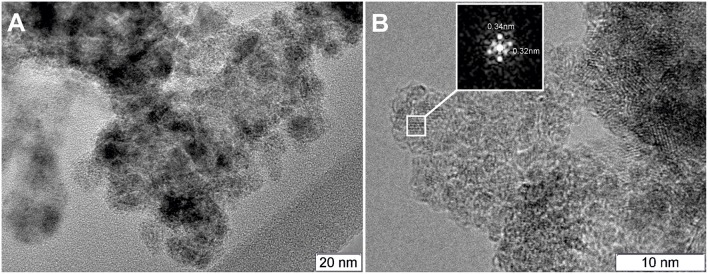
TEM image of the PtCeC composite **(A)** and Fourier image of the crystal lattice of a nanoparticle **(B)**.

The composite material PtCeC obtained by PAS consists of highly dispersed nanoparticles embedded within a matrix of amorphous carbon. Figure 1 shows typical TEM images of this material with 1 to 10 nm large nanoparticles. The analysis of interatomic distances features that nanoparticle structure corresponds to Ce_2_O_3_ (ICDD PDF-2 #00-023-1048). The reflections on the Fourier image displayed in Figure 1B reveal the interplanar distances of 0.32 and 0.3316 nm corresponding to the (002) and (100) reflections of a Ce_2_O_3_ trigonal structure. We detected a similar microstructure for a PdCeC composite prepared by PAS (Gulyaev et al., 2014b).

**Figure 2:**
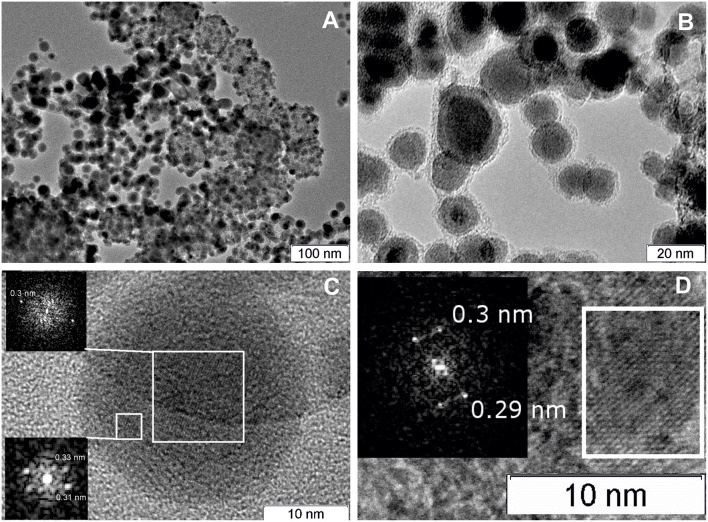
TEM image of the PtCeSnC composite **(A)**, TEM image of large core-shell-shell particles **(B)**. HRTEM image and Fourier image of the lattice of core-shell-shell particles Sn@SnO_2_@C **(C)**. HRTEM image and Fourier image of the crystal lattice of small Ce_2_O_3_ particles within amorphous carbon matrix **(D)**.

Morphology of the as-prepared PtCeSnC composite is characterized by the presence of two types of nanoparticles: a highly dispersed fraction with sizes 1–10 nm and a fraction of larger particles with sizes from 10 to 40 nm. Figure 2A shows typical images of the PtCeSnC composite.

Larger particles (see Figure 2B) exhibit a core-shell-shell structure. The outer shell has lower density than the core and is built of amorphous carbon. The core of the particles contains tin. The analysis of the crystalline structure of the inner particle core shows the distance 0.3 nm, which could be assigned to different phases: SnO_2_ (Pbcn) [0.30 nm (113)], SnO (P4/nmm) [0.299 nm (101)], Sn (I4_1_/amd) [0.29 nm (200)], see Figure 2C. The next crystalline shell of the particles shows the distances of 0.31 and 0.33 nm, which are unambiguously related to the (113) and (112) reflections of SnO_2_ (Pbcn). The observation of higher density of the particle core suggests that the core structure is related to Sn. Hence, a large fraction of the core-shell-shell particles appears to feature a composition Sn-SnO_2_-C, where C is amorphous carbon.

Structural analysis of the dispersed particles of the as-prepared PtCeSnC composite showed the distances of 0.3 nm and 0.29 nm, which could be related to (002) and (101) reflections of the Ce_2_O_3_ correspondently, see Figure 2D.

### Characterization of the Calcined Pt/Ce and Pt/CeSn Samples

Our earlier studies of catalysts prepared by PAS (Kardash et al., 2017) showed that calcination at temperatures above 600°C causes burning of carbon in PtCeC and PtCeSnC composites and transforms them into oxide materials. The chemical composition analysis was performed for the calcined samples (see Table 1). One can see that after calcination at 600–800°C the Pt/Ce sample exhibits a higher specific surface area than the Pt/CeSn sample. However, after calcination at 900°C the specific surface area of the Pt/CeSn sample becomes higher than that of the Pt/Ce sample, which indicates a stabilizing effect of Sn preventing particle sintering.

**Figure 3:**
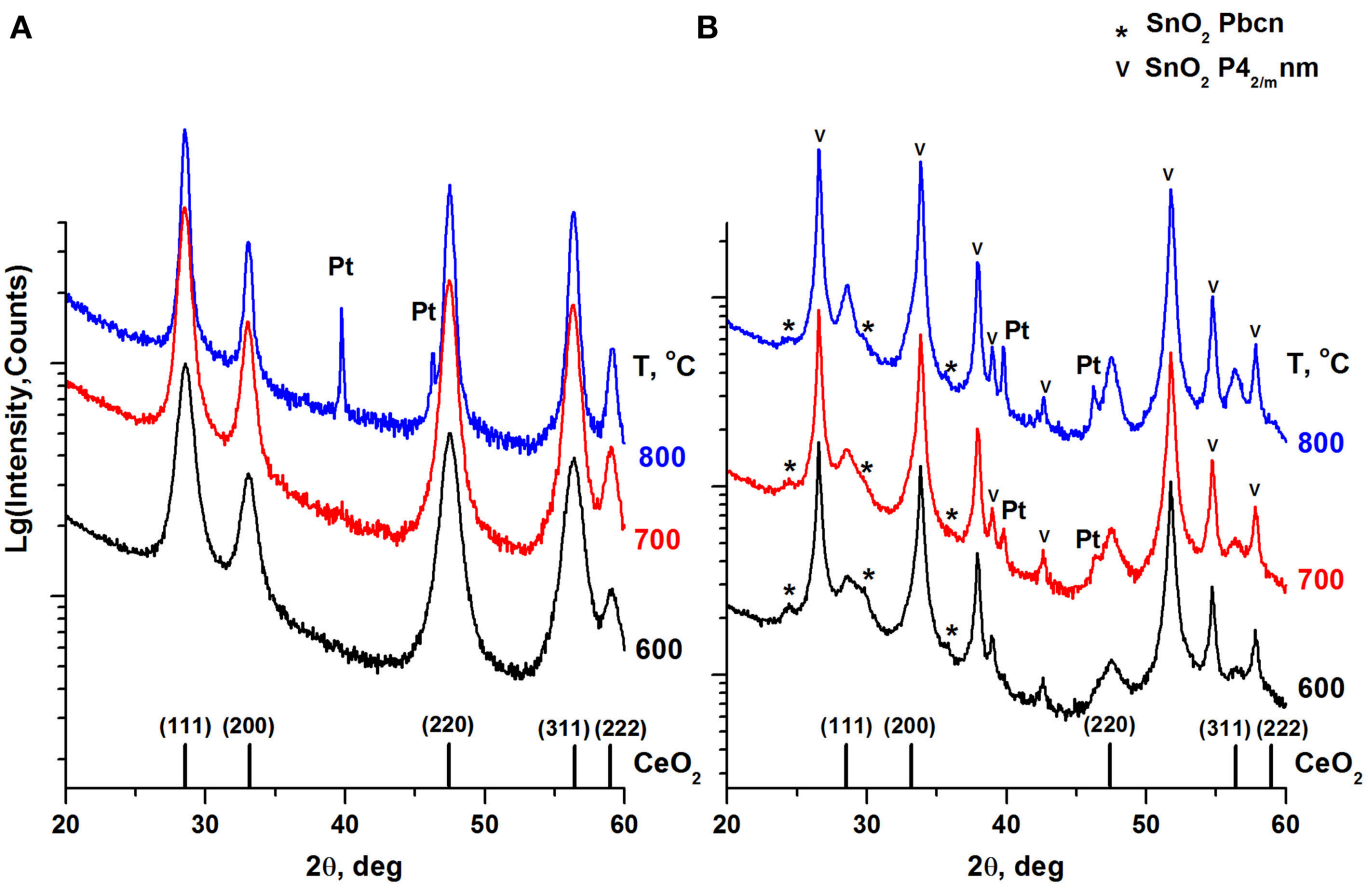
XRD patterns of the Pt/Ce **(A)** and Pt/CeSn **(B)** samples calcined at different temperatures. The graph at the bottom shows the positions of CeO_2_ fluorite phase reflections and corresponding Miller indexes. Symbol (^*^) indicates an unstable orthorhombic form of SnO_2_, the symbol (v) indicates reflections of a cassiterite SnO_2_ phase.

Figure 3 presents XRD patterns of the calcined Pt/Ce and Pt/CeSn samples. Table 2 shows the results of a quantitative phase analysis obtained by the Rietveld refinement of the XRD data.

**Table 2:** Phase composition of the Pt/Ce and Pt/CeSn samples calcined at different temperatures T_calc_, lattice parameter (a) and particle mean size (D) of the fluorite phase.

**Sample**	**T_**calc**_, °C**	**Phase composition, % wt**				**Parameters of the fluorite phase**	
		**CeO_**2**_ Fm3m**	**SnO_**2**_ P4_**2/**_mnm**	**SnO_**2**_ Pbcn**	**Pt**	**a, Å**	**D, nm**
Pt/Ce	600					5.415 (1)	6.6 (1)
	700	100	–	–	–	5.415 (4)	8.6 (1)
	800	98 (1)	–	–	2 (1)	5.413 (3)	12.7 (1)
Pt/CeSn	600	24 (7)	64 (6)	12 (3)	-	5.404 (1)	3.5 (5)
	700	20 (5)	70 (5)	9 (3)	0.4 (1)	5.401 (2)	3.0 (1)
	800	21 (4)	79 (4)	-	0.7 (1)	5.406 (2)	4.2 (1)

*The standard deviations of the calculated values are given in parentheses*.

CeO_2_ phase with a fluorite-type structure is a major component in the Pt/Ce sample. The lattice parameter of the fluorite phase and mean particle size estimated from the XRD data are shown in Table 2. For the Pt/CeSn sample, the oxides SnO_2_ (cassiterite, ICDD PDF-2 #00-034-0394) and CeO_2_ (ICDD PDF #00-029-1484) are the major phases. Note, that the CeO_2_ fluorite phase exhibits smaller particles in the Pt/CeSn sample compared to the Pt/Ce sample for all calcination temperatures. This observation implies that addition of SnO_2_ to the composite allows stabilizing the CeO_2_ particle size against sintering.

Additional peaks were detected in the Pt/CeSn sample, corresponding to the unstable orthorhombic SnO_2_ phase (ICDD PDF-2 #04-015-3275). According to the literature data, this form of SnO_2_ can be built at high pressures and temperatures (Gracia et al., 2007). The fraction of the orthorhombic SnO_2_ phase is decreased upon sample calcination. We assume that this unstable phase can result from a rapid oxidation of the PtSnCeC composites, when they are exposed to the atmosphere upon taking out of the synthesis chamber. This phase could be stabilized by the carbon matrix within core-shell-shell nanoparticles. However, the subsequent calcination of the composite transforms it into a stable cassiterite phase.

Metallic Pt phase was not detected in the samples calcined at 600°C. Peaks corresponding to metallic Pt appear on the XRD patterns only after calcination of the Pt/CeSn sample at 700°C and of the Pt/Ce sample at 900°C. However, the Rietveld refinement shows that the content of metallic Pt is lower than the analytical amount of Pt in the sample. This indicates the presence of Pt in highly dispersed states in the samples calcined at 600°C and even higher temperatures.

Total amount of SnO_2_ in the Pt/CeSn sample is less than detected by the XRF analysis. It implies that a part of Sn is embedded in the CeO_2_ lattice. This assumption is supported by decreasing of the fluorite lattice parameter from 5.411 Å for CeO_2_ in the Pt/Ce sample to 5.401 Å in the Pt/CeSn sample. Relying on the difference in the radii of the Ce^4+^/Ce^3+^ cations and Sn^4+^ cations, it is possible to estimate the composition of the Ce_1−x_Sn_x_O_y_ joint fluorite phase. For the Pt/CeSn-600 sample, the estimated composition of the fluorite phase is Ce_0.93_Sn_0.07_O_2−δ_. However, the so determined tin content in the fluorite structure is underestimated, since the calculation did not take into account that the fluorite lattice parameter can increase with decreasing particle size to 1–3 nm (Tsunekawa et al., 2004; Baranchikov et al., 2010).

**Figure 4:**
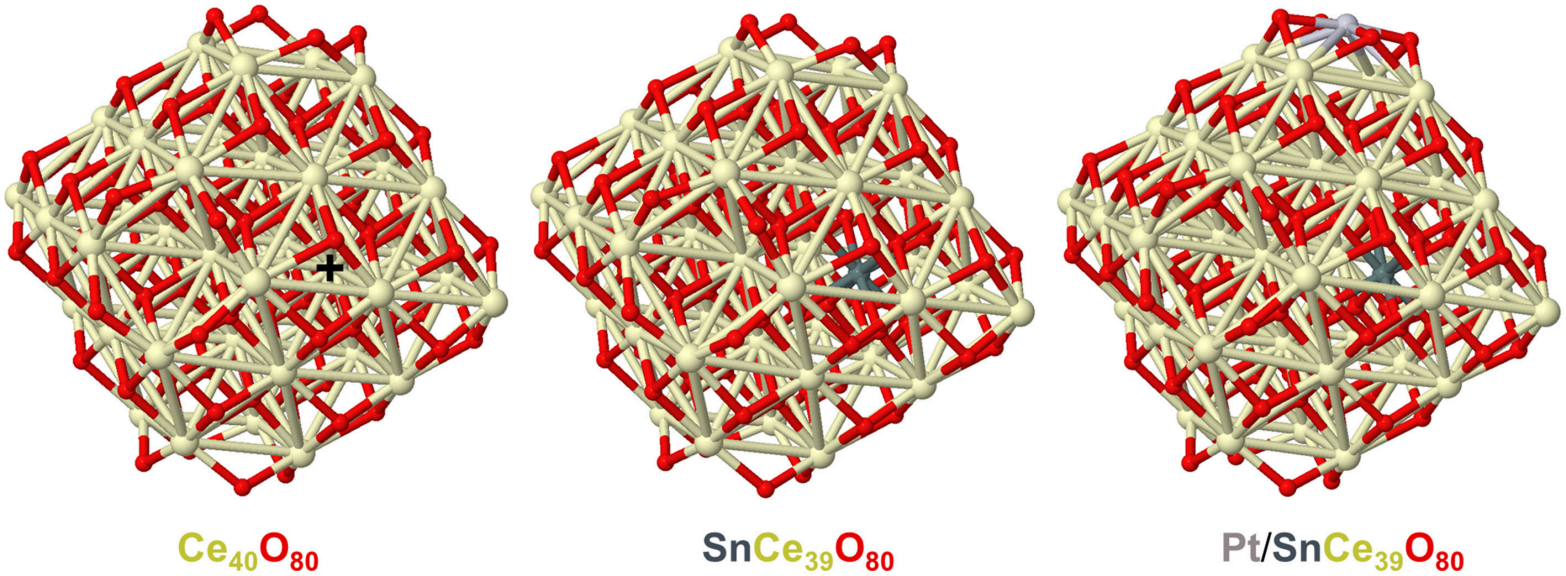
Geometric structures of CeO_2_-based nanoparticles calculated to analyze effects of doping Sn ions on the nearest distances from the latter to O and Ce ions. Color coding for atoms: Ce—beige, O—red, Sn—dark gray, Pt—light gray. The nearest distances to the Ce^4+^ ion marked by **+** in the Ce_40_O_80_ model: Ce-O-2.292, 2.292, 2.293, 2.295, 2.358, 2.358, 2.361, 2.361 (average 2.326); Ce-Ce-3.732, 3.734, 3.737, 3.738, 3.744, 3.746, 3.747, 3.752 (average 3.741). The nearest distances to the Sn^4+^ ion in the SnCe_39_O_80_ model: Sn-O-2.181, 2.184, 2.187, 2.189, 2.264, 2.269, 2.276, 2.280 (average 2.229); Sn-Ce-3.687, 3.687, 3.688, 3.693, 3.717, 3.724, 3.726, 3.730 (average 3.707). The nearest distances to the Sn^4+^ ion in the Pt/SnCe_39_O_80_ model: Sn-O-2.175, 2.176, 2.205, 2.210, 2.238, 2.246, 2.260, 2.266 (average 2.222); Sn-Ce-3.682, 3.691, 3.691, 3.700, 3.714, 3.717, 3.725, 3.726 (average 3.706). All the distances are in Å. Atomic coordinates and total energies of the displayed structures are given in the Supplementary Material.

In line with these considerations, the nearest 8 Sn-O and 8 Sn-Ce distances calculated by DFT for the model tin-doped nanoparticles SnCe_39_O_80_ and Pt/SnCe_39_O_80_ with Sn^4+^ dopant located in an inner Ce^4+^ position are shorter than the corresponding Ce-O and Ce-Ce distances in the pristine Ce_40_O_80_ particle (see Figure 4 and Supplementary Material). Namely, the average distances Ce-O and Ce-Ce in Ce_40_O_80_ are 2.326 Å and 3.741 Å, respectively, whereas the corresponding average distances Sn-O and Sn-Ce are 2.229 Å and 3.707 Å in the SnCe_39_O_80_ particle and 2.222 Å and 3.706 Å in the Pt/SnCe_39_O_80_ particle. Our calculations of the latter with the doping Sn occupying various cationic positions of the CeO_2_ lattice showed (see Supplementary Figure 1) that the location of the Sn^4+^ dopant inside the nanoparticle is moderately stabilized, by 14–36 kJ/mol, compared to its surface location on the (111) terrace of the nanoparticle. Interestingly, a notably more stable location was calculated for a surface corner position of the dopant, where it substitutes a partially reduced Ce^3+^ cation and acquires a quite unusual Sn^3+^ state. We note in passing that such special corner sites O_4_ exposed by nanostructured ceria are able to bind very strongly atoms of various metals in the form of cations (Figueroba et al., 2016, 2017; Neitzel et al., 2016).

**Figure 5:**
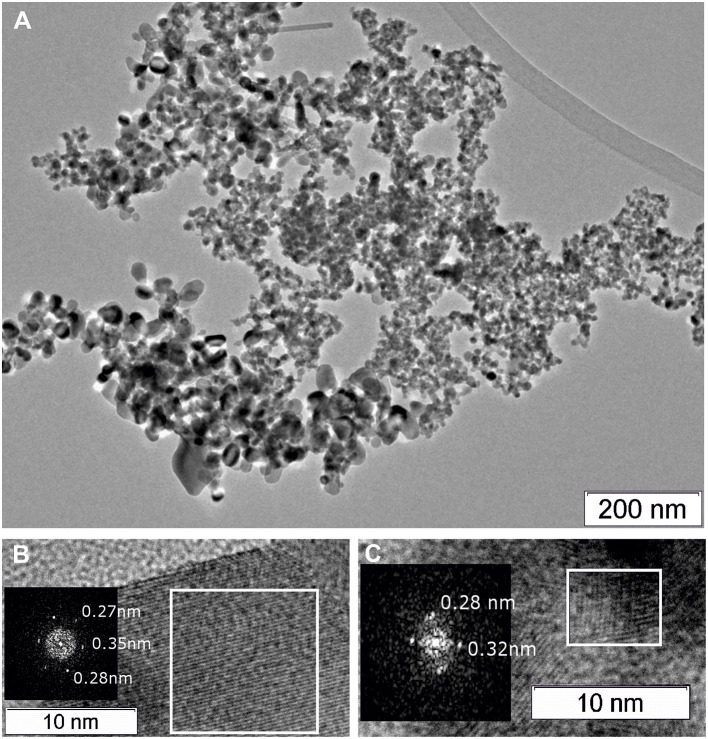
TEM images of the Pt/CeSn-600 sample **(A)**. A HRTEM and Fourier image of the crystal lattice of the SnO_2_ cassiterite phase **(B)**, HRTEM and Fourier image of the CeO_2_ fluorite phase **(C)**.

**Figure 6:**
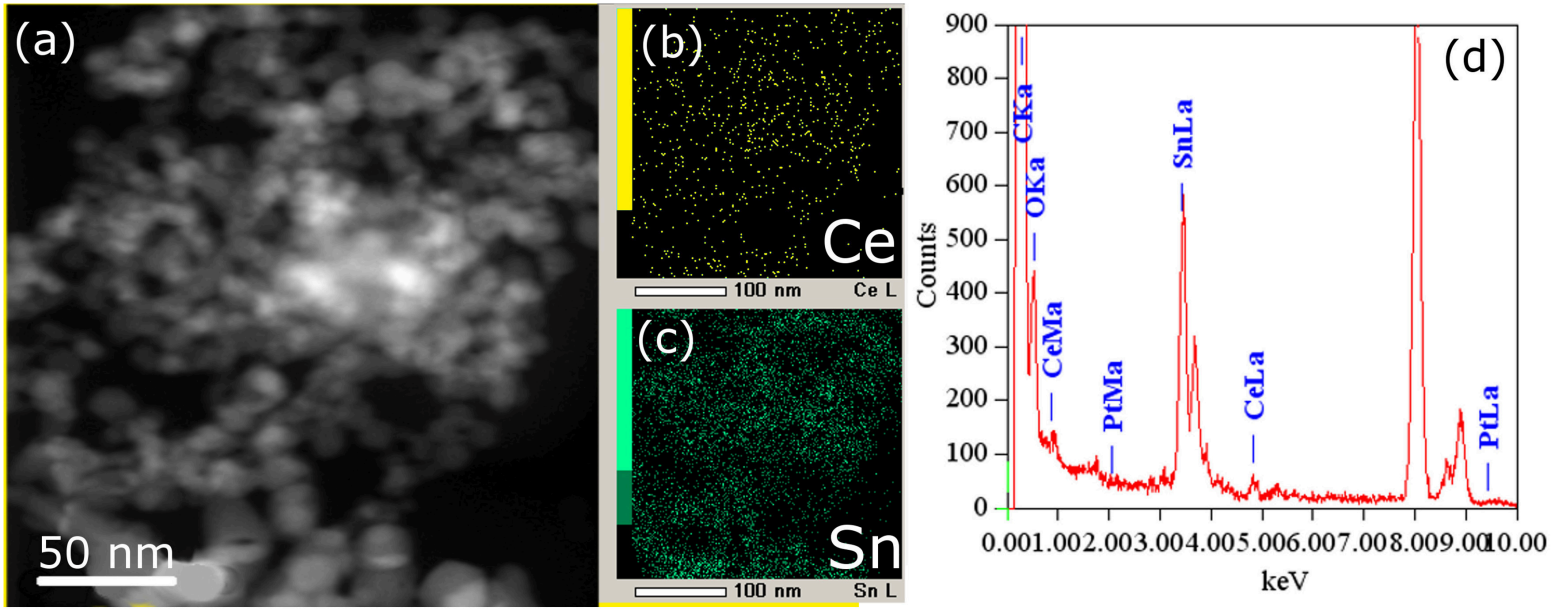
HAADF-STEM image **(a)** and corresponding EDX-mapping **(b,c)** patterns (CeL signal is shown in yellow, SnL signal is shown in green) of the Pt/CeSn-600 sample, **(d)** EDX analysis of the sample composition.

Figures 5, 6 show TEM images of the Pt/CeSn and Pt/Ce samples calcined at 600°C. The TEM data corroborate that calcination of the composites causes removal of the amorphous carbon matrix and crystallization of the formed oxide structures. Two particle types are observed in the Pt/CeSn-600 sample: larger ones with the sizes of 20–50 nm and smaller ones with the sizes of 5–15 nm (Figure 5A). A detailed analysis of the structure of the material showed that the larger particles belong to SnO_2_ (P4_2_/mnm) (Figure 5B). The crystal structure analysis of the smaller particles revealed that they correspond to a cubic fluorite-type structure (Figure 5C). These findings agree with the XRD analysis data showing that fluorite-type particles are smaller than those of tin oxides type.

The cerium and tin distribution in the Pt/CeSn-600 sample was investigated by means of EDX. Figure 6 displays HAADF-STEM images and the corresponding EDX maps for cerium and tin. As can be seen from the TEM images, cerium and tin are distributed over the entire volume of the sample, and no individual CeO_2_ particles were detected there. This is consistent with the XRD data for the Pt/CeSn-600 sample showing the formation of composites built from SnO_2_ and Ce_1−x_Sn_x_O_2_ nanoparticles. The EDX data on the sample composition agree with the chemical analysis data.

**Figure 7:**
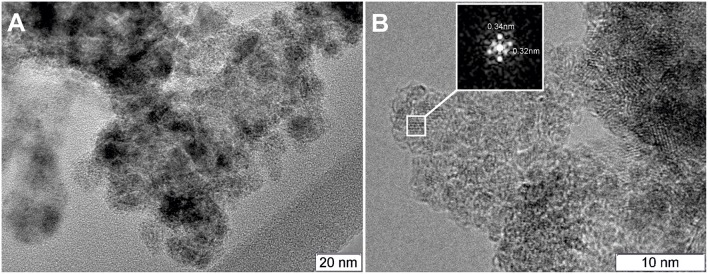
**(A)** TEM image of the Pt/Ce-600 sample, and **(B)** HRTEM and Fourier image of the CeO_2_ crystal lattice.

Figure 7 displays TEM images for the Pt/Ce-600 sample. Nanocrystalline blocks with sizes 5–10 nm are observed. The detected distances correspond to the cubic fluorite-type ceria.

**Figure 8:**
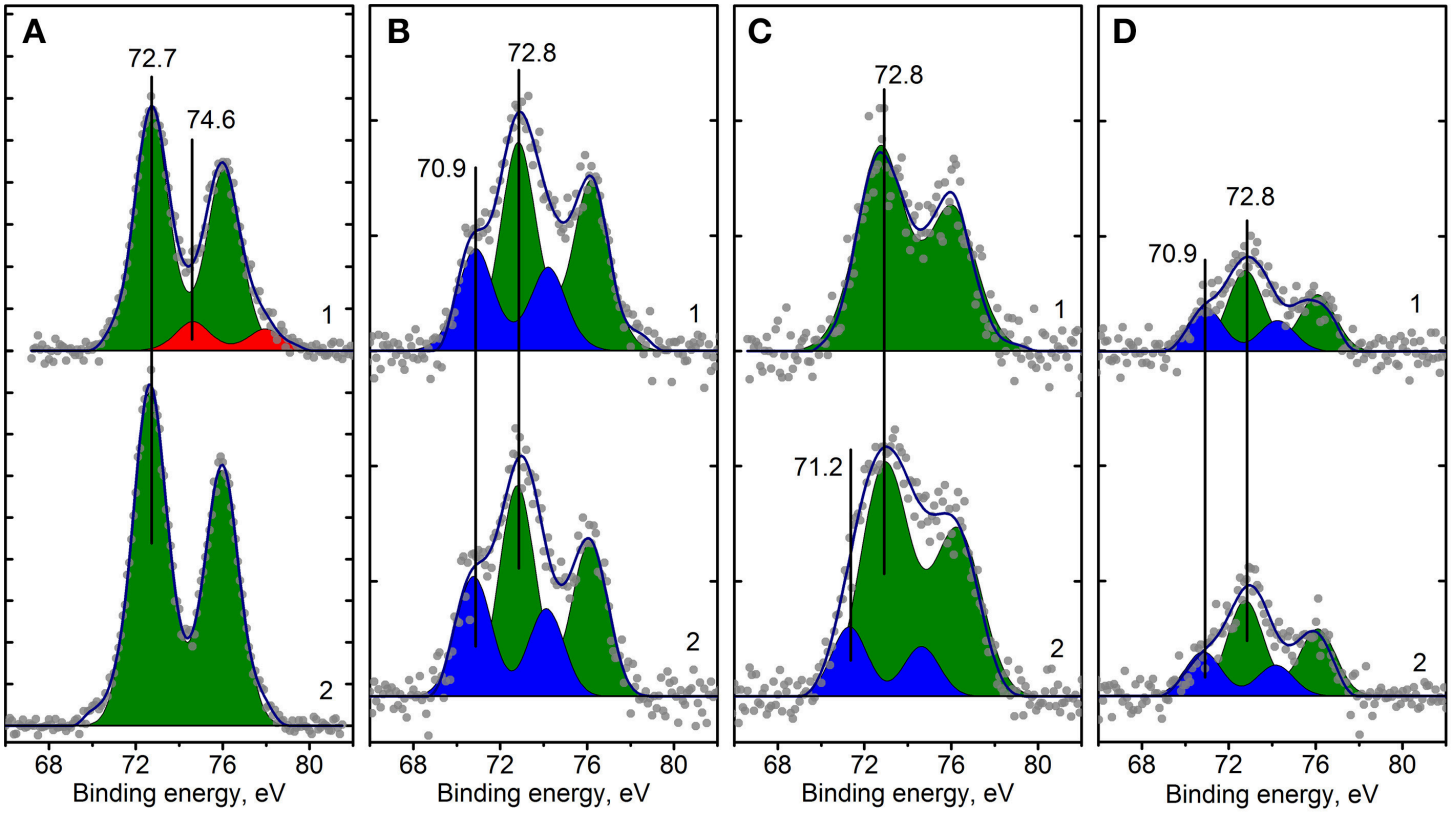
Curve fitted Pt4f XPS spectra for Pt/Ce-600 **(A)**, Pt/Ce-900 **(B)**, Pt/CeSn-600 **(C)** and Pt/CeSn-900 **(D)** samples, before (curves 1) and after CO+O_2_ reaction (curves 2). Ticks range on the Y-axis is 50 counts.

**Figure 9:**
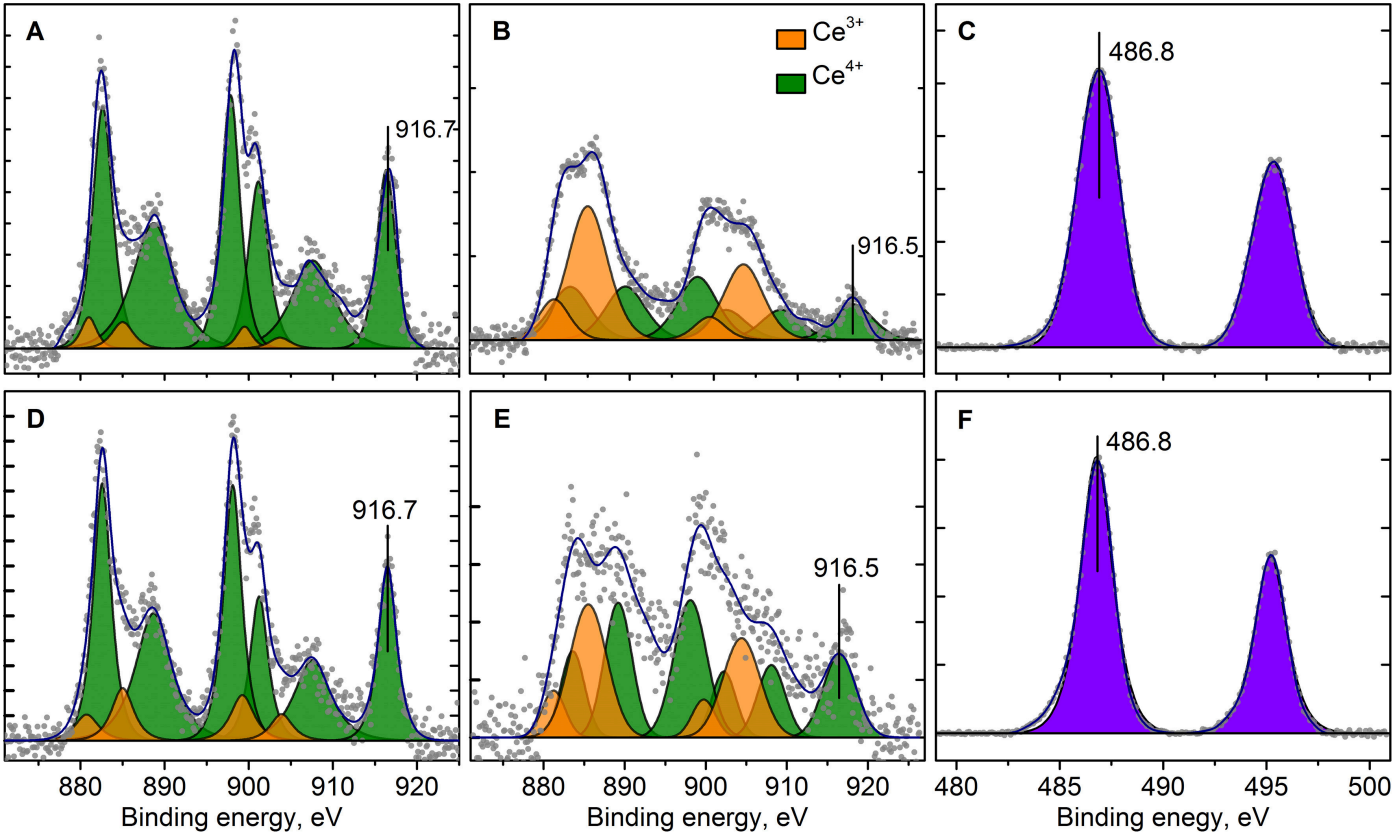
Curve fitted Ce3d **(A,B,D,E)** and Sn3d **(C,F)** XPS spectra for Pt/Ce-600 **(A,D)**, Pt/CeSn-600 **(B,C,E,F)** before **(A,B,C)** and after CO+O_2_ reaction **(D,E,F)** respectively. Ticks range on the Y-axis is 500 counts.

XPS spectra of the Pt/Ce and Pt/CeSn samples calcined at 600 and 900°C are shown in Figures 8, 9. The quantitative analysis data and the Pt4f binding energy values determined from the XPS spectra for the Pt/Ce-600 and Pt/CeSn-600 samples are presented in Table 3. According to these spectroscopic data all samples contain significant amounts of carbon, concentration of which varies from 27 to 35 at. %. From the analysis of the C1s spectra (not shown) it follows that the detected carbon is represented mainly by amorphous carbon or hydrocarbon groups. The share of oxygen-containing carbon groups, including carbonates, is at most 5–10% (Table 3). Such notable content of carbon in the samples obtained by sputtering in an electric arc discharge is not surprising, since the sputtering of atomically dispersed metals and species from graphite carbon rod occurs simultaneously.

**Table 3:** Quantitative composition of the catalyst surface and the XPS binding energy values E_b_ of individual states of the elements in the Pt/Ce-600 and Pt/CeSn-600 catalysts.

**Sample**	**Pt/Ce**		**Pt/CeSn**	
	**E_**b**_ (eV)**	**Concentration, % at**.	**E_**b**_ (eV)**	**Concentration, % at**.
Pt4f		0.65		0.26
Pt^2+^	72.7	0.58	72.7	0.26
Pt^4+^	74.6	0.07	–	–
C1s		34.83		27.56
C_1_	284.8	30.42	285.5	25.88
C_2_	288.6	4.41	289.0	1.68
Sn3d	–	0	486.9	18.62
Ce3d	916.7	24.85	916.5	7.30
O1s	529.4	39.67	530.9	46.25
Ce/Sn		100:0		28:72
Ce^3+^/Ce (%)		6		49

Analysis of the charge states of platinum in a series of samples was carried out using the spectra curves fitting. Fitted Pt4f spectra are presented in Figure 8. Upon decomposition into individual components, metallic platinum (E_b_ = 71.1 eV) could not be detected in samples calcined at 600°C. In the Pt/Ce-600 sample platinum is present in two states characterized by E_b_(Pt4f_7/2_) = 72.7 and 74.5 eV. Formally, these states can be referred to as Pt^2+^ and Pt^4+^. In the remaining samples in the presence of tin, only the state Pt^2+^ with E_b_(Pt4f_7/2_) = 72.8 eV was detected, but not the state Pt^4+^.

After calcination at 900°C, the Pt^2+^ species are preserved. However, a new state with E_b_(Pt4f_7/2_) = 70.9 eV appears. This state is referred to as Pt^0^, and its appearance is in a good agreement with XRD data. After calcination at 900°C, the surface Pt concentration decreases, which could be attributed to sintering of Pt particles.

Figure 9 shows Ce3d spectra for the Pt/Ce-600 and Pt/CeSn-600 samples, and Sn3d for the Pt/CeSn-600 sample. The Sn3d spectra show only one main doublet with E_b_(Sn3d_5/2_) = 486.9 eV attributable to Sn^4+^ (Moulder et al., 1992). Ce3d spectra were decomposed into individual components of Ce^4+^ species (V, V^′′^, V^′′′^ and U, U^′′^, U^′′′^) and Ce^3+^ species (V^0^, V′ and U^0^, U′) in accordance with literature data (Anandan and Bera, 2013; Zhu et al., 2014). The analysis shows that the quantity of Ce^3+^ species is increased significantly in the Pt/CeSn-600 sample. In the Pt/Ce-600 sample, only 6 at.% of Ce^3+^ is detected, whereas 49% of cerium is in the Ce^3+^ state in the Pt/CeSn-600 sample.

Such a high amount of Ce^3+^ species in the Pt/CeSn-600 sample could be explained by the XRD and TEM data, which show the formation of the Sn-doped fluorite phase Ce_1−x_Sn_x_O_2−δ_. When Sn^4+^ substitutes cerium in the fluorite phase, the Ce^3+^ in the position of Ce^4+^ could be generated together with an oxygen vacancy according the electroneutrality requirement: CeO_2_ → Sn_Ce_ + V${}_{\text{O}}^{••}$ + 2Ce'_Ce_, where V^••^ – is an oxygen vacancy with −2 charge, Ce'_Ce_–is a Ce^3+^ in the Ce^4+^ position.

**Figure 10:**
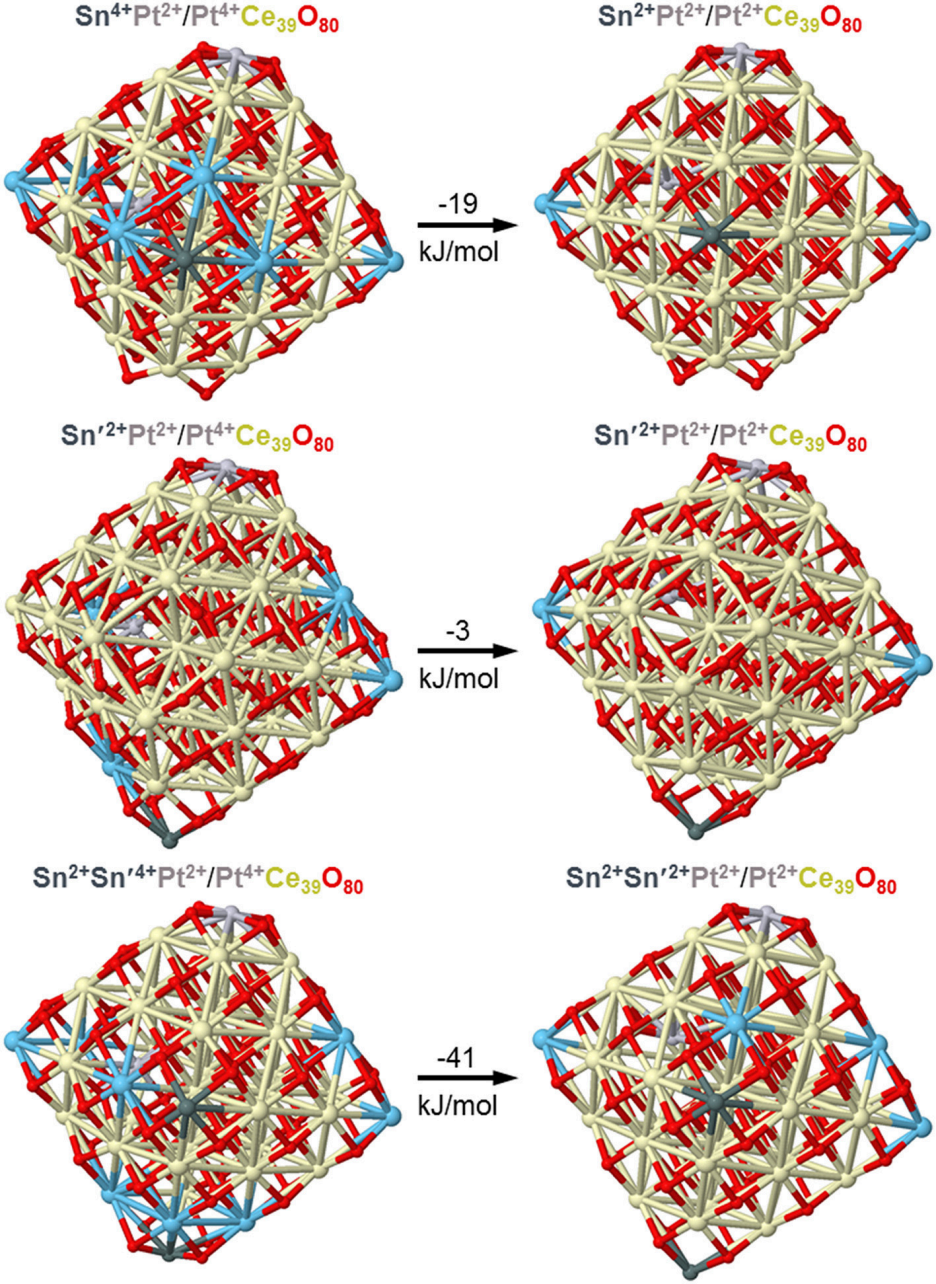
DFT optimized structures and relative energies of model Pt/PtCe_39_O_80_ nanoparticles interacting with one and two Sn atoms illustrating interplay of Pt^4+^ and Pt^2+^ oxidation states. Color coding for atoms: Ce^4+^-beige, Ce^3+^–light blue, O—red, Sn—dark gray, Pt—light gray. Atomic coordinates and total energies of the structures are given in the Supplementary Material.

Sn atoms were shown in a combined experimental and DFT study to easily reduce ceria nanoparticles, both bare and doped by Pt atoms acquiring the oxidation state +2 (Lykhach et al., 2017b). First, only Ce^4+^ cations were partially reduced upon interactions of atomic Sn with Pt/ceria nanoparticles, but already a moderate increase of Sn content resulted in the reduction of cationic Pt^2+^ species to essentially neutral atomic ones prone to form Pt clusters. Using the same computational approach and similar model Pt/ceria nanoparticles we computationally investigated in the present work stability of Pt dopants in a much more labile +4 state in ceria nanostructures in the presence of minimal amounts of Sn. Figure 10 shows that the interaction with a model particle Pt^2+^/Pt^4+^Ce_39_O_80_ of just one Sn atom can already slightly favor partial reduction of Pt^4+^ remote from Sn by 8 Å. In the presence of two doping Sn atoms (see Figure 10, bottom panels) we also succeed to optimize a local-minimum structure Sn_2_Pt^2+^/Pt^4+^Ce_39_O_80_ with the inner Pt atom in the highest oxidation state +4. This structure is over 40 kJ/mol less stable than the corresponding structure Sn_2_Pt^2+^/Pt^2+^Ce_39_O_80_, where Pt^4+^ is reduced to Pt^2+^. These calculated data substantiate and explain our experimental observations on the absence of the Pt^4+^ XPS signals, when Sn component is added to the Pt-ceria samples.

### Catalytic Properties and TPR-CO Data

**Figure 11:**
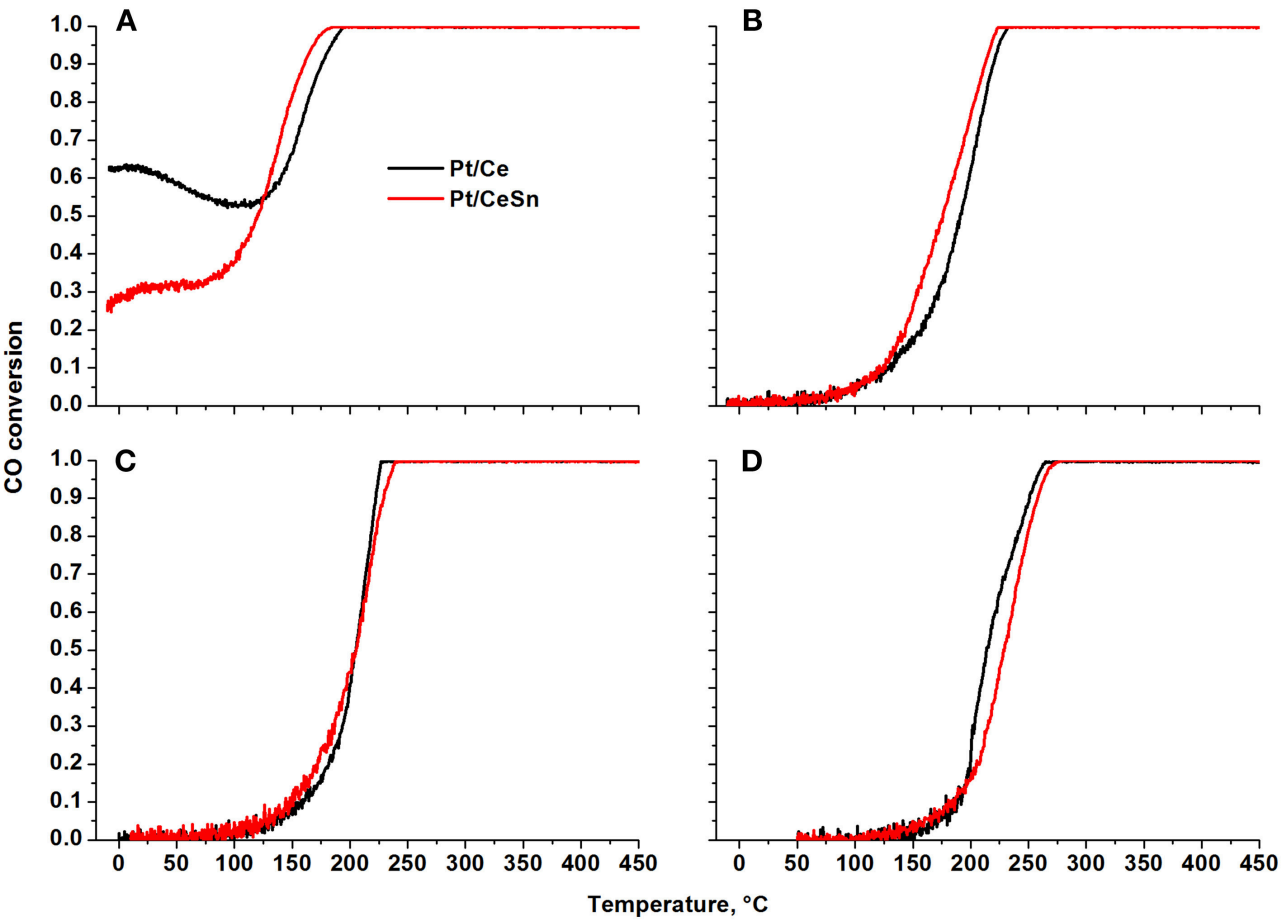
Temperature dependencies of CO conversion (TPR-CO+O_2_) for the Pt/Ce and Pt/CeSn catalysts calcined at different temperatures: **(A)** 600°C, **(B)** 700°C, **(C)** 800°C, and **(D)** 900°C.

Figure 11 presents temperature dependencies of the CO conversion over the prepared Pt/Ce and Pt/CeSn catalysts and Table 4 shows temperatures for the 10% (T_10_) and 50% (T_50_) CO conversion.

**Table 4:** Catalytic characteristics in CO oxidation of the samples calcined at different temperatures T_calc_.

**T_**10**_, T_**50**_, °C**	**T_**calc**_, °C**	**Pt/Ce**	**Pt/CeSn**
T_10_, °C	600	< -20	< -20
	700	126	122
	800	159	146
	900	182	182
T_50_, °C	600	< -10	118
	700	191	175
	800	204	205
	900	215	228

The Pt/Ce and Pt/CeSn catalysts, calcined at 600°C (Figure 11A), are characterized by unusual behavior of CO conversion at varying temperature. Up to 100 °C the observed CO conversion either quite weakly depends on temperature (Pt/CeSn) or even decreases with temperature increase (Pt/Ce). The conversion of CO at −10°C is 63 and 26% for Pt/Ce and Pt/CeSn, respectively. The temperature of the 10% CO conversion is significantly below room temperature (Table 4).

The presence of several regions in the CO conversion curve with different temperature dependencies, i.e., with different observed activation energies, indicates the presence of active centers of different nature triggering different CO oxidation mechanisms.

The calcination of catalysts at temperatures above 700°C leads to the disappearance of the low-temperature activity. The T_10_ values increase by more than 150°C. The 50% conversion of CO (T_50_) increases by 215 and 110°C for Pt/Ce and Pt/CeSn, respectively after calcination of the catalysts at 800°C. Catalysts calcination at 900°C results in the increase of T_50_ by 10°C for Pt/Ce and by 20°C for Pt/CeSn catalysts.

The TOF values at 150°C are 0.005 and 0.020 for the Pt/Ce-700 and Pt/CeSn-700 samples, respectively (see Table 5). Furthermore, after calcination at 800 and 900°C the Pt/CeSn samples show higher TOF values than the Pt/Ce samples. These results indicate that the promotion by tin leads to an increase in both thermal stability and catalyst activity in the medium temperature range.

**Table 5:** TOF and activation energy E_a_ values for CO oxidation over the Pt/Ce and Pt/CeSn samples calcined at 700, 800, and 900°C and featuring specific surface area S_BET_.

**Sample**	**Pt,%**	**S_**BET**_, m^**2**^/g**	**E_**a**_, kcal/mol**	^*^TOF × 10^**3**^, s^**−1**^
Pt/Ce-700	4.58	71	8.3	5.4
Pt/CeSn-700	2.37	39	9.2	19.8
Pt/Ce-800	4.58	48	10.8	1.5
Pt/CeSn-800	2.37	27	11.3	12.8
Pt/Ce-900	4.58	10	17	0.7
Pt/CeSn-900	2.37	16	12.7	7.5

* *TOF(s^−1^) = W/C_Pt_, where W—reaction rate (molecules/cm^2^ s), C_Pt_–Pt concentration calculated from Pt content in the catalyst (XPS)*.

Table 5 also provides activation energy values calculated for CO oxidation reactions on Pt/Ce and Pt/CeSn catalysts calcined at 700, 800, and 900°C. The finding that the activation energy values are quite close to each other indicates that the limiting stages of the CO oxidation catalytic process on different samples under scrutiny are the same and, therefore, the oxidation process is carried out at essentially the same active centers. The data given in Table 5 was obtained at the temperature range of 100–200°C. It was not possible to get such quantitative data for the low-temperature range of 0–100°C. Nevertheless, it can be assumed that the reaction mechanism is similar in the low-temperature range and is triggered by active centers of the fluorite phase.

The catalysts after CO+O_2_ reaction were analyzed by XPS. Figure 8 (curves 2) shows Pt4f lines for the Pt/Ce-600, Pt/Ce-900, Pt/CeSn-600, and Pt/CeSn-900 catalysts after reaction. For the most active catalysts, calcined at 600°C, XPS shows that the fraction of Pt^2+^ ionic species in both Pt/Ce-600 and Pt/CeSn-600 catalysts does not decrease significantly during the catalytic tests. However, we observe some reduction of Pt after the tests: Pt^4+^ species are reduced to Pt^2+^ in the Pt/Ce-600 catalysts; a small fraction of Pt metal is observed in the Pt/CeSn-600 catalysts. This is an indication that some Pt cations are reduced in CO+O_2_ mixture. Yet, most of Pt^2+^ is preserved after the catalytic tests. Furthermore, no changes in the Pt states were detected in the samples calcined at 800 and 900°C. Figures 9D–F shows that Ce and Sn oxidation states of the catalysts are not changed too. This observation was expected, since the catalytic reaction CO+O_2_ is carried out not in a stoichiometric mixture, but in an excess of oxygen (O_2_: CO ratio = 5: 1).

**Figure 12:**
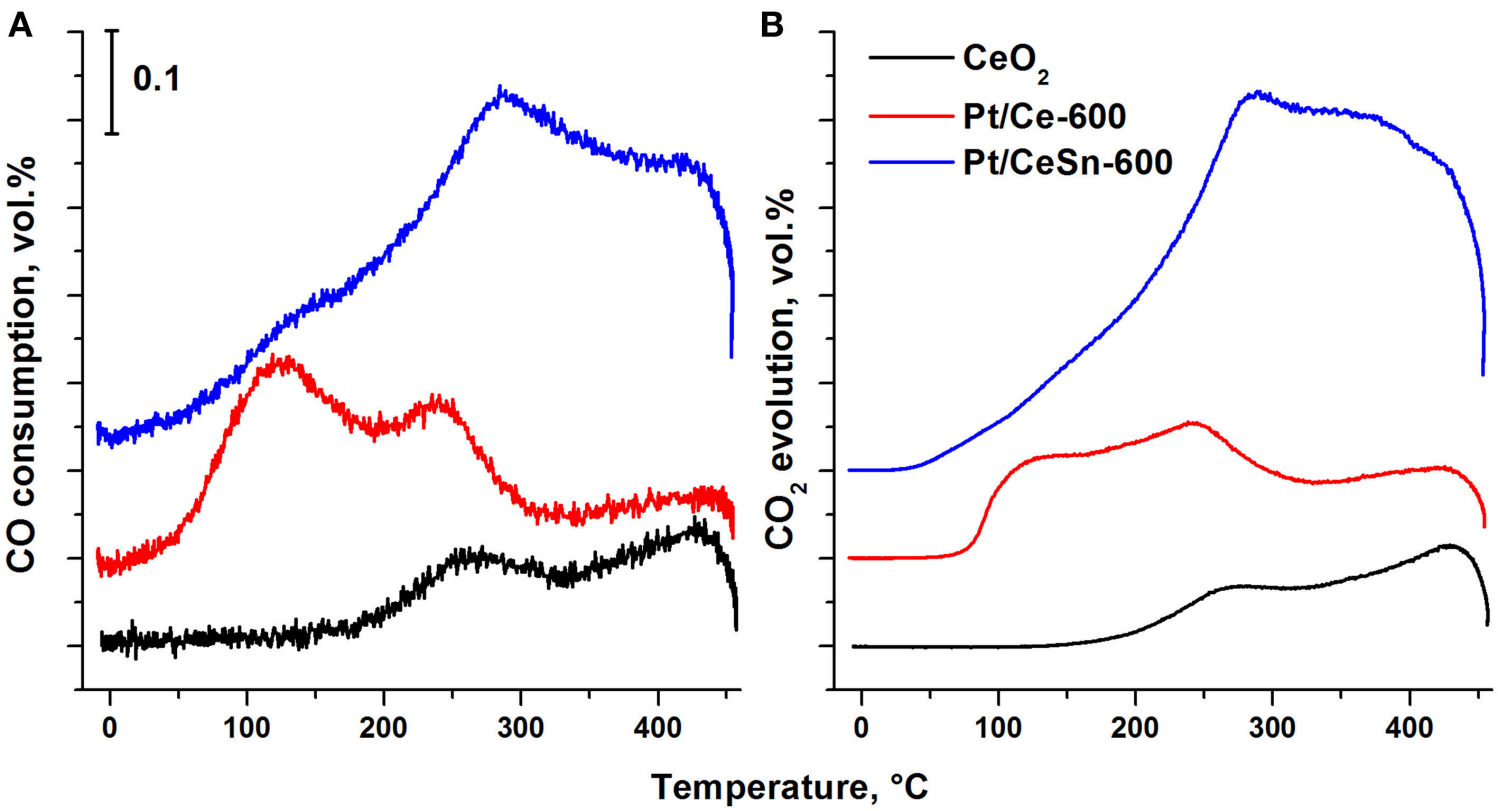
Temperature dependence of the CO consumption **(A)** and CO_2_ evolution **(B)** during the TPR-CO for the catalysts Pt/Ce-600, Pt/CeSn-600 and pristine CeO_2_-600.

Figure 12 presents results for the catalysts obtained by the method of temperature-programmed reaction with CO (TPR-CO). The study of our catalysts by the TPR-CO method in the absence of the gas-phase oxygen allowed us to characterize the reactivity of oxygen present in the catalysts to carbon monoxide. Spectra of both CO absorption and CO_2_ emission during TPR-CO are presented for the Pt/Ce, Pt/CeSn and pristine CeO_2_ samples calcined at 600°C.

For the pristine CeO_2_, CO consumption and CO_2_ release begin above 200°C. Addition of Pt leads to a strong activation of oxygen present in ceria revealed by lowering initial temperatures of the CO consumption and the CO_2_ release to just 20 and 45°C, respectively. The promotion by tin causes a further decrease of the reactions begin temperature by 20°C, i.e., to 0 and 25°C, respectively. These data suggest that the addition of tin to the catalysts leads to weaker binding of the lattice oxygen in them. TPR-CO spectra of the catalysts under study reveal three peaks at the temperatures of 20–200°C (peak 1), 200–350°C (peak 2) and above 350°C (peak 3). The first two peaks appear to result from CO interactions with reactive oxygen activated during formation of the Pt—Ce catalyst due to appearance of ionic platinum in the (PtCe)O_2_ mixed phase.

At temperature above 350°C the profiles of CO consumption and CO_2_ release match. According to Giordano et al. (2000), diffusion of oxygen from the support volume to the surface and interaction with gas-phase CO begins at these temperatures. H_2_ release is observed in our experiments during such a process. It was suggested (Zhu et al., 2004) that H_2_ and CO_2_ release could be observed during the TPR-CO experiments due to a reaction of CO with surface hydroxo-groups of the catalyst: CO(ads) + 2OH^−^(support) → CO_2_(gas) + H_2_(gas) + O^2−^(support).

Addition of Sn to the catalysts leads to a decrease in the area of the peak 1 due to a decrease in the CeO_2_ content. The areas of the peaks 2 and 3 are increased due to increased amount of reactive oxygen species. Table 6 shows that the amount of consumed CO and released CO_2_ for the Pt/CeSn-600 catalyst is almost 3 times higher than that for the Pt/Ce-600 catalyst. This corroborates that the modification of our Pt/Ce catalyst by tin noticeably increases the amount of oxygen reactive at low temperatures in the Pt/CeSn catalyst. Notably, the catalysts calcined at temperatures above 600°C lose their high low-temperature oxidative activity. However, the observation of higher TOF values for Pt/CeSn catalysts compared to the corresponding Pt/Ce catalysts prepared by calcination at the same temperatures indicates increased thermal stability of the Sn-containing catalysts.

**Table 6:** CO consumption and CO_2_ evolution during the TPR-CO.

**Sample**	**CO, μmol/g**	**CO_**2**_, μmol/g**	^*^CO/Pt	^*^CO_**2**_/Pt
Pt/Ce-600	1,121	975	4.8	4.2
Pt/CeSn-600	2,753	3,093	23.8	26.7

* *CO/Pt and CO_2_/Pt are molar ratios of the CO consumed and CO_2_ evolved during the TPR-CO to the Pt content in the catalyst, respectively*.

## Discussion

To achieve effective low-temperature oxidation of CO over ceria-based catalysts particle size in them should not exceed 10 nm (Boronin et al., 2009; Kurnatowska et al., 2012). In such cases a SMSI effect between dispersed ceria and ionic forms of the active component is possible, which can result in the formation of a large number of oxygen vacancies and mobile oxygen species with increased diffusion and reactivity.

In this work we used plasma arc synthesis to obtain highly dispersed ceria particles and mixed phases with tin dioxide. TEM data show that the as-prepared condense PtCeC and PtCeSnC substances are nanocomposites, in which the Ce and Sn components are in the form of semi-oxidized particles with Ce_2_O_3_, SnO, and SnO_2_ crystalline structures coated with amorphous carbon. However, according to the reducing conditions of the synthesis, the initial particles should be in metallic state. We assume that the semi-oxidized nanoparticles Ce_2_O_3_ and SnO are formed, when samples are exposed to the atmosphere after opening the chamber, in which plasma arc synthesis was performed. Considering high reactivity of cerium and tin metal particles their partial surface oxidation occurs even despite the diffusion difficulties of oxygen molecules into the carbon matrix. As a result, formation of core-shell structures Ce_2_O_3_@C in the PtCeC composite and of core-shell-shell structures Sn@SnO@C in the PtCeSnC composite takes place.

Subsequent calcination of the nanocomposites in an oxygen atmosphere at T > 600 °C results in burning out of the amorphous carbon matrix and conversion of the components into oxidized forms. Our TEM and XRD data show that phases with a fluorite structure (CeO_2_ and Sn-doped CeO_2_) and cassiterite (SnO_2_) are formed, with fluorite particles being much smaller (3–4 nm) than SnO_2_ particles (9–12 nm). We note that so small CeO_2_ particles were not obtained in our previous studies of samples prepared by co-precipitation and solution-combustion methods (Slavinskaya et al., 2015; Vasilchenko et al., 2016). Our XRD data reveal formation of a mixed Ce_1−x_Sn_x_O_2−δ_ phase. Furthermore, XPS data show that the state of Pt in the Pt/CeSn-600 catalysts is characterized only by Pt^2+^ ions, which significantly differs from the state of platinum in the Pt/Ce-600 catalyst (Pt^2+^/Pt^4+^) and also indicates the influence of tin ions on the surface state of the fluorite phase in Sn-modified catalysts. Our DFT calculations showed that the presence of Sn^4+^ ions in ceria nanoparticles facilitates Pt^4+^ reduction to Pt^2+^. In addition, very stable square planar structures PtO_4_ were formed (Bruix et al., 2014; Figueroba et al., 2017) when platinum interacted with nanostructured CeO_2_ surface. It can be speculated that the presence of embedded tin ions boosts formation of such stable structures. Along with the effect of tin on the state of platinum, the Ce3d spectra show a significant increase in the concentration of Ce^3+^ ions. Namely, the concentration of Ce^3+^ ions in the Pt/CeSn catalysts reaches 50 % at., while in the Pt/Ce catalysts the Ce^3+^ concentration is 15–20% at. These data indicate that the number of oxygen vacancies in the Sn-modified catalysts is significantly higher.

Key factor for the implementation of efficient CO oxidation in PGM-ceria systems is the formation of mobile highly reactive oxygen species. TPR-CO experiments allowed us to establish the effect of mobile oxygen formation in the catalysts synthesized by PAS. For these catalysts, the starting temperature of the interaction with CO is 20–50°C. At temperatures up to 200°C a significant amount of oxygen is released due to interaction with CO. This low-temperature mobile oxygen is generated in both Pt/Ce and Pt/CeSn catalysts, but there is a difference. In fact, a significant part of mobile oxygen is released from the Pt/Ce-600 catalyst with a peak with T_max_ = 100°C, while from the Pt/CeSn-600 catalysts mobile oxygen is released in the whole temperature range from 20 to 450°C, and its amount is more than 2.5 times higher than in the case of Pt/Ce-600 catalyst. Obviously, this effect is directly related to the modification of the CeO_2_ fluorite phase by Sn^4+^ ions.

**Figure 13:**
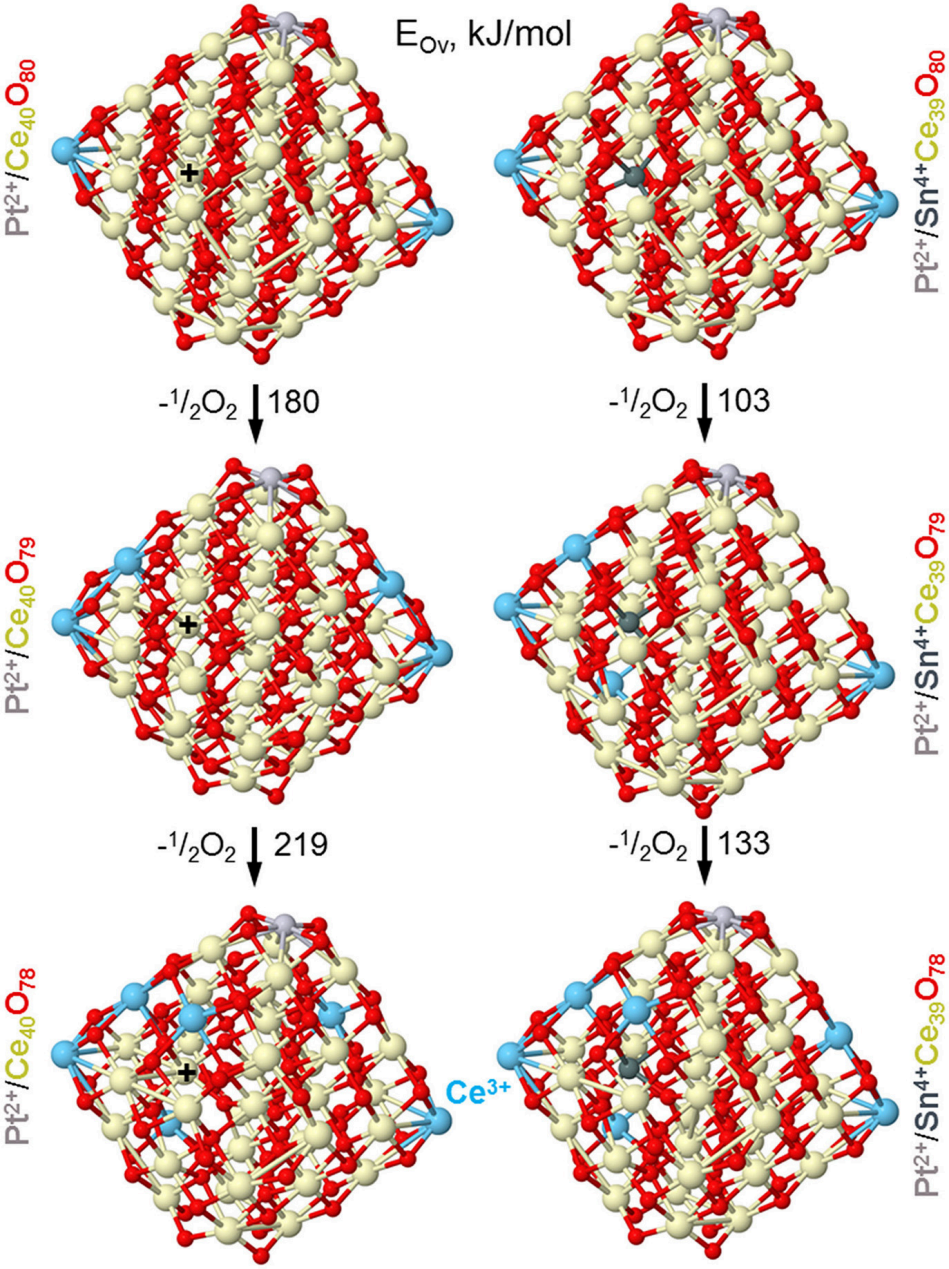
Optimized structures of nanoparticles Pt/Ce_40_O_80−n_ and Pt/SnCe_39_O_80−n_ corresponding to formation of the first and second oxygen vacancies (Ov) with the lowest energy E_Ov_ in the vicinity of Sn^4+^ and Ce^4+^ ions located in the same near-surface cationic position labeled by +. Color coding for atoms: Ce^4+^–beige, Ce^3+^–light blue, O—red, Sn—dark gray, Pt—light gray. Atomic coordinates and total energies of the structures are given in the Supplementary Material.

Our DFT modeling allows estimating effects of Sn^4+^ doping ions in near-surface Ce^4+^ positions of ceria nanoparticles on the reducibility of the latter (see Figure 13). In line with experimental observations (*vide supra*), the substitution of a Ce^4+^ cation in the lattice of ceria by Sn^4+^ greatly facilitates formation of oxygen vacancies O_v_ in the immediate vicinity of the dopant. Indeed, the lowest vacancy formation energy E_Ov_ (calculated with respect to ½ O_2_) for removal of the first O atom located near Sn^4+^ is almost 80 kJ/mol smaller than when the vacancy is created near Ce^4+^ cation occupying the same site. Calculated E_Ov_ value for creating the second O_v_ around the Sn^4+^ cation decreases even somewhat more with respect to the Ce^4+^ site. Notably, both the first and the second E_Ov_ values for the Sn^4+^ dopant case are very low, 103 and 133 kJ/mol, respectively. These E_Ov_ values are close to those calculated using the same DFT scheme for Pt-ceria nanocomposites, where predicted high oxygen mobility was confirmed experimentally (Vayssilov et al., 2011). Thus, the present low E_Ov_ values corroborate very high oxygen mobility in nanostructured ceria induced by doping with Sn. Our calculations indicate that at least two mobile reactive O atoms can be easily generated by the presence of one Sn^4+^ cation in ceria lattice, with concomitant creation of four reduced Ce^3+^ ions, in full agreement with our experimental observations. The enhanced mobility of oxygen is related to strongly distorted oxygen coordination of Sn^4+^ compared to Ce^4+^ most favorably surrounded by cubes of eight O^2−^ anions. The calculated eight nearest Ce-O and Sn-O distances (in Å) are: for Ce^4+^ in Pt^2+^/Ce_40_O_80_−2.28 × 2, 2.31 × 2, 2.33, 2.34, 2.35, 2.36 and for Sn^4+^ in Pt^2+^/SnCe_39_O_80_−2.11, 2.12, 2.14, 2.15, 2.22, 2.27, 2.35, 2.54 (see sketches of these models in the upper panels of Figure 13 and atomic coordinates in the Supplementary Material).

Although the amount of mobile oxygen is generally higher for the Pt/CeSn-600 catalyst, this is probably not the only important factor for low-temperature oxidation reactivity. Our CO conversion data show that the catalytic activity in the range of 0–100°C is slightly lower for the Pt/CeSn-600 catalysts than for the Pt/Ce-600 ones. Most likely, the amount of mobile oxygen affects the catalytic activity above 100°C, when the TOF values are higher for the Pt/CeSn-800 and Pt/CeSn-900 catalysts. The six-fold difference in TOF values for the Pt/Ce and Pt/CeSn catalysts calcined at 900°C reinforces notably higher activity of the latter at temperatures above 100°C. According to the experimental data, Pt^2+^ and Pt^4+^ ions are present in catalytically active centers of the Pt/Ce-600 samples. In the Pt/CeSn-600 samples, doping Sn species can replace Pt^4+^ ions and also induce additional reduction of Ce^4+^ ions.

Application of plasma arc synthesis makes it possible to mix catalysts components at the cluster and nanoscale level. Due to the mixing of components takes place in the carbon matrix, which suppresses sintering at the sputtering stage, formation of highly dispersed oxide structures during annealing in oxygen atmosphere is possible. In the processes of annealing and burning of carbon, nanoparticles of CeO_2_ and SnO_2_ interact in the contact area.

## Conclusions

We prepared complex PtCeC and PtCeSnC composites in the form of highly defective oxide particles stabilized in carbon matrices using a plasma arc synthesis method. High-temperature annealing of the prepared samples in oxygen removes the carbon matrix and results in the formation of Pt/CeO_x_ and Pt/CeSnO_x_ samples that actively catalyze CO oxidation. In the presence of Sn XRD analysis shows formation of a mixed phase CeSnOx and stabilization of more dispersed species with a fluorite-type structure, the factors significantly contributing to the high activity and thermal stability of the catalyst modified by Sn.

XPS analysis indicates the presence of both Pt^2+^ and Pt^4+^ cationic states in the catalyst Pt/CeO_x_, whereas only the state Pt^2+^ could be detected in the catalyst Pt/CeSnO_x_. Insertion of Sn ions in the fluorite lattice of Pt/CeO_x_ does not stabilize Pt^4+^ state in the Pt/CeSnO_x_ catalyst, but induces formation of remarkably high concentration (50%) of lattice Ce^3+^ ions. Results of our DFT calculations corroborate destabilization of Pt^4+^ ions by incorporation of Sn cations into the fluorite structures of Pt/CeO_x_ nanoparticles.

Our TPR-CO experiments show the presence of loosely bound active oxygen in the Pt/CeO_x_ and Pt/CeSnO_x_ catalysts in contrast with the similarly prepared pristine CeO_x_ catalyst. CO_2_ evolution in the gas phase upon interaction of the Pt/CeO_x_ and Pt/CeSnO_x_ catalysts with CO takes place in the temperature range 0–200°C, while no reaction of the CeO_x_ catalyst with CO could be detected at these temperatures. The TPR-CO results that the ignition temperature of CO oxidation over the Pt/CeSnO_x_ catalyst is lower than that over the Sn-free Pt/CeO_x_ catalyst strongly suggest the presence of more reactive oxygen species in the Sn-modified catalyst. Moreover, incorporation of Sn causes increased amounts of the loosely bound highly reactive oxygen (enhanced oxygen storage capacity). According to our DFT calculations, doping Sn cations are somewhat more energetically stable inside ceria nanoparticles than on their surface. The calculations also provide an atomic-level explanation for the observed low-temperature generation of surprisingly abundant reactive oxygen and reduced Ce^3+^ species in the presence of Sn in ceria, which is related to strongly distorted Sn-O coordination sphere compared to that of Ce-O.

Along with the effect of Sn incorporation in the fluorite lattice of CeO_2_ we also document interactions of tin and cerium oxides at the nanoscale, which stabilize highly dispersed cerium oxide species crucial for the observed enhanced stability of the modified by tin Pt/CeSnO_x_ catalysts.

## Data Availability

The data that support the findings of this study are included in this published article and the Supplementary Material. Extra data are available from the corresponding authors upon reasonable request.

## Author Contributions

TK, ED, ES, AS, VM, AZ, and SN performed experiments. KN performed DFT modeling, analyzed the calculated data, and was involved in the preparation of the manuscript. TK, ED, ES, and AS were involved in the analysis of the experimental data and the preparation of the manuscript. AB supervised the experimental work and was involved in the analysis of the experimental data and the preparation of the manuscript.

### Conflict of Interest Statement

The authors declare that the research was conducted in the absence of any commercial or financial relationships that could be construed as a potential conflict of interest.
