# Supplementary material for: Pt/CeO2 and Pt/CeSnOx Catalysts for Low-Temperature CO Oxidation Prepared by Plasma-Arc Technique
Source: Front Chem. 2019 Mar 12;7:114. doi: 10.3389/fchem.2019.00114 (PMC6424011; doi:10.3389/fchem.2019.00114)
Supplement: Supplementary file 1 [file Data_Sheet_1.pdf]

Supplementary Material

Pt/CeO_2 and Pt/$CeSnO_x$ Catalysts for Low-Temperature CO Oxidation Prepared by Plasma-Arc Technique

Tatyana Yu. Kardash[1,2,*], Elizaveta A. Derevyannikova[1], Elena M. Slavinskaya[1,2], Andrey I. Stadnichenko[1,2], Vasiliy A. Maltsev[3], Alexey V. Zaikovskii[3], Sergey A. Novopashin[3], Andrei I. Boronin[1,2,*] and Konstantin M. Neyman[4,5,6*]

1 Boreskov Institute of Catalysis, Siberian Branch of the Russian Academy of Sciences, Novosibirsk, Russia,
2 Novosibirsk State University, Novosibirsk, Russia,
3 Kutateladze Institute of Thermophysics, Siberian Branch of the Russian Academy of Sciences, Novosibirsk, Russia,
4 Departament de Ciència dels Materials i Química Física, Universitat de Barcelona, Barcelona, Spain,
5 Institut de Química Teòrica i Computacional, Universitat de Barcelona, Barcelona, Spain,
6 ICREA (Institució Catalana de Recerca i Estudis Avançats), Barcelona, Spain

*** Correspondence:** T. Yu. Kardash kardash@catalysis.ru
K. M. Neyman konstantin.neyman@icrea.cat
A. I. Boronin boronin@catalysis.ru

1 Supplementary Figures

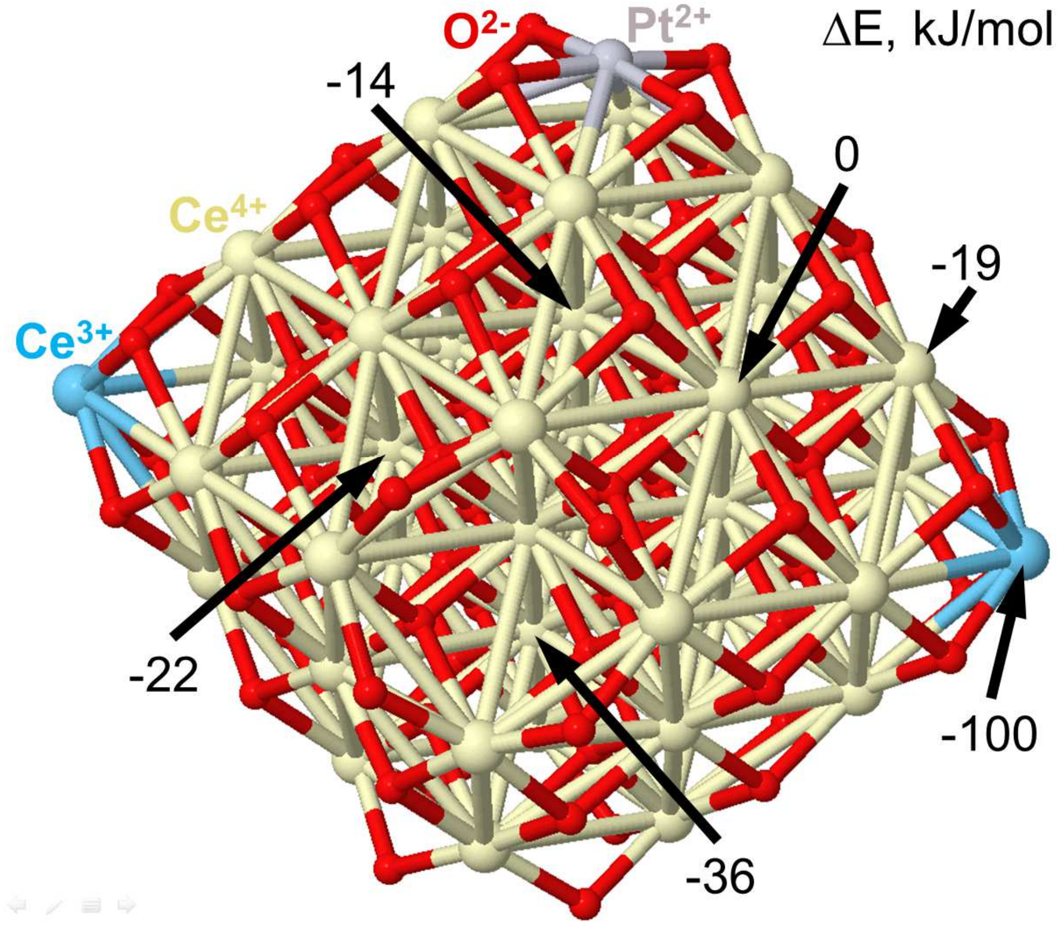

Supplementary Figure 1. Calculated by DFT relative energies ΔE (kJ/mol) of model nanoparticles $Pt/SnCe_{39}O_{80}$ with one doping Sn ion substituting one Ce ion in the indicated by arrows positions of the displayed pristine nanoparticle $Pt/Ce_{40}O_{80}$. Negative energy values correspond to more stable systems. Atomic coordinates and total energies of the fully optimized structures referred to in the figure are given below, in the Supplementary Data.

2 Supplementary Data

2.1 Cartesian coordinates (Å) and total energies (eV) of ceria-based nanoparticles obtained from DFT calculations after optimization of all atomic positions

$Ce_{40}O_{80}$ E = -952.28033 structure displayed in Figure 4

Ce 8.36569554839131 9.53799061874590 9.50494704478155
Ce 13.51212640006311 9.44923140477911 14.96185160718299
Ce 8.45413226660510 9.51940129431679 14.96021896022538
Ce 10.95263771352866 6.86548036115275 14.89040017176273
Ce 11.01672683992810 12.10158037019540 14.91001647558100
Ce 8.35463523199783 12.30604066649994 12.28537573431356
Ce 8.28649663911972 6.75577088609212 12.27248432696171
Ce 13.61576974195128 6.67353328461472 12.27268665387716
Ce 13.68927477213212 12.23499547316965 12.28449614220827
Ce 16.14602539068300 9.41715471375996 12.26255093025505
Ce 5.82371971466317 9.56677624783274 12.25819543998115
Ce 10.98837089973162 9.49582604099624 12.16260405903517
Ce 11.05847568905712 14.88871634413386 12.13178948780736
Ce 10.91131486302602 4.08565940855494 12.10720549761994
Ce 13.58723996327807 14.92343764792648 9.50865151450794
Ce 8.53852952380610 14.99938031833538 9.50820086350325
Ce 5.86679848087522 12.33698729953903 9.51320185645426
Ce 16.18745691010009 12.17535543478975 9.51199071701475
Ce 11.02281495327024 12.15920920514163 9.50878022690710
Ce 13.60751065563300 9.45511234490374 9.50563608770088
Ce 18.53649826735485 9.39036799109360 9.51706618716221
Ce 10.95177201256781 6.83953921689466 9.49816884776247
Ce 16.11716435880195 6.66774117632225 9.50207661418914
Ce 3.44647599161636 9.61607785183912 9.50498858253609
Ce 5.78967083881853 6.82089460427596 9.49756422186459
Ce 13.44114658957817 3.99929194671496 9.49214788353560
Ce 8.38321825860143 4.07876003790499 9.48704519278725
Ce 11.06455242249927 14.89505169918083 6.88659384573399
Ce 10.91265903068409 4.11747197657243 6.87105325351905
Ce 10.99260751758573 9.50612039333922 6.84742501757429
Ce 5.82941808930856 9.59010699473250 6.74810767219740
Ce 16.15602964575523 9.42641105913673 6.75475439146673
Ce 13.69910806910398 12.23969719953103 6.73537251748697
Ce 8.36093627806857 12.32103339344717 6.73146522980738
Ce 13.62134009197650 6.68619672700674 6.72453625562530
Ce 8.28364046739643 6.77324275535418 6.71829692464506
Ce 11.03201537614741 12.12634370138489 4.11038614238961
Ce 10.95837673990196 6.89597943632681 4.09879726213982
Ce 8.46862731188394 9.54794366517097 4.04023518021291
Ce 13.52452619879776 9.47250720178460 4.04688690482363
O 12.53702473380075 7.86567797103607 16.01580097527092

O	12.58095749018621	11.06113876398636	16.02336804004466
O	9.38796207979192	7.90545360977931	16.01044780892796
O	9.42955791904868	11.09985254578753	16.02528612735761
O	6.90717472763897	10.93148603011077	13.57347967837289
O	9.63712707222847	13.57505113236581	13.57863227315380
O	12.43448223439350	13.53703295242972	13.57692834936298
O	15.09848389256146	10.81471099059396	13.57389560755286
O	15.06211750064365	8.04144455890423	13.56810226442367
O	9.53375940146923	5.43904652662363	13.55873042608525
O	12.32975729206299	5.40098199158265	13.56136280609778
O	6.87425681532942	8.16329965496031	13.56574095249926
O	9.64279052697462	8.17413761295995	13.48705882560088
O	12.29722250774954	8.13791172505197	13.48795135727376
O	12.32972896246407	10.80451589636482	13.49614411007315
O	9.67442916762654	10.83933289420301	13.49748432331850
O	12.64961728664717	15.98811478248216	11.10961523966596
O	9.50830158970740	16.03886177496996	11.10769600556066
O	12.47241580590907	2.94850098944967	11.08767511965591
O	9.31923621137355	2.99999212673858	11.08362972847987
O	6.83699569171301	5.49094379989124	10.87763517907177
O	6.94637749265706	13.62472759492441	10.90018832062931
O	15.13692668441818	13.49821927730627	10.89841017104431
O	4.33148193709516	10.97486838568947	10.88860462228197
O	17.64553318581776	8.02044274096191	10.88832291745813
O	15.02682933534584	5.36885944069004	10.88180504911500
O	17.68402022935632	10.77526010774619	10.89383534956562
O	7.00229419899098	10.90812998279099	10.86003956506071
O	4.29444298212596	8.22025870177181	10.88129840976285
O	9.60177682254284	8.15983045045526	10.85613448906304
O	12.33636580796382	8.11651286046181	10.85722184788599
O	12.37420452702891	10.83195189460500	10.86256878942990
O	15.00857948706763	10.78723968208311	10.86071881913015
O	9.71218518365439	13.50634511302655	10.84252773216538
O	14.97304194702264	8.08113639952852	10.85373847770197
O	12.36775318479297	13.46724183279094	10.84169422672201
O	9.60623235502492	5.52253075310283	10.82446706820922
O	12.25776325889923	5.47985960550991	10.82644198270315
O	9.63738523154143	10.87458633216387	10.86469875962383
O	6.96750240503981	8.20163407751234	10.85323523821099
O	9.63931973603864	10.88117702887451	8.14885828859853
O	6.96613137623138	8.21178444940793	8.14679684563486
O	12.37337319265610	13.46962993264166	8.17468295331856
O	9.71942272001798	13.51187218736405	8.17512095311360
O	9.60727016462518	5.53451972141847	8.15840455213132
O	15.01174526256819	10.79204701736016	8.15521241386554
O	12.26349250835245	5.49365556164403	8.16183747405211
O	12.37664360513152	10.83623939222995	8.15230031019692

O 14.97631168216284 8.08672912702902 8.15059011229457
O 9.60301505814314 8.16789792451045 8.14421845408134
O 12.33797957647651 8.12328998552606 8.14422450565455
O 7.00718318185921 10.91771418612081 8.15412617173839
O 17.68679609173456 10.78043837453553 8.13884921663944
O 15.14342434642361 13.50350871372740 8.12587292433503
O 4.29419910651368 8.23099197654197 8.12466049594913
O 6.95185405386824 13.63282444105520 8.12836760855606
O 17.65110530420116 8.02545897173080 8.13486776449555
O 4.33459672876877 10.98692157818406 8.13183681636179
O 15.02971048397496 5.37518106908752 8.11188703696326
O 6.83895017830283 5.50109054840302 8.10834023486208
O 9.50711757524100 16.04014261619386 7.91244217697123
O 12.65551247671366 15.99431602815817 7.91327924532840
O 12.46723038717486 2.96521872545293 7.89534069894928
O 9.31839659878125 3.01368636103436 7.88703965736083
O 9.68394313321257 10.86140934928914 5.51409985370896
O 12.34167928432917 10.82026506884191 5.51867913118315
O 12.30212696809762 8.15536267715616 5.51096862498285
O 9.64706687401969 8.19714580565212 5.50758685618798
O 6.87629860020124 8.18756929383626 5.43425246210629
O 15.11205634798631 10.83058762736560 5.44431312635447
O 12.44411325598043 13.55179507706054 5.44231429982519
O 15.07059365741251 8.06038495646819 5.43768543888598
O 9.64695430632066 13.59633799584228 5.44106082330687
O 9.53366674955676 5.46601149138575 5.42386502835078
O 12.33127456207715 5.41984717669171 5.42831567818065
O 6.91535179105843 10.95869376402726 5.43880520588171
O 9.44586355709127 11.12844902757492 2.98394942924708
O 12.59304958441505 11.08726447385377 2.99294706272251
O 9.40269346544429 7.94110787709011 2.97839415646301
O 12.55115537208184 7.89451046274540 2.98225695240772

$Sn^{4+}(Ce^{4+})_{39}O_{80}$ E = -945.24521 structure displayed in Figure 4

Ce 13.50299153668393 9.45094285285288 14.95268138394033
Ce 8.46137054086593 9.51368414709677 14.94756034116818
Ce 10.95903504135616 6.85787308914738 14.88035430903443
Ce 11.01799100378334 12.10417472528720 14.90204648356128
Ce 8.35507778437553 12.29003753971962 12.27302910306163
Ce 8.29162966495310 6.76623639894322 12.25816051829828
Ce 13.61222505645856 6.67427336020105 12.27145214388186
Ce 13.68504466734232 12.23447571388973 12.28322476517216
Ce 16.13962412237184 9.42120085996463 12.26336806062655
Ce 5.85418329151844 9.56285786637655 12.22169316138053
Ce 10.97116252377516 9.49345297875245 12.14362721587280
Ce 11.05758903903639 14.87747549436926 12.13954094262740

Ce 10.91129059754215 4.09117743067965 12.10987639335405
Ce 13.57627907308284 14.92053411766600 9.50875680149605
Ce 8.54076366910813 14.98859239586707 9.51146329454839
Ce 5.89354093310215 12.29861500652532 9.51305760409473
Ce 16.17593893442881 12.17948647698076 9.51322182184377
Ce 11.00677776226747 12.14392190174139 9.50924291269131
Ce 13.59805523059103 9.45622260886450 9.50712946505190
Ce 18.52884128735182 9.39440428242024 9.51810856632070
Ce 10.93867365698936 6.85438079836850 9.49583752713326
Ce 16.11106291295896 6.67030946210771 9.50382002628050
Ce 3.45977320826415 9.61301360958576 9.50507280689949
Ce 5.82214051109744 6.85815084921642 9.49484037781328
Ce 13.43301457175414 4.00778704750534 9.49277234526921
Ce 8.38344278726811 4.09172886119015 9.48581043190133
Ce 11.06087098824260 14.88767117949932 6.88006350341218
Ce 10.91389433687576 4.12127132401261 6.87020254372969
Ce 10.97645603621882 9.50789302983174 6.86348276037582
Ce 5.85763034108429 9.58670021844278 6.78150499476129
Ce 16.14998740615536 9.43053774098860 6.75791338084135
Ce 13.69316331236928 12.24162959083274 6.73566155387098
Ce 8.35734031545666 12.30705689839303 6.74685478297018
Ce 13.61911950790368 6.68916098456293 6.72459180225320
Ce 8.28925547310841 6.78780984867908 6.73091282102217
Ce 11.03186507813878 12.13314472133228 4.12004606380450
Ce 10.96056500401538 6.89063763078176 4.10817849017750
Ce 8.47047742196012 9.54797023239214 4.05303260529418
Ce 13.51468419976081 9.47563562208365 4.05410377593449
O 12.53235822297906 7.86256287250116 16.01044232160241
O 12.57511549959570 11.06128225284128 16.01912363859635
O 9.38222736436977 7.90759636385233 15.99553903908745
O 9.42356586713646 11.09465551604300 16.00989470880040
O 6.91180370180226 10.93777671109399 13.54151058924992
O 9.64990024134848 13.57173355068030 13.57950935576942
O 12.43990081042964 13.53915962129016 13.58004407403536
O 15.09438640451672 10.82003020139897 13.57258357545724
O 15.05864505528158 8.04819854625280 13.56678295387016
O 9.55362510807882 5.43675230531018 13.55659366336089
O 12.34161282582439 5.39701479432029 13.56470622614980
O 6.88151344404485 8.14779681290645 13.53292252249315
O 9.64949275611806 8.16674814615044 13.46699168429750
O 12.29391267546035 8.13620584373329 13.48141094419896
O 12.32555245393117 10.80415357533095 13.48960311360468
O 9.67952030793696 10.84047876287433 13.47668336853708
O 12.63627104600154 15.98540123862210 11.11058292472398
O 9.49395676703920 16.02245664041602 11.10959727859226
O 12.46669990894568 2.95245900717697 11.08786969911258
O 9.31030007688790 3.01272998092754 11.07806133462908

O	6.84288404375577	5.52002529253493	10.88406243365952
O	6.94914879352077	13.59165142131667	10.91153806746676
O	15.13100280375881	13.50196278119648	10.89907275120508
O	4.35955294070976	10.96598187460945	10.88521358546767
O	17.63917409725459	8.02248515139141	10.88847899683284
O	15.02159194151786	5.37182700272607	10.88284487248891
O	17.67240853805398	10.77865328497307	10.89552676058966
O	7.06671303655572	10.84721006961932	10.79852311688221
O	4.32433753070752	8.21957176878987	10.87530890132537
O	9.55331420179059	8.20836758261033	10.80100573888363
O	12.31978558616348	8.11731049612319	10.85641728265280
O	12.35382986392605	10.83213374432895	10.86209155660147
O	14.99361797213440	10.78971837028580	10.86043521777422
O	9.71612331840590	13.48600453198637	10.84761473525102
O	14.96203689387178	8.08528054358541	10.85349314719151
O	12.36284963536942	13.46208424444379	10.84206976547532
O	9.61097289973033	5.53934251506265	10.82738292375342
O	12.25384498045570	5.48552326780679	10.82751935322791
O	9.58456647768442	10.82103194677481	10.81084876294687
O	7.03871395450758	8.26041000358514	10.79294664710750
O	9.58301568986060	10.82673960367213	8.20061258812025
O	7.03850380125804	8.27356921001096	8.20527849676158
O	12.36666952582092	13.46580550841831	8.17243611588484
O	9.72070447176231	13.49183456102481	8.17145549892978
O	9.61191166260011	5.55203071703589	8.15522501687476
O	14.99742136005855	10.79442470265985	8.15882893022622
O	12.26054328338202	5.49740947638957	8.16113256471412
O	12.35595349247432	10.83688082102895	8.15303809277087
O	14.96566293574577	8.09157772496172	8.15384862863922
O	9.55363451049754	8.21634681899040	8.19124424236189
O	12.32118594516573	8.12446458086202	8.14468235689527
O	7.06661046450828	10.85999874770693	8.20979963794092
O	17.67570849137642	10.78423275329848	8.14099927104623
O	15.13601934805700	13.50701356119969	8.12763182282812
O	4.32571200570950	8.23031569325335	8.12867962734064
O	6.95477172417787	13.60265262031862	8.12033330797119
O	17.64443046396679	8.02987917666598	8.13624715704043
O	4.35889804582869	10.97772388016828	8.13640700902561
O	15.02890073037498	5.37786463300610	8.11385941871010
O	6.84282938248765	5.53381682286004	8.09330464609038
O	9.49668848253094	16.02645611145773	7.91719685949200
O	12.64449844187375	15.99152555894529	7.91208142885329
O	12.45609194546181	2.96590727855499	7.89872820076200
O	9.30684092684656	3.02742535079255	7.89187747515677
O	9.68641314188925	10.86670246689752	5.53492602301870
O	12.33559819835708	10.82285685200668	5.52389719551204
O	12.29901063353202	8.15640994235601	5.51737193874422

O 9.65192994893146 8.19471074502600 5.52538899981578
O 6.88210203186300 8.17607271147024 5.46388226353905
O 15.10701954179365 10.83536854316985 5.44732049757209
O 12.45099231287811 13.55629435117805 5.43676072786114
O 15.06968084545121 8.06538857057919 5.44089069531318
O 9.65908764049788 13.59563611022328 5.44234974455161
O 9.55472630896974 5.46581684987514 5.42521078318058
O 12.34513176334373 5.41809701160019 5.42689485263989
O 6.91551781169927 10.96636792129797 5.46766306967704
O 9.43541113234018 11.12630598511548 3.00125652732116
O 12.58422821966532 11.08874277563465 2.99675866995108
O 9.39350129829161 7.94419839517179 2.99518500412643
O 12.54519754323166 7.89379382554134 2.98806084695934
Sn 8.39988544346072 9.53606349494848 9.50162825105875

$Pt^{2+}/Sn^{4+}(Ce^{3+})_2(Ce^{4+})_{37}O_{80}$ E = -952.94182 structure displayed in Figure 4

Ce^{3+} 18.68466023492496 9.34090354724752 9.50831216892362
Ce^{3+} 3.30218908234318 9.58804593569100 9.50206973986196
Ce 13.52681968928045 9.41640706790851 14.93552148889649
Ce 8.43705065985603 9.49662188422508 14.92372690015727
Ce 10.94800185249316 6.85213987823442 14.85997468201140
Ce 11.02682420414929 12.06409731837135 14.94206049369527
Ce 8.32680036899023 12.24169199155660 12.27798710508086
Ce 8.26280234151752 6.77416311821923 12.23854771129220
Ce 13.62202977096607 6.67182501719867 12.26194125108863
Ce 13.73476079960895 12.16398349274827 12.29986507355776
Ce 16.19242981640897 9.37358603159743 12.20466381516863
Ce 5.80226558645610 9.54331441739548 12.17668340151340
Ce 10.96939385510633 9.49191368242476 12.14294236895561
Ce 11.06703100889319 14.76013017075091 12.22293895387981
Ce 10.90204751773863 4.10550425882556 12.09870541164022
Ce 13.70825968492232 14.74847274557495 9.52358320878323
Ce 8.42279570573923 14.82699981538824 9.51734569698831
Ce 5.80895131741276 12.20069986805606 9.51301537159187
Ce 16.27742504602471 12.06295469536414 9.51462691139433
Ce 11.01175928207706 12.14282035728140 9.51453651121203
Ce 13.59242273646869 9.44760255705063 9.50495146329684
Ce 10.93336803435485 6.86409020125570 9.49309685277574
Ce 16.14141594523988 6.69999540834170 9.49574513924010
Ce 5.77324684005880 6.89630692635552 9.48955725474883
Ce 13.44817756864743 4.03661495309290 9.49435645997524
Ce 8.36571562214847 4.12203355692365 9.47700086404296
Ce 11.07401002069513 14.77415733027788 6.82045583199729
Ce 10.91336037598338 4.12465729022901 6.87327819652739
Ce 10.97702485788948 9.50809322737920 6.86555549276510

Ce 5.79794986458739 9.55761634947342 6.82682065188628
Ce 16.19903149034392 9.40120060834933 6.80546328845554
Ce 13.73937141389678 12.18435893537747 6.72842736717570
Ce 8.32874481605155 12.25248342406291 6.74801726527743
Ce 13.63072208568636 6.68697209788366 6.73004179805438
Ce 8.26640435226885 6.78916536584941 6.73919660000022
Ce 11.03510443233014 12.08888812310764 4.08387912473392
Ce 10.95075852801884 6.88827928492352 4.12877437894045
Ce 8.43813603583012 9.52650711785349 4.08314431563478
Ce 13.54187882586437 9.44330336353275 4.07509112014463
O 12.54421004878094 7.85728418204471 15.99754250387024
O 12.58181971370822 11.04958979844111 16.02344349076274
O 9.36752533241901 7.91292108353297 15.98292480886480
O 9.42553129268794 11.08987206252178 16.00904749729956
O 6.92566936410900 10.94511438119477 13.52763487326578
O 9.66259920138723 13.61626802681280 13.56954639166371
O 12.44172411843638 13.56918541852425 13.57886260955625
O 15.09042953409760 10.80913824341143 13.55516451964512
O 15.06272669863345 8.03552885340440 13.55566744651886
O 9.54640074163846 5.44992619107483 13.55458933061131
O 12.33021768490962 5.40437821860592 13.56624420968503
O 6.87349119491028 8.15616209022191 13.52752347236497
O 9.64179981961984 8.17758069555400 13.45899398742686
O 12.29078903813441 8.14068359782955 13.47601600234478
O 12.33382254779534 10.80545316540758 13.49525325377201
O 9.68378661590178 10.85394808833527 13.47790913849616
O 12.52589694844458 16.12663227809701 10.96510021430285
O 9.62829271549877 16.15652675953708 10.96464239471905
O 12.46747346337439 2.97524184870886 11.08698082348161
O 9.29982557533139 3.03333063710521 11.06712893500049
O 6.83958880410022 5.53999950400346 10.88423137028202
O 6.97339105703657 13.61154615201075 10.88842312426582
O 15.13035876273370 13.50253652886851 10.88871812109894
O 4.40687181024262 11.00519373971228 10.90969519707451
O 17.58648451818060 7.96619613585988 10.91687284837045
O 15.01169604334699 5.37827838695094 10.88077003024435
O 17.63501284367482 10.79769332302349 10.91326824863863
O 7.08150308131728 10.84707421923910 10.79569002258863
O 4.36801210627273 8.17957085529074 10.91375170027420
O 9.55714636017699 8.23092700755974 10.80030321279185
O 12.30799009459609 8.13312602365187 10.85583072760475
O 12.34902398575541 10.85180964091559 10.86370377874825
O 14.98787082519985 10.77365735471468 10.85357310251903
O 9.69772571556653 13.49702891738648 10.87575161557196
O 14.93557365297649 8.08633768908993 10.84186196261807
O 12.40246717014015 13.46852137382382 10.87968348590513
O 9.60294732467414 5.56008465429382 10.82180530197208

O 12.24662205865745 5.50441373100032 10.82607453992736
O 9.59600327763064 10.84358351625467 10.80767971932400
O 7.05073871026048 8.27593527501238 10.78036559317466
O 9.59724779326316 10.84952461694613 8.20864371953714
O 7.04776353965117 8.27888688216204 8.20848394431719
O 12.40410542553570 13.47533039095280 8.16054570438957
O 9.69877585190439 13.50314547733721 8.15926415125880
O 9.61030765003590 5.56871942089157 8.15051297184662
O 14.98928332256122 10.78445446864848 8.16586076716914
O 12.25763371300353 5.51369718110408 8.16105839173573
O 12.34978572114527 10.85920745122546 8.15569253924543
O 14.93671848713790 8.09631071128432 8.15669545488339
O 9.55632682446712 8.23741629919908 8.19178606474946
O 12.30963810510004 8.13966121927909 8.14414816060286
O 7.08044923847949 10.84812594505830 8.21853629047451
O 17.63531893810012 10.81451291647500 8.11168034337943
O 15.13157956683010 13.51268204045075 8.15039072426437
O 4.35968830414327 8.18481278069166 8.08011507642491
O 6.97390336066529 13.61465300277526 8.14196892645828
O 17.59032934067099 7.98286078197321 8.07698873865138
O 4.40978188899883 11.00952017250519 8.10881115598818
O 15.02141898953987 5.38493147021757 8.10568370586219
O 6.84423408016442 5.54301915374081 8.08970530721439
O 9.63057950766604 16.16393544162882 8.07672929106472
O 12.53030953104005 16.13349876378717 8.08293880270671
O 12.46454401405205 2.98486019852362 7.89475956555723
O 9.30068835141265 3.04329389540912 7.88641863339813
O 9.68718185832934 10.87562769131605 5.53931182496606
O 12.34346767589545 10.82751834715136 5.52296997695326
O 12.29653014568926 8.16208712810340 5.52446505114273
O 9.64717445846444 8.20366413298595 5.53573467260067
O 6.87420637288013 8.17741906981815 5.46627746917252
O 15.10262046185079 10.84237245885642 5.46308961618533
O 12.45372683637359 13.59230797494069 5.45905439213176
O 15.06816390797293 8.06725039880636 5.44092915280815
O 9.67343079440232 13.63302479404174 5.46661079404108
O 9.55068215582451 5.47631803086494 5.41919922779411
O 12.33520108871327 5.42742476906558 5.42118879656311
O 6.92495401970892 10.96470845447831 5.48580322557433
O 9.43441139759443 11.12162121173295 3.00943764347239
O 12.58648453800873 11.07689855229165 2.99639507406761
O 9.36604819379310 7.94779658392178 3.01278573856959
O 12.55379611559890 7.89228120134182 3.00243460440449
Pt 11.08048321646713 16.25399413760300 9.52252771059436
Sn 8.39312422179623 9.52922331656226 9.50069525976734

$Pt^{2+}/Sn^{4+}(Ce^{3+})_2(Ce^{4+})_{37}O_{80}$ E = -952.72119 data in Supplementary Figure 1

Ce^{3+} 18.70071702763756 9.34116038164475 9.49603607236091
Ce^{3+} 3.28804563846170 9.57571383635398 9.51233580306904
Ce 8.38069896298044 9.52955757858685 9.50815811102051
Ce 13.55105118154481 9.42327748498019 14.93864042953673
Ce 8.43806893965044 9.49677935452203 14.93433939379646
Ce 10.96064791605458 6.86321548163896 14.87174784569260
Ce 11.02428278752145 12.06183162922292 14.94939496674261
Ce 8.32782674131141 12.25248131143194 12.29567497267396
Ce 8.26806598489667 6.74836689132939 12.26223567626193
Ce 13.62847422943202 6.67230453599571 12.26128368640036
Ce 13.74321828248233 12.16469855495175 12.30561357705803
Ce 16.21110761290917 9.37674465258429 12.20693667078505
Ce 5.77988795264536 9.53856025766226 12.20653454618017
Ce 10.99244586370568 9.49068548874128 12.16014833920374
Ce 11.07250447270714 14.77291124873347 12.21520015807439
Ce 10.90717079386680 4.09027360099496 12.08838031144310
Ce 13.68197495533330 14.73157375429684 9.48306673032806
Ce 8.40914875282560 14.83873569231473 9.51853852054860
Ce 5.77628316543327 12.22472953187699 9.50741631177014
Ce 16.28256971687143 12.03011413220178 9.48663208558849
Ce 11.02773458289736 12.16866236805076 9.51531981380447
Ce 13.62071161372796 9.44017513397027 9.51469941497765
Ce 10.95448939563507 6.84855340347552 9.49433700784413
Ce 16.15443510463147 6.69284876559808 9.49165880877653
Ce 5.75400803047523 6.86151094457124 9.49388206895056
Ce 13.46390751006340 4.03355320214755 9.49123774620790
Ce 8.36558032044578 4.10729604012453 9.47173688049597
Ce 11.06922320691235 14.78396455895644 6.83805367785663
Ce 10.91441047144636 4.12342801838413 6.87317862694456
Ce 10.99843465539153 9.51107039861165 6.84040903845524
Ce 5.77403047379780 9.55628775536041 6.80529703343448
Ce 16.19742757410638 9.42981562757934 6.82939958253164
Ce 8.31896144208351 12.26666686909243 6.72391592580407
Ce 13.63980713656349 6.68685481517178 6.72372396623527
Ce 8.27253276946889 6.77861889875996 6.72658908858834
Ce 11.02783872139402 12.08002700679761 4.07272996266268
Ce 10.95038605815951 6.90502217041015 4.11127122758840
Ce 8.42551826681655 9.52462702418893 4.06636095083783
Ce 13.52235440398315 9.48653122436076 4.08334191506000
O 12.57291655677094 7.85556492555339 15.99818891227306
O 12.58321228067311 11.04602006114191 16.01981803341002
O 9.39761913645425 7.91907638177059 15.99986855849580
O 9.42840948955200 11.10021276398684 16.02289593884704
O 6.93109157700089 10.93439870325372 13.55649465256455
O 9.65922114463661 13.61505855187116 13.56810105464817

O	12.44744042039254	13.57483410634927	13.57557049314407
O	15.09911079609627	10.82096396226035	13.58515384776815
O	15.06933052769438	8.04131424311241	13.55209278952261
O	9.53358282819741	5.44620797914226	13.55717457855195
O	12.32507788472896	5.41214761348175	13.55837216733991
O	6.87168636499078	8.16331171674485	13.55894236422318
O	9.64239301513092	8.17910048554462	13.47707356452212
O	12.29969518704592	8.15018351971841	13.48673974146800
O	12.34784102059309	10.81637843145191	13.48403137779530
O	9.68685046003572	10.84678659197604	13.50149063188515
O	12.53369205559965	16.12070254206206	10.95061142884938
O	9.63974374596515	16.17221092813093	10.96911307209307
O	12.48261297205087	2.96781799515314	11.08689369450979
O	9.31186514124717	3.01443061284909	11.06587098502483
O	6.83741307250704	5.50344268408663	10.87408272059113
O	6.96818522624705	13.63282852769316	10.88116312555552
O	15.16211767691041	13.54504008295735	10.76554554274956
O	4.37969978145822	11.00634377055082	10.90962053008018
O	17.60229458286938	7.95703452783183	10.91926662335764
O	15.01184329659375	5.37909104479589	10.88126476279167
O	17.63074638194638	10.78036081287760	10.90217490165504
O	7.01912279095727	10.90020015554223	10.85270225866407
O	4.34079561282228	8.17484953370061	10.92556969503648
O	9.61609564487799	8.17447960905610	10.85331426000140
O	12.33317129577338	8.11734104901847	10.87145267375838
O	12.35445692134919	10.83893816487987	10.83020188689393
O	14.98726251721665	10.79992478942458	10.91717455653928
O	9.69677870588506	13.52129527703140	10.87079589793581
O	14.96165684640867	8.09046383521994	10.83620510578963
O	12.41600013646046	13.46554038292379	10.89102594864897
O	9.60175263794607	5.53790694533607	10.81783317557652
O	12.25247656003306	5.49118455282434	10.81845639851154
O	9.64310459480968	10.89784637928719	10.87263372471554
O	6.99415441004323	8.20997210727622	10.84468340870750
O	9.70148472710573	10.89191786854735	8.16734035586028
O	6.98720089740399	8.22314202343959	8.15767738505267
O	12.31727585394096	13.50768603164606	8.20184527067096
O	9.65349789384396	13.52239683674586	8.15179535811833
O	9.61454993567322	5.54719442778434	8.15517959973417
O	15.11209742758253	10.63018277800650	8.31505376175000
O	12.27409886217536	5.52754091988949	8.15090870651759
O	12.50372358166393	10.94437479433800	8.07251519371595
O	14.93215564082854	8.03480989075546	8.12744555774504
O	9.60456535407384	8.17714394529122	8.13837631995870
O	12.31220540747514	8.17631571386881	8.15970177781068
O	7.04321058695355	10.91145558757531	8.15744383028801
O	17.70612004166029	10.83975735045018	8.08853955042130

O 14.92676088307960 13.29085121128123 7.99067843269527
O 4.33477526204546 8.18028181516783 8.08177871376581
O 6.94616931424305 13.63830643393688 8.13728939566095
O 17.58557976616766 7.99365120130612 8.08378168977389
O 4.39085980500167 11.00770706159213 8.10995732902228
O 15.03195710263386 5.35756428405507 8.10380761009310
O 6.84776334837378 5.51664818187377 8.09855513481019
O 9.62755727558294 16.18088433856387 8.07893928390574
O 12.52342898202603 16.15094087230434 8.06680011878732
O 12.46489133882533 2.99030376412552 7.89536516353903
O 9.30641763550778 3.03210820114368 7.86941322116759
O 9.64430107466719 10.87719614016325 5.52361677566291
O 12.28069627027475 10.81372281694822 5.49151698132182
O 12.31073110233964 8.15351267741449 5.52894698434788
O 9.65092441449560 8.21659225639522 5.51130381283281
O 6.87229264759145 8.18992214423770 5.44368624224057
O 14.94526337277393 10.99159411323360 5.66939646749018
O 12.55259800643353 13.49737400461034 5.57790731063312
O 15.08712888450631 8.17728967242489 5.39873146499873
O 9.72083999757836 13.64272857048698 5.44055675342052
O 9.54325379695387 5.48494892953978 5.41844864457582
O 12.34416396323977 5.43228539789715 5.41541596306641
O 6.90224132999125 10.97162885411310 5.46156967457810
O 9.44352755310653 11.10802600311517 2.98948032420876
O 12.59419148498698 11.10241028179430 2.97328186256703
O 9.35305466358984 7.93463978198815 2.99780679236656
O 12.52223782153979 7.94136387977408 2.99937177669109
Pt 11.08051572327534 16.26622910723609 9.51944209923670
Sn 13.62948748310895 12.17081386195032 6.75070927881018

$Pt^{2+}/Sn^{4+}(Ce^{3+})_2(Ce^{4+})_{37}O_{80}$ E = -952.86120 data in Supplementary Figure 1

Ce^{3+} 18.68478386965996 9.35264798923768 9.49734917760555
Ce^{3+} 3.29019103056706 9.59138695626262 9.50917216255855
Ce 8.39245184094312 9.55007958815671 9.50432707161045
Ce 13.54545127358638 9.42134090039699 14.93138366254504
Ce 8.42767478363085 9.49470064234559 14.92801342565335
Ce 10.95360588142459 6.86927266869774 14.86676215807025
Ce 11.02249398469929 12.05699384373106 14.93712962539859
Ce 8.33923656533412 12.24986798554434 12.27451663138500
Ce 8.26655666745426 6.75886806505324 12.26174366073754
Ce 13.62712720729133 6.67398736167230 12.26564699272678
Ce 13.72240348635323 12.16288412123831 12.27803823347738
Ce 16.19600891931202 9.37229619235575 12.20686125122529
Ce 5.78132659962866 9.54119239412065 12.20945247763404
Ce 10.98976829690127 9.50681127912673 12.14658483301200
Ce 11.06843633075964 14.74328847873861 12.18272856874542

Ce 10.90198633690219 4.10138118971330 12.09453470168050
Ce 13.69185415797770 14.72370239662651 9.51943963226707
Ce 8.44760844210107 14.80577451769259 9.51327601488008
Ce 5.78475128400313 12.22346452737205 9.51009330149580
Ce 16.27243714013440 12.05936497066248 9.51753903003036
Ce 13.58588118087913 9.46548180893443 9.50691166281681
Ce 10.95083374799896 6.85554727234360 9.49586523751786
Ce 16.14124926646658 6.70857926394113 9.49773398089892
Ce 5.75856552841171 6.87330865800847 9.49650356130634
Ce 13.45580871500822 4.03790310016053 9.49353186317049
Ce 8.36337063450514 4.11780770833157 9.47775684529066
Ce 11.06872672131794 14.75705270496350 6.84918484047457
Ce 10.91245805382608 4.12533490571821 6.87447412020720
Ce 10.99075678128755 9.52106830081665 6.86602779104587
Ce 5.77524465625613 9.55444372709933 6.80190746828917
Ce 16.19600848055759 9.40106804386863 6.80163005365502
Ce 13.72454804481470 12.18058181479965 6.74798004463495
Ce 8.33714285179084 12.26442183378224 6.74784092123993
Ce 13.63447772998834 6.69208161729250 6.72845313454239
Ce 8.26253546693575 6.77662600910392 6.72402082912005
Ce 11.02540408460234 12.08105015933959 4.08959020284226
Ce 10.94929698124592 6.89990373748947 4.12355213122583
Ce 8.42925726345591 9.52368647918376 4.08325004090062
Ce 13.54899538380796 9.44377677734605 4.07596741308463
O 12.55768988837037 7.87085669404749 15.99884127635712
O 12.58268123979297 11.05976467850874 16.00987163584972
O 9.37587637221788 7.91348142984743 15.99436928296877
O 9.43248172556723 11.10073655854729 16.00891813878833
O 6.92508469653231 10.92280346957524 13.55493765018895
O 9.64230264350611 13.61289886146742 13.53671230515626
O 12.44255430503811 13.56641938488801 13.53827145926552
O 15.09163998461479 10.79868536047373 13.55460285297609
O 15.06807863885059 8.02831312851088 13.56056077529684
O 9.53088909478875 5.45159554692145 13.55794224148434
O 12.31999539409621 5.41089275850499 13.56343073549109
O 6.86942124097109 8.15638698544051 13.56151955994084
O 9.64206416154754 8.18542329159216 13.47589064265107
O 12.29870551838098 8.14734794257728 13.47921202313561
O 12.34195398468570 10.80340859323799 13.48070574955757
O 9.67522944505493 10.84457889001043 13.47854186235098
O 12.54284745673279 16.09774934079826 10.96682212340721
O 9.63856647324123 16.14356765285520 10.96574571866622
O 12.47386684419780 2.97872526516722 11.08600623349560
O 9.30843064483521 3.02554754517124 11.07075816003590
O 6.83758744685151 5.51078169872023 10.87651435999406
O 6.99403813234667 13.62556838289153 10.88414502793952
O 15.10857428312337 13.49724382181325 10.89268396035883

O	4.39169675762816	11.02146157274329	10.90585508572871
O	17.58999917960618	7.97350198996423	10.91795029984813
O	15.01332993001446	5.38078328241724	10.88220604411740
O	17.62900271933950	10.80791176317049	10.90710764496713
O	7.03901201971377	10.89905764831127	10.85574158260529
O	4.33828292816950	8.19009915490478	10.92392190228172
O	9.61005548931494	8.19052215492865	10.85643691548436
O	12.32689943232128	8.14785847451662	10.85866852455024
O	12.31531143900748	10.90160790955652	10.81049575940435
O	14.98035781395704	10.77392438633952	10.85753250840412
O	9.75587310265630	13.45247327577661	10.80719089912324
O	14.94172692752326	8.08982918948677	10.84541921702438
O	12.34359743653237	13.41226930533576	10.80963826236093
O	9.60260962225118	5.54911549746547	10.81922355422932
O	12.25187959310163	5.50925007827446	10.82508355553605
O	9.70797483249998	10.94225603537780	10.80929702813484
O	6.99363102271606	8.21692042978804	10.84589215506051
O	9.70783624299862	10.95169168638372	8.20636629800449
O	6.99112797005682	8.22568572589573	8.15437615031457
O	12.34260346351904	13.41996651691768	8.21854880996993
O	9.75550939322184	13.46080755890724	8.21576753902712
O	9.60789471834959	5.56093393601165	8.15600054213627
O	14.98084200345818	10.78375558608270	8.16384482965186
O	12.26087203845446	5.52041812801080	8.16181385763437
O	12.31590807786302	10.90949057203664	8.20853181191260
O	14.94536200587125	8.10122320807872	8.15494065082207
O	9.60933985114866	8.19936620795639	8.14329155782308
O	12.32901740496786	8.15738901541980	8.14325716870728
O	7.03816621575235	10.90638492350797	8.16035964656920
O	17.63061229640694	10.82396506450272	8.11567311184683
O	15.11013448820456	13.50569920278473	8.14653934113355
O	4.33305352031266	8.19581851402937	8.08077658399708
O	6.99576302622928	13.63428413490634	8.13860798203790
O	17.59808272109637	7.98904906702404	8.07498039705277
O	4.39181660654575	11.02723400575568	8.11351410784388
O	15.02169539725227	5.39027504492382	8.10592984203194
O	6.84084938419215	5.51908889512669	8.10286090208153
O	9.64011675774262	16.15277493546472	8.07187851056531
O	12.54454477926755	16.10482030279239	8.07519906055728
O	12.47274705037879	2.99128968229733	7.89496721907394
O	9.30898433408735	3.04042586281972	7.87941516313630
O	9.67563906482672	10.86451829127523	5.53798022126243
O	12.34798399492691	10.82328774097979	5.53884513093473
O	12.29745717720430	8.16616555351602	5.52173124846352
O	9.64090396344027	8.20926006335574	5.52366406060120
O	6.86490062873717	8.17576541690854	5.43920051313070
O	15.10187671289489	10.82979571253034	5.46434116881185

O 12.44619332436785 13.58724309072878 5.49042392225398
O 15.06758305671480 8.05870716631971 5.43704792400366
O 9.64607753511476 13.62971061615086 5.48859555505862
O 9.53254518611496 5.47915931445650 5.41692843224971
O 12.32474369044180 5.43259897241129 5.42347098676904
O 6.92219337840568 10.94255320782997 5.46292875944303
O 9.43978983998813 11.13054118457817 3.00893601061551
O 12.58433168083097 11.08321030189444 3.00952709492545
O 9.37447076026404 7.94985805888826 3.00269141723650
O 12.56270160918604 7.89722375838637 2.99995937354897
Pt 11.09317802917755 16.25563936212735 9.52006961664779
Sn 11.03033561503099 12.13775498938895 9.51127801089358

$Pt^{2+}/Sn^{4+}(Ce^{3+})_2(Ce^{4+})_{37}O_{80}$ E = -952.92258 data in Supplementary Figure 1

Ce^{3+} 18.63813617292215 9.57104011700463 9.50664361264846
Ce^{3+} 3.28504176054263 9.59643001901295 9.51553421902251
Ce 8.36887687831796 9.53019236941942 9.50935274874302
Ce 13.52732468515016 9.41031021185895 14.94668537006604
Ce 8.42785560142817 9.49311973040115 14.93733094267777
Ce 10.94351880410970 6.84869698823127 14.86698301749618
Ce 11.02082161086917 12.05642729440466 14.95125376980748
Ce 8.33074589457154 12.25528384155977 12.29674600654508
Ce 8.25854525047762 6.74608949863565 12.25788247801561
Ce 13.62981727632478 6.66943532066091 12.26771042876920
Ce 13.74218113724141 12.14533498784978 12.29737043132049
Ce 16.18484956985078 9.40327110173757 12.21268412562516
Ce 5.77333399760211 9.54363498839301 12.21215451172949
Ce 10.97838911585411 9.48356107504453 12.16356220953221
Ce 11.10514284988122 14.75995380420846 12.21075087915877
Ce 10.90967412364537 4.08321122655864 12.08773204165622
Ce 13.77663832632081 14.71742611450470 9.51579516087041
Ce 8.46023923255516 14.83858245472988 9.51626271630794
Ce 5.78382116074059 12.23332877102674 9.51378863573942
Ce 11.03879761903829 12.15245396215097 9.51103909533068
Ce 13.57933285534114 9.42540006053902 9.50424100830082
Ce 10.94578384060775 6.83351799938102 9.49449601438209
Ce 16.14888918902917 6.72959323404845 9.49713153879057
Ce 5.74057287339428 6.86578952772445 9.49566663246346
Ce 13.46963743109965 4.02147301842022 9.49126803083199
Ce 8.36159300460452 4.10121223346922 9.47664287931922
Ce 11.10560848203943 14.77668588501634 6.81703044907834
Ce 10.91412923820582 4.12053122906454 6.87501577425711
Ce 10.97933306835878 9.49625144684513 6.84612095534146
Ce 5.76790459842899 9.55831873876237 6.80522285456107
Ce 16.18286365704869 9.42099271223382 6.79907047034406

Ce	13.74196139939663	12.15800675771964	6.72770529031151
Ce	8.33234381369944	12.26594131738672	6.73344706437816
Ce	13.63357162065995	6.68286691566946	6.72307562184061
Ce	8.25233967373654	6.77140236254319	6.72792091059981
Ce	11.02562145891489	12.08011640599572	4.07428480900228
Ce	10.93012257212095	6.88337040352269	4.11707263350097
Ce	8.42446619543221	9.52596086163976	4.07090908392334
Ce	13.53240112052094	9.43191021061149	4.06088674966841
O	12.54729559574621	7.84127281999957	16.00140843307518
O	12.57762337622758	11.03625685553335	16.03205286851248
O	9.37375083021935	7.90566915565073	15.99588236809925
O	9.42748360334589	11.09402555629198	16.02422277431446
O	6.92505622452135	10.93521147861759	13.55886170008891
O	9.66785018952127	13.61061321366599	13.56547174215077
O	12.44966979925674	13.56705106620776	13.56886446285080
O	15.08177535004604	10.81167766081809	13.56763211781356
O	15.04841071277378	8.04009302252109	13.55764203824477
O	9.52727224159146	5.43912118726869	13.55244021340335
O	12.31966175942366	5.39957676949785	13.55698427315610
O	6.85960353303041	8.16315432245124	13.55844357705882
O	9.62951830512761	8.17270168693346	13.47783428241374
O	12.28637982559801	8.13434997872795	13.48492467910344
O	12.33250304492261	10.79791573949236	13.50310497750216
O	9.67534570389545	10.84255307005415	13.50099923336125
O	12.60374908899809	16.09974447805091	10.95828927212696
O	9.69848384602739	16.17087127388897	10.95701070378212
O	12.48772983334927	2.95978294599037	11.08288718106335
O	9.31160330205806	3.00761665113978	11.06938148332774
O	6.83486600104825	5.50438551006728	10.87527427759219
O	6.99757029824697	13.63635858625899	10.88209618190652
O	15.21583359388593	13.38733227259520	10.84185054738039
O	4.37755234231214	11.01963390247659	10.91015953118333
O	17.62302951751863	8.14457749242725	10.93730351986053
O	15.05147336213001	5.39183310868072	10.87317175003732
O	17.49664116685003	11.00832203517417	10.86421102305511
O	7.01564189320039	10.90424189028529	10.85800054263779
O	4.33359090140128	8.18224928605161	10.92292952928292
O	9.60487926520212	8.16458787830370	10.85424477843951
O	12.31920896310575	8.11151115771172	10.86515532054035
O	12.34160057080890	10.83877327712122	10.86572272452525
O	14.94297330467089	10.73286463267072	10.84415363128754
O	9.72465326280256	13.51457389163953	10.87279220438730
O	14.95248816619266	8.06932307503825	10.84543985188708
O	12.45676103632665	13.46300079048084	10.86895455590573
O	9.60277148643170	5.52707493693292	10.81715028532884
O	12.26569778311045	5.48415572189857	10.82005806558117
O	9.64219730250059	10.89394878817594	10.86365400122423

O 6.98333728335829 8.21123889639098 10.84651830964236
O 9.64397543304394 10.90098979148382 8.15620098310305
O 6.98217735668873 8.21998413938190 8.15709752520957
O 12.45349776382679 13.47133192406276 8.15723177725752
O 9.72518369293665 13.52309008603591 8.15764045254610
O 9.60596728702235 5.54300385582464 8.15294976621512
O 14.94655034700823 10.73925894162770 8.17351466278245
O 12.27305676362667 5.50116796945290 8.16023483865529
O 12.34332121036021 10.84691366087086 8.15095779486016
O 14.94857793540926 8.07487471718157 8.15182490947418
O 9.60313529488504 8.17510081587661 8.14579271604937
O 12.31791568871505 8.12249916491606 8.13448995683048
O 7.01749570506107 10.91144260015318 8.16561384576045
O 17.50262748173345 11.02486510496007 8.16539342461091
O 15.21407692290820 13.39294323516229 8.19390738703052
O 4.33329465982682 8.18800279403388 8.08408438152477
O 6.99894885490276 13.64467801419317 8.14914316791364
O 17.61534303974675 8.15378044748556 8.06914422911748
O 4.38242388095550 11.02475050752454 8.11301937862116
O 15.05685847866640 5.39869100636717 8.11321646657395
O 6.83604844230986 5.51302636343090 8.10241477751044
O 9.69771852371760 16.17843960297903 8.08086796987269
O 12.60400159596965 16.10825662301203 8.07831567861303
O 12.48277204963146 2.98018633488770 7.89884862955076
O 9.31334415176904 3.02620010616305 7.88086328789585
O 9.67653320412536 10.86465945458731 5.51678239363921
O 12.33663265305978 10.81537130540158 5.51294556400814
O 12.28504443567360 8.15190110248414 5.51300300260656
O 9.62703147302696 8.19963467031448 5.51689491427127
O 6.85697497231849 8.18267076198751 5.44436887303976
O 15.08815278131162 10.83522453498303 5.45006040158446
O 12.45616708389954 13.58641028515806 5.45882127098412
O 15.05017229775060 8.06436514747865 5.43792517860278
O 9.67531534910212 13.62926808105530 5.46523204128371
O 9.52610308343136 5.47054718722798 5.41792681051889
O 12.31878811777605 5.41988395713877 5.42376634744240
O 6.92340261809543 10.95627034835959 5.46437897251417
O 9.43027234246889 11.12355738746391 2.99354739653316
O 12.58165226381562 11.06737976751044 2.98689066019408
O 9.35965259275833 7.94113585694821 2.99708651632521
O 12.54026344170460 7.88019316525969 2.99277128694112
Pt 11.15450395029821 16.24624195797394 9.51856300627155
Sn 16.12356448035824 11.97091596098660 9.51511717056966

$Pt^{2+}/Sn^{4+}(Ce^{3+})_2(Ce^{4+})_{37}O_{80}$ E = -952.94479 data in Supplementary Figure 1

Ce^{3+} 18.68409421627767 9.34676164543494 9.50225819345558
Ce^{3+} 3.30226058933854 9.58467229465890 9.51217331253705
Ce 13.52660545682259 9.41603705884853 14.93491036034542
Ce 8.43701294377157 9.49642507636775 14.92384486920922
Ce 10.94681588906070 6.85138502277562 14.85987815892209
Ce 11.02715787959841 12.06330086985663 14.94180292200312
Ce 8.32606907174848 12.24174931340014 12.27841090063388
Ce 8.26300917679130 6.77362367402426 12.23813352667410
Ce 13.62254471318148 6.67185577922445 12.26146393752737
Ce 13.73446407417313 12.16372197152302 12.30080840779813
Ce 16.19399755787609 9.37306799105753 12.20343038923600
Ce 5.80321710630361 9.54327382597020 12.17664398396556
Ce 10.96874851423742 9.49145986837605 12.14236583413758
Ce 11.06551873632771 14.75949846448720 12.22424177300536
Ce 10.90213355496132 4.10589292655783 12.09738688108971
Ce 13.70633155934143 14.74859046212169 9.52455206740930
Ce 8.42148305689279 14.82659625662712 9.51822594624937
Ce 5.80791814421066 12.19780601080791 9.51255792043047
Ce 16.27623321598078 12.06466246674138 9.51723290417216
Ce 11.01186172027201 12.14266267106418 9.51455951912703
Ce 13.59315935526189 9.44800656611149 9.50530877873637
Ce 10.93514143859659 6.86405781208782 9.49151164702102
Ce 16.14329043846758 6.69916000046937 9.49728350508934
Ce 5.77437300393577 6.89412321268569 9.48718629293055
Ce 13.44880243506006 4.03629835888520 9.49392096026428
Ce 8.36603194777466 4.12281657066123 9.47604236187992
Ce 11.07360879863343 14.77460609546713 6.82080525447832
Ce 10.91326956726518 4.12500958334744 6.87310376830605
Ce 10.97781467436413 9.50874733637845 6.86544310010656
Ce 5.79649724726901 9.55518211688846 6.82911582384035
Ce 16.19888066013190 9.40132461971083 6.80520961641450
Ce 13.73976115567317 12.18551931693038 6.72939191529207
Ce 8.32889505652365 12.25196567081543 6.74854993228866
Ce 13.63190037969596 6.68778374181214 6.72984382400228
Ce 8.26671853842453 6.78965682541726 6.73779079268635
Ce 11.03570933313829 12.09024928972879 4.08408111748722
Ce 10.95079699980278 6.88971274868769 4.12732974183399
Ce 8.43850115745632 9.52770829494148 4.08346528285762
Ce 13.54107662407930 9.44550010830310 4.07415793821228
O 12.54271791532699 7.85612566030775 15.99665157662774
O 12.58203112969658 11.04768289461497 16.02311808888689
O 9.36635893374461 7.91258904726720 15.98263686732700
O 9.42588398400201 11.08930623558904 16.00893008165999
O 6.92485769838168 10.94406034917020 13.52910581349784
O 9.66137139938133 13.61605339122619 13.57041337566947

O 12.44070694553656 13.56838646199306 13.57997165854796
O 15.09043049120177 10.80921555947381 13.55497933743909
O 15.06230837895455 8.03531425021441 13.55475763342605
O 9.54310484552002 5.45002113763913 13.55334911565041
O 12.32693448071241 5.40432153256446 13.56503424174252
O 6.87299424062119 8.15538539367636 13.52545166633713
O 9.64133576363501 8.17744758449435 13.45883805049144
O 12.29022856870676 8.14017350941646 13.47533035442108
O 12.33352862451121 10.80489678365168 13.49505415506557
O 9.68362048490709 10.85390406178224 13.47754310879234
O 12.52354526675976 16.12659079320562 10.96744257566959
O 9.62648717034829 16.15595514182368 10.96573409933529
O 12.46627944589714 2.97474004124676 11.08604232348774
O 9.29933094698817 3.03376547544576 11.06599119461202
O 6.84073741245756 5.53923283106028 10.88257361430467
O 6.97230791389446 13.61092636080261 10.88831982637866
O 15.13006703388438 13.50273567416979 10.88991156585116
O 4.40297138002485 11.00369745841410 10.90988057426409
O 17.58500733392970 7.96435651472915 10.91821686935664
O 15.01194056861561 5.37684248532363 10.88167686307069
O 17.63227856176063 10.79895093186531 10.91402450043907
O 7.07829730779265 10.84821670478960 10.79920218162126
O 4.36474624618784 8.17680241514923 10.91404545422655
O 9.55789700089685 8.22995456339986 10.79971249915317
O 12.30846923953651 8.13331440069549 10.85512955505761
O 12.34784869609679 10.85164905024526 10.86373805288263
O 14.98682459995044 10.77458974450165 10.85370121980687
O 9.69719487247755 13.49645007110131 10.87647038218271
O 14.93566879465327 8.08613551600136 10.84149960156035
O 12.40192985676609 13.46842337564980 10.88034551920315
O 9.60437347127614 5.56036200566656 10.82054674951048
O 12.24776751253268 5.50465220817541 10.82511206856143
O 9.59329207088910 10.84274188462275 10.80682491043660
O 7.05056543813668 8.27706300328646 10.77832605574843
O 9.59922868607151 10.85039936938471 8.20772863797161
O 7.04960753186733 8.27414939171124 8.20601093805915
O 12.40364783642820 13.47571402568870 8.16100369368735
O 9.69812342832425 13.50357622628595 8.16039948774259
O 9.61151578210623 5.56963344048711 8.14921991435248
O 14.99117187485139 10.78553831878043 8.16674551956329
O 12.25896151358871 5.51428967646639 8.16019281180894
O 12.35125765065441 10.85988690778949 8.15568961751902
O 14.93984088277890 8.09729304729499 8.15761684101813
O 9.55783584950383 8.23873319386235 8.19133708114104
O 12.31156371592206 8.14027898941356 8.14293328677622
O 7.08267555955160 10.84358982248008 8.22257346333750
O 17.63954713673028 10.81638342592753 8.11352976379127

O 15.12968570698187 13.51417546924380 8.15206603900753
O 4.36366652290572 8.18173935552930 8.08142690237372
O 6.97313407443617 13.61209257591628 8.14211741005735
O 17.59237611529889 7.98344250773303 8.07667362004726
O 4.41129211996856 11.00708747075298 8.10842003247475
O 15.02192821215053 5.38530655316538 8.10581405146679
O 6.84565395611777 5.53964827165182 8.08865795454073
O 9.62945632531645 16.16378464494182 8.07813000672792
O 12.52889647325934 16.13423196663408 8.08472778948740
O 12.46432640430100 2.98504358596365 7.89523824251210
O 9.30058219511249 3.04480952397750 7.88276733165995
O 9.68787713554282 10.87641513649717 5.53879860500678
O 12.34414712702917 10.82876087829492 5.52250427945094
O 12.29749971100285 8.16341137705336 5.52330253847327
O 9.64811359432867 8.20485820128354 5.53492682680291
O 6.87460886174748 8.17769734303076 5.46543098335612
O 15.10336877849248 10.84336674630242 5.46417870275482
O 12.45453445994698 13.59352143840629 5.46032597047582
O 15.06928116262087 8.06840781753071 5.44120172523897
O 9.67440008647386 13.63406369380314 5.46715092286039
O 9.55110404415421 5.47780436207007 5.41666853182720
O 12.33586335376742 5.42902737143642 5.42063291941705
O 6.92595807979395 10.96496800274974 5.48876736210541
O 9.43605253374250 11.12272327870749 3.00907800350791
O 12.58871278235839 11.07960777035920 2.99591358469936
O 9.36533099347287 7.94920564483962 3.01188339126204
O 12.55255527020611 7.89513244950221 3.00064044689575
Pt 11.07891965801281 16.25388858781271 9.52418960270851
Sn 8.39404932632847 9.52828771608747 9.49982832207435

$Pt^{2+}/Sn^{4+}(Ce^{3+})_2(Ce^{4+})_{37}O_{80}$ E = -953.09469 data in Supplementary Figure 1

Ce^{3+} 18.68366707921862 9.34252261945428 9.50096576195867
Ce^{3+} 3.29108261936127 9.58748648518754 9.50735647629408
Ce 8.39463953527153 9.51296506874543 9.50371708239324
Ce 13.54264259494249 9.42561118784674 14.93013255951106
Ce 8.42817267995957 9.49607256495878 14.92781153649907
Ce 10.95181765108038 6.86324433224773 14.85843326809096
Ce 11.02015820693511 12.05403172235083 14.94229994661271
Ce 8.32362979812984 12.24849785910167 12.29680824215956
Ce 8.28325774324240 6.75594566457967 12.23455328915712
Ce 13.60780668074342 6.67612784998740 12.24174046810234
Ce 13.73537316428843 12.16146663294198 12.29749763382602
Ce 16.19356227275123 9.37731741392133 12.20668533340821
Ce 5.78338400356412 9.53940010946718 12.20870382383634
Ce 10.98688341469064 9.47091674252051 12.14094973687685
Ce 11.06575406727393 14.76075825596994 12.21354507304584

Ce 10.90623313511015 4.12848030278555 12.08170496465319
Ce 13.71975376514810 14.74810402801301 9.51888904857325
Ce 8.41949132487151 14.83022648040502 9.51562301175351
Ce 5.78433043393162 12.22019722268247 9.51006496650963
Ce 16.27512021681934 12.05797986048396 9.51606558773058
Ce 11.02856607624900 12.15079513684470 9.51334394109469
Ce 13.58103913045018 9.43148821663172 9.50241777516671
Ce 16.13785130361788 6.71014814040772 9.49848588756511
Ce 5.75605779570578 6.87187563209335 9.49525582149148
Ce 13.44653384164786 4.06602423045646 9.49185893517753
Ce 8.37144346176503 4.14233627357131 9.48103465519212
Ce 11.07210070443103 14.77190149749485 6.82102855277862
Ce 10.91173744542918 4.14310813139650 6.89185768036044
Ce 10.99398027872745 9.48877220804958 6.86604456794939
Ce 5.77725567945714 9.55187941043863 6.80058754247938
Ce 16.19707295887938 9.40421243166171 6.80175302568649
Ce 13.74089575708322 12.18049098110150 6.72471972299896
Ce 8.32492801282862 12.26058774180573 6.72772072004173
Ce 13.61191088515154 6.69160345888241 6.75494766366322
Ce 8.28556367648827 6.77299981098172 6.75505093538385
Ce 11.02623571213636 12.07691125790777 4.07897708184746
Ce 10.95096887853066 6.89614442731551 4.13190172436900
Ce 8.43085981129839 9.52621371842398 4.08643558609339
Ce 13.55108078438083 9.44896682671402 4.07453360799802
O 12.55052175195236 7.85799013990358 15.98289413274932
O 12.58769930411128 11.04529761407607 16.01994348530349
O 9.38289644756607 7.90433818844229 15.98259715754519
O 9.42707191820653 11.08976968813158 16.01968198027314
O 6.92337851860403 10.93819366308804 13.56176994892888
O 9.64737103108704 13.61221557162047 13.56860485879593
O 12.43070944313545 13.56577050629968 13.57029038669534
O 15.09308458214210 10.82108099633063 13.55933388774238
O 15.06655742988785 8.05079670705029 13.55591981723595
O 9.53524664838549 5.45166496492451 13.53467174792345
O 12.32159609859559 5.41175471472377 13.54391992453900
O 6.86899179190873 8.17093545680494 13.55537916944994
O 9.63397923879903 8.18232927262857 13.45773644069465
O 12.30272952629150 8.14387561677738 13.45742445504990
O 12.33655565705086 10.80190813101829 13.49238909709754
O 9.67805886068677 10.84275746222559 13.49210365025564
O 12.53831060068897 16.11959520348373 10.96353133165962
O 9.64216508533742 16.16283385214457 10.96282571156872
O 12.49782665289172 3.01450405284416 11.10370610073423
O 9.28643619761077 3.06428559525637 11.09441859598448
O 6.85451601465427 5.51129265415217 10.87535060146128
O 6.96654650342737 13.63038850012050 10.88048940573953
O 15.13250345465979 13.50291262280235 10.88727206854246

O	4.37672170862620	11.00181685983984	10.90846095714146
O	17.57428996887387	7.96010015762086	10.91473774297281
O	14.99697586209568	5.38622489641184	10.88047240770740
O	17.63890082468015	10.79367413115620	10.91241107963731
O	7.02305607216405	10.89880516392826	10.85597417002848
O	4.35789679589122	8.16967594002373	10.91715961016437
O	9.66479563460013	8.12030149273478	10.80067900296265
O	12.26352692685773	8.07857031497087	10.79538494555624
O	12.36699539644027	10.83292461737304	10.86120808491384
O	14.99354061780397	10.77372644444438	10.85432240230056
O	9.69323770900449	13.50483936205539	10.87108900408475
O	14.92111803293312	8.09113945838713	10.84769403260167
O	12.40433800504415	13.46182540423968	10.87344311341740
O	9.66374543239881	5.59661754289567	10.76048380104156
O	12.19710877935475	5.55655866498659	10.76844385564207
O	9.65037767425254	10.87766478024004	10.86233662620039
O	7.01386892029780	8.21453650911262	10.84611714614531
O	9.65178823211276	10.88516898268750	8.15361010299288
O	7.01231049695855	8.22218604491396	8.15161604176361
O	12.40529893523602	13.46921305788173	8.16020794427541
O	9.69484711867564	13.51118172151823	8.16041303346442
O	9.66522168762917	5.60227276732800	8.21754665919934
O	14.99596565396218	10.78365146525819	8.16208799771410
O	12.19874030072443	5.56610632580236	8.22362650108044
O	12.36862828607440	10.84224670554455	8.15417240300455
O	14.92686945760065	8.10014860408543	8.15195329740823
O	9.67043985178092	8.12704499447894	8.20119434097993
O	12.27257843000177	8.08710255777284	8.19331282819214
O	7.02340683407597	10.90421923104349	8.16117296069865
O	17.64430867477682	10.80896834243669	8.11209133431031
O	15.13342014062499	13.51197904858648	8.14934358761196
O	4.35163883080898	8.17459949366181	8.08571249047873
O	6.96715544880114	13.63540617323279	8.14348883020760
O	17.58215596178994	7.97512749059081	8.08276617243649
O	4.37851230209360	11.00450670233869	8.10667849093311
O	14.99904563588767	5.39339702480469	8.11218267971394
O	6.85668035009015	5.51896211108927	8.10657094615411
O	9.64276077907723	16.16912590517145	8.07510308872483
O	12.54120191162762	16.12683856995378	8.07794207086102
O	12.49903900829313	3.02610655777588	7.87055405422874
O	9.28462068756809	3.07200495905952	7.86172221826472
O	9.68264878657157	10.86338747282041	5.52312652923460
O	12.34506591963177	10.82406594330893	5.52125141856368
O	12.30514846467836	8.16626625880262	5.53839772877017
O	9.63630711318933	8.20757058900591	5.54401843492700
O	6.86908207087977	8.19323756977376	5.44429327513065
O	15.10466403937203	10.85446029140948	5.45750664625869

O 12.44005832855290 13.58928255291550 5.46113033580436
O 15.07030388496308 8.08306463396708 5.44228834797499
O 9.65654197976003 13.62780999797296 5.46202172279958
O 9.53706238018474 5.47750551808335 5.44193446723177
O 12.32679459429690 5.43385663673902 5.44661685349662
O 6.92425398817296 10.96057829286857 5.45557761655768
O 9.43294286559311 11.11916054790580 2.99790682753081
O 12.59153411813245 11.07355859497568 2.99475213349638
O 9.38680600565820 7.94008372286563 3.01763843225694
O 12.55849947116445 7.88986830585072 3.01366972383288
Pt 11.09259834559075 16.25271577029764 9.51965126521889
Sn 10.94926345530855 6.84213761287610 9.49547298939807

$Pt^{2+}/Sn^{3+}Ce^{3+}(Ce^{4+})_{38}O_{80}$ E = -953.75488 data in Supplementary Figure 1

Ce^{3+} 3.27896222610770 9.58815437140534 9.51120245042822
Ce 8.37586403578934 9.52930088630615 9.50751692376637
Ce 13.53564178533751 9.42392824454019 14.95154268660000
Ce 8.43036985483578 9.49258491145553 14.93143584916561
Ce 10.94993119664976 6.85770732997414 14.86743297395961
Ce 11.01761406118567 12.05999695831748 14.95126547190678
Ce 8.32188891251492 12.25052940026183 12.29565985848362
Ce 8.26533634850018 6.75012725723632 12.25610613325990
Ce 13.62979897879388 6.67143929237019 12.26524688825390
Ce 13.73660947730618 12.17103168021338 12.30137608769356
Ce 16.20220333332797 9.38453140255129 12.22307584077929
Ce 5.77412811225057 9.53525722858115 12.20537958946971
Ce 10.99412336995173 9.49098587811707 12.16187433067978
Ce 11.05996441791265 14.76664749968111 12.21436452713259
Ce 10.90403442212688 4.08991572014565 12.09069436755905
Ce 13.71154569061916 14.76673135208987 9.52212383524927
Ce 8.41502111162231 14.83424284832236 9.51555553206003
Ce 5.77243108658935 12.22490832882845 9.51038615154540
Ce 16.27907758242099 12.08967003397509 9.51260324937850
Ce 11.03231332901391 12.15991363326571 9.51287225845123
Ce 13.61639545227864 9.44932367466847 9.50531487347004
Ce 10.95705854132122 6.84654330947846 9.49610102320524
Ce 16.15389913563120 6.68849172855624 9.49673069381888
Ce 5.74520632911170 6.86148202913585 9.49326868792647
Ce 13.45913403417060 4.02585767879710 9.49132530209589
Ce 8.36337643646461 4.10840982281014 9.47401693878361
Ce 11.06686396999275 14.77913514963502 6.82114381353621
Ce 10.91300901372531 4.12266848630897 6.87476662755136
Ce 10.99720758550878 9.50558543658857 6.85139531352396
Ce 5.76643529853774 9.54824438799420 6.80628522753313
Ce 16.20516048668006 9.40949050962470 6.78584942963172

Ce	13.73830918590146	12.18941877253483	6.72360289357467
Ce	8.32326437534415	12.26073159055867	6.73172559522433
Ce	13.63688832459396	6.68842592308607	6.72652957615709
Ce	8.26301393211762	6.77146041551167	6.72786426105699
Ce	11.02509125077666	12.08099364929236	4.07476779746111
Ce	10.94213183338966	6.89327877097485	4.11799147623050
Ce	8.42923026526873	9.52486090739053	4.07919308908199
Ce	13.54591368893936	9.44384403444544	4.06056126884918
O	12.55248566705599	7.86408265590058	16.00297619407598
O	12.58173936666576	11.04886282402207	16.03045305412530
O	9.37859295583115	7.90470725406585	15.99264284729087
O	9.42780959375893	11.09246965847647	16.02209137425935
O	6.92652538750694	10.93072829596253	13.55712557955543
O	9.64632468666121	13.61354271589851	13.56915139114101
O	12.43036517652877	13.56898391827096	13.57003675648150
O	15.09642797646601	10.81951185019442	13.56087188652039
O	15.07176177027046	8.04139568797476	13.55926205479174
O	9.53261921145604	5.44530568848888	13.55413246527207
O	12.32396472381427	5.40976686110447	13.56096630082327
O	6.87236154664078	8.16088926343106	13.55503884421029
O	9.64301888569908	8.17960016264989	13.47546049031147
O	12.30075965155598	8.14332855803503	13.48344656745783
O	12.34020650776211	10.80857126129730	13.50185164510555
O	9.68052130952743	10.84621265360630	13.49714615921925
O	12.53000814283737	16.13217673939021	10.96521980324394
O	9.63574929351575	16.16897357568313	10.96391334058891
O	12.47439787019830	2.96644203422928	11.08207449973783
O	9.30934849616275	3.01509687781435	11.07025141975758
O	6.84088400628328	5.50403876101671	10.87365702596453
O	6.96499220222373	13.63058942273567	10.88041977516897
O	15.13483544442783	13.51270556371894	10.88688884018051
O	4.37869617927170	11.00484567601895	10.90957788818051
O	17.61195898773975	8.00051267595749	10.89061294916549
O	15.02194824602818	5.37688571377265	10.88086851189727
O	17.65422773723645	10.77455101609728	10.88771197277190
O	7.02035347601696	10.90000776371490	10.85522566944526
O	4.34501642868303	8.17365929532715	10.92295597036254
O	9.61809183210540	8.17201344268536	10.85520162592011
O	12.34097265679270	8.13291077770457	10.85620601612515
O	12.38002795901629	10.85201342758778	10.86201682497381
O	15.01648442998845	10.78756197623909	10.86135306965996
O	9.69192055066072	13.51390721560735	10.87200218465057
O	14.96639695620670	8.08409531963821	10.84844780606243
O	12.40684905876743	13.47654568842872	10.87456152352487
O	9.60544613411365	5.53626126221297	10.81600079385276
O	12.25862078551090	5.49618931114772	10.82543051703237
O	9.65628987061891	10.89349402008720	10.86300628198504

O 6.99324416751679 8.20929190869916 10.84214677365061
O 9.65699712930728 10.90016296317375 8.15642732839840
O 6.99039730110038 8.21771372438457 8.15517715860307
O 12.40793621575466 13.48283855195386 8.15910224566375
O 9.69412889695982 13.52012441444771 8.16077135446007
O 9.61129023041020 5.54978027334956 8.15501723971817
O 15.01660796073549 10.79747523564483 8.15679090992568
O 12.26897981851130 5.51154813142817 8.15956070324793
O 12.38106413611987 10.86135325176849 8.15685171334912
O 14.96836131859464 8.09530051571338 8.14778834300402
O 9.61738013403120 8.18039504961681 8.14593903190506
O 12.34282393536478 8.14364605262888 8.14604849322695
O 7.01903938372402 10.90502472937562 8.16392713111993
O 17.65503480565466 10.78986885908143 8.13662072501576
O 15.13667867412826 13.52412498856644 8.15015387031488
O 4.34005868079222 8.17994626439447 8.08271631399759
O 6.96674545432042 13.63484672338262 8.14312694880235
O 17.61168652183384 8.01695898460844 8.10678042019068
O 4.37703080308784 11.00956166753137 8.11100093544621
O 15.03148283738830 5.38679068082823 8.10182396005280
O 6.84363115841490 5.51265785608610 8.10025331621580
O 9.63825606506978 16.17526500327451 8.07432607657426
O 12.53343801945312 16.13819948314847 8.07901226909969
O 12.47373932255304 2.98356066338522 7.89713645644651
O 9.31187253946918 3.03185684500684 7.87618951718469
O 9.68319834779953 10.86595532270837 5.52087901718924
O 12.34762206335687 10.82792814620112 5.51711858138059
O 12.30089620888512 8.16276241761733 5.51821205841669
O 9.64299392486143 8.20628843580175 5.52159699973330
O 6.86737968061837 8.18017659757859 5.44260621578125
O 15.10618218624144 10.84987976758137 5.45641646714126
O 12.44040955557970 13.59099843947070 5.46111306168116
O 15.07356006546917 8.07253216205895 5.43350012801695
O 9.65675236484098 13.62905804091493 5.46420710922226
O 9.53285723866437 5.47598163057089 5.41653059431991
O 12.32606372213342 5.43230805029264 5.42430626627804
O 6.92515608741108 10.94937703685246 5.46281340220300
O 9.43331637005429 11.12309124674640 2.99675148565585
O 12.58516178938439 11.07194315886804 2.98937822284908
O 9.37089821305396 7.94199383602223 3.00164393894390
O 12.55492861338777 7.89404228141923 2.99629304813107
Pt 11.08500077696458 16.26227235349808 9.52066826699935
Sn 18.45056817534395 9.36131085364968 9.50536908501592

Data for structures displayed in Figure 10

$Sn^{4+}Pt^{2+}/Pt^{4+}(Ce^{3+})_6(Ce^{4+})_{33}O_{80}$ E = -957.43345

Ce^{3+} 18.71133701383881 9.35577000354967 9.42581340880334
Ce^{3+} 3.27494436124967 9.60633681302972 9.50639244279467
Ce^{3+} 13.78788723746968 9.40139181942719 14.88798688325788
Ce^{3+} 8.23745408267211 9.51525532749448 14.87922359757285
Ce 11.01252705852828 6.67899349077915 14.80292470476063
Ce^{3+} 11.01874648731828 12.20064843126745 14.86935016261226
Ce 8.29453706467851 12.25794129625005 12.17299119830932
Ce 8.30828347467105 6.74922630351403 12.26150953286062
Ce 13.70745070341622 6.64165448691213 12.24407129281581
Ce 13.73693747751663 12.16995008461139 12.26000583296799
Ce 16.27271909494496 9.38712547735726 12.13747861999307
Ce 5.84201654636614 9.57963683496974 12.03100406893865
Ce 11.00280291880347 9.43854516057398 12.28199310630880
Ce 11.05549569883409 14.77102818558362 12.13488121280379
Ce 10.91562214908632 3.99896393763511 12.05854339594891
Ce 13.67475926327445 14.72654710685376 9.46481452848498
Ce 8.38598292825763 14.83596928003973 9.42982519333252
Ce 5.69498451064392 12.30464866721947 9.35653124746587
Ce 16.25995751309221 12.06640681106308 9.47450596097759
Ce 10.94048169879983 12.09311841083686 9.49467452746892
Ce 13.58053895549265 9.44638557678836 9.52726476190261
Ce 10.95050731426243 6.85726011822731 9.59058997156049
Ce 16.17221797134044 6.71066207798723 9.45981916914063
Ce 5.83842755075355 7.01421476341899 9.43389737746969
Ce 13.46051192114178 4.02680478480798 9.45796134860647
Ce 8.35742673517755 4.13969667796462 9.48718237213565
Ce 11.04973935421918 14.75652763653158 6.77587845271399
Ce 10.88743870345398 4.18149581054884 6.88608827151048
Ce 10.93138751474139 9.51847866410223 6.90882726362728
Ce^{3+} 5.65425778481954 9.65827022905073 6.64957365739765
Ce 16.17773826728655 9.40652551226277 6.78326853199389
Ce 13.71455014782715 12.18040570088585 6.69934554315934
Ce 8.22878927829959 12.33894113419992 6.57996038897820
Ce 13.61677823425254 6.70610673213121 6.74932986432841
Ce 8.23818851053091 6.84938011338283 6.70995011975835
Ce 11.04835312328783 12.08392369768785 4.06006374802154
Ce 10.96267448505309 6.89403670350377 4.14891101532238
Ce 8.43444687569850 9.54830376196516 4.09781498177812
Ce 13.54138408629515 9.43897759343177 4.08014515521903
O 12.34874714989946 7.93260398635336 16.30119436102907
O 12.40141461294818 10.75021172154204 16.42682138056674
O 9.60907853867428 7.98491815086529 16.41123159554249
O 9.63703777233319 10.81756010867920 16.36042242146735
O 6.87289717149208 11.01409213384344 13.39189372345892

O	9.62051998768914	13.66642438782347	13.47204598199365
O	12.47729170946400	13.62253793216284	13.50503891683709
O	15.20607840790210	10.83067858620803	13.49994485254449
O	15.19294761350134	7.98114370869552	13.49933753745604
O	9.61249808387033	5.39002681851922	13.61953687444028
O	12.38713323124253	5.35112833521163	13.63140375138349
O	6.84653321862857	8.14154093395838	13.43620517753015
O	9.65511508811975	8.12270237722646	13.75196328783943
O	12.37326859789763	8.04481460897701	13.59725267584797
O	12.42072522225012	10.82973179290822	13.63241380438796
O	9.63528523979243	10.89466624729802	13.53688130480022
O	12.52501028727593	16.11617596682839	10.91462280183492
O	9.62760801814170	16.14532493166221	10.90518807485515
O	12.46720693901803	2.91598461039950	11.04199399599438
O	9.29201934855640	2.96577705627381	11.07834810034208
O	6.89096562122795	5.59397196335420	10.85311376542409
O	6.95885130656465	13.59738325237830	10.85314564954692
O	15.11275027171764	13.48607015735104	10.85621653043775
O	4.44131077601497	11.00237206898649	10.91080016596623
O	17.65880346514511	7.97929986991176	10.86252527308699
O	15.05246504864376	5.37287537714877	10.87087346472203
O	17.65704675808540	10.80529702024314	10.85086724912990
O	7.08547879381890	10.87533966696975	10.48526834681291
O	4.48894348562058	8.18812156716911	10.91039650626170
O	9.83091848588882	7.93245136550608	11.21915251875633
O	12.41764557993926	8.13112405653006	10.93502791612837
O	12.30635337080464	10.83425895270257	10.93771056578175
O	14.98813102108756	10.77124822815903	10.82481271904793
O	9.67578287577568	13.43774892477371	10.80055610899497
O	15.00113006144269	8.08208852588415	10.81313306448762
O	12.37324719696309	13.43703054096893	10.81573561386606
O	9.62993032350662	5.43725763776743	10.82535171387029
O	12.29005712456699	5.46818694041237	10.87433496947944
O	9.57540166965265	10.62586900234206	10.79191010231209
O	7.29815543753426	8.32235566250804	10.72342480473640
O	9.35688197683140	10.85935030314391	8.20494632175378
O	7.07840144095147	8.56662704693804	8.18282645526351
O	12.36919819843506	13.44235403339542	8.13026018587635
O	9.67074823313404	13.47114423431310	8.07647547686247
O	9.60257974220666	5.62802433865110	8.16906928871242
O	14.94580628955808	10.78524069223197	8.14917874506147
O	12.25770545658202	5.52209393120453	8.20218344752071
O	12.27586410602651	10.84115239509909	8.19516335304655
O	14.93315849611111	8.10275547609388	8.15633013869058
O	9.56574684488536	8.31401379183322	8.44440406369118
O	12.28977680273112	8.14035625428046	8.18524072790182
O	6.73967055679861	11.29122280915841	7.80068852503706

O 17.61140245780345 10.82124291078340 8.06196296731275
O 15.12228179001435 13.50857186348628 8.11174088368976
O 4.33936871687151 8.16962696380393 8.13691745649532
O 6.92343839024901 13.90001392730713 8.10493495335300
O 17.59974118092239 7.98651056982143 8.03276709771378
O 4.12937615074344 11.13348555815854 8.16388883497665
O 15.03071179842681 5.38860747471723 8.09618209237685
O 6.83072905341128 5.65248133622779 8.04160257639197
O 9.64558566606476 16.16703839736889 8.01056423476992
O 12.53576337591991 16.11415943910044 8.02734647269819
O 12.46745845837851 3.00233462310658 7.88195560389855
O 9.28209641285511 3.07676575030534 7.90774979872596
O 9.71044194989874 10.91816666431095 5.56051075775716
O 12.33655533742444 10.81239348994625 5.54505082344490
O 12.28682667477687 8.17287795617163 5.56046314758432
O 9.66358005171901 8.22163606173795 5.61178008149737
O 6.95249847976223 8.12252555636110 5.38923840187938
O 15.10330508791313 10.84307142337227 5.44436678681564
O 12.46315319077194 13.57782207077902 5.42721059923113
O 15.06317124588247 8.05717499256710 5.44306885811944
O 9.66864384903748 13.69466949801207 5.36248411762014
O 9.55036377320863 5.48673179606965 5.43523925461487
O 12.33352829691770 5.42680888788025 5.45541510965766
O 7.02364097604799 11.01442136804052 5.15374130211435
O 9.51645262677402 11.11765745896770 2.97874922280576
O 12.64962982750624 11.07106091622544 2.99768783356540
O 9.42435435144221 7.93881411192403 3.04757763160103
O 12.58487197891574 7.86308938262472 3.01944117510111
Pt^{2+} 11.08117943163724 16.24745238744103 9.46365465335012
Pt^{4+} 8.34262612267900 9.59247496490905 9.45776585990473
Sn^{4+} 10.99391710371579 9.37750649777760 16.87818409941223

$Sn^{2+}Pt^{2+}/Pt^{2+}(Ce^{3+})_2(Ce^{4+})_{37}O_{80}$ E = -957.63497

Ce^{3+} 18.71025599858076 9.36976455621255 9.46027085478611
Ce^{3+} 3.26338826703986 9.61657385939355 9.48326455166857
Ce 13.60810796541734 9.42917347068780 14.80455396478192
Ce 8.36617237237152 9.51833382613170 14.83837504716915
Ce 10.95908539515336 6.79911012062114 14.77840167703701
Ce 11.03985536139331 12.11041377108621 14.82720248946396
Ce 8.27881776929680 12.30716563070338 12.31384419849725
Ce 8.26680883693225 6.77673870640392 12.17744036928697
Ce 13.64525278345353 6.65607310546901 12.20431341555094
Ce 13.74462261975729 12.17792023274544 12.23022212913611
Ce 16.24908055950240 9.39447332685337 12.14803325882792
Ce 5.74771148391491 9.46202266653796 12.16438871648925
Ce 10.95592738641554 9.43473704260577 12.11716365975334

Ce 11.05677905249829 14.81393651732321 12.16354063935793
Ce 10.90724239712926 4.05521609514258 12.05682663165927
Ce 13.71308231165691 14.78189454674603 9.47687044123466
Ce 8.39193168728150 14.79690237700122 9.48853303222410
Ce 5.70155685865660 12.25578768813178 9.49383950890383
Ce 16.28178491431892 12.08629634160116 9.47158470581271
Ce 11.04404399863132 12.17740180029234 9.48857752521680
Ce 13.60332718851060 9.45308504419018 9.48032814629628
Ce 10.88137847689082 6.88538872422415 9.48019287858832
Ce 16.16955300390056 6.71239743504103 9.46079861136913
Ce 5.83115153045573 6.92517966572539 9.46507054823178
Ce 13.45244186576235 4.04084795797747 9.46794005331302
Ce 8.35342667223677 4.08770685190634 9.46707574400549
Ce 11.04192776511538 14.78097901109284 6.79172734915170
Ce 10.91116941313696 4.12227520614756 6.87481307619622
Ce 10.94809502628066 9.45319094390651 6.84664022030641
Ce 5.79473384598793 9.47829512188486 6.78394564810075
Ce 16.19691272665131 9.41285545966052 6.77304735836126
Ce 13.70395892924462 12.17156842032489 6.69485378627825
Ce 8.28960000187976 12.28782557089396 6.63698427687677
Ce 13.61998296108653 6.68471467169588 6.72369170456675
Ce 8.29333337647312 6.80492963431560 6.76355635133686
Ce 11.02078164594539 12.05420224248521 4.03323930120123
Ce 10.93829864660257 6.87875355121596 4.10205126044675
Ce 8.42367504730458 9.51024010747954 4.03302703838853
Ce 13.53790804261562 9.43107269663527 4.05720206959685
O 12.40311418849132 8.07706832020656 16.11824866858274
O 12.43821808584743 10.87563432295579 16.11809069578900
O 9.59155104149355 8.12170582301605 16.16384782149153
O 9.64657367956133 10.92767943967870 16.15423516129853
O 6.97175444118422 10.94770613089496 13.62814225439928
O 9.66575266763767 13.61996706358789 13.61420469592905
O 12.42569717848111 13.54358281116385 13.57099423899321
O 15.08662192642017 10.81861845389659 13.55187166135930
O 15.06882726159921 8.07821327249169 13.56691704491526
O 9.59869898345074 5.47241595834687 13.60614207522339
O 12.37273331782219 5.42993345668656 13.60824551447616
O 6.89847013122233 8.19397153673443 13.64852276509784
O 9.62947437168929 8.18572327837111 13.56372408045197
O 12.31836828749730 8.11637799860189 13.49672924414966
O 12.35967006601470 10.81205259014621 13.50296850849559
O 9.67314010586518 10.88155424461978 13.53878135597960
O 12.53269776543180 16.14955450613528 10.94716567577542
O 9.64277480440931 16.18187254289766 10.95198644475801
O 12.49904395518894 2.96407995886660 11.07944533673071
O 9.33891239916257 2.97231235207223 11.09478792471209
O 6.88542692889329 5.48725618572396 10.87043824807111

O	6.95328946162382	13.66987234590995	10.91328226495133
O	15.12809552707569	13.51093130623547	10.87136825592445
O	4.35726117287551	11.03067171439017	10.90389364889763
O	17.63018767149836	8.00189032057439	10.91045840622599
O	15.03427916450793	5.39664253544714	10.87699474551090
O	17.65158619789461	10.81163590951544	10.89524183212884
O	6.95045941672635	10.90936440398832	10.93995657630362
O	4.31064765317505	8.21523739104470	10.93252533924631
O	9.67403688483177	8.07870079023816	10.97833515540997
O	12.36443826397956	8.14507900713384	10.85933383645881
O	12.36982158063358	10.86571260309656	10.86199714193100
O	15.01537713440621	10.78935715403273	10.83685791149047
O	9.70220507194193	13.53861476532042	10.89018503291582
O	14.98530282661667	8.09914021165399	10.83227480606625
O	12.41115897746801	13.50124361474724	10.85576389252215
O	9.60383613870450	5.48337720091326	10.81578487800354
O	12.25555452759485	5.50576826749498	10.83304747994192
O	9.67630591331356	10.91206405782836	10.95467832681912
O	6.95015504773167	8.16246719151521	10.93471226821032
O	9.66910796616155	10.91470253120367	8.06724459676227
O	6.96361981081522	8.16859945378232	8.06273146836660
O	12.40339499541317	13.49580066715918	8.14408647186131
O	9.69492639997791	13.53160367596410	8.12542240227610
O	9.60645977525370	5.49549833592135	8.17517873670874
O	15.00134207880366	10.79967832462518	8.14228254405392
O	12.25950845570539	5.51870242483111	8.15913447864358
O	12.36303973378968	10.87150507413533	8.14809386222745
O	14.97342977618381	8.10871787600059	8.14571209542913
O	9.67587886379443	8.07851712424701	8.03335333798169
O	12.36145990222300	8.15167354685073	8.14724992418545
O	6.94962106287243	10.91593702122159	8.08615932055523
O	17.65124374848952	10.84498672113380	8.08908332888995
O	15.13519537279735	13.53535389789352	8.13692480519376
O	4.31483753613342	8.22402411179103	8.07053652878056
O	6.93063778278474	13.68631952892732	8.13898790722075
O	17.62169025851033	7.99668578625174	8.05858949499932
O	4.33819692940425	11.08528643119899	8.12914443617237
O	15.04909409525805	5.39563446049453	8.10345149941504
O	6.87157789227415	5.48337405966322	8.12037469512173
O	9.63165031165136	16.17937788996004	8.06521686945121
O	12.52893622397000	16.14875358928937	8.05640617808953
O	12.49947003289182	2.98165589739714	7.89805897554800
O	9.32625797610255	3.00040303100340	7.88034208803159
O	9.67257784961967	10.88079440938882	5.47513343170702
O	12.33280372189174	10.80746335569214	5.50038978764248
O	12.29517162226531	8.13937276756947	5.50961381025700
O	9.63768060628791	8.22039844103072	5.44845187419476

O   6.87488634915167   8.18983589781502   5.37194996114416
O   15.09659327315556   10.85183857174260   5.44229607682243
O   12.42169506724399   13.58089399103188   5.44893471807923
O   15.08046757859328   8.08057667392336   5.42923961222501
O   9.62852175514763   13.66165133769227   5.42097758798623
O   9.59692547157545   5.47792779367541   5.42339417727212
O   12.36968743919220   5.41353416054290   5.42180383562146
O   6.93539900698421   10.96943540770566   5.41212164809622
O   9.44957561458879   11.11653817399443   2.93711904121962
O   12.60149294973910   11.06444275668263   2.96504883498953
O   9.38087466552685   7.92459090451947   2.95214681455168
O   12.56599344701766   7.88577415990268   2.98163648890803
Pt^{2+} 11.08109552796835   16.27246140131070   9.49935299777431
Pt^{2+} 8.32647602071162   10.81217485440145   9.51012785820156
Sn^{2+} 11.00426936921216   9.45887100761931   17.26698670513345

$Sn^{2+}Pt^{2+}/Pt^{4+}(Ce^{3+})_4(Ce^{4+})_{35}O_{80}$ E = -957.90231

Ce^{3+} 18.72992443242194   9.34445180225172   9.49923215843399
Ce   3.40710194450649   9.62724733488833   9.56954974779481
Ce   13.58617230515367   9.47346891936316   14.91127714992375
Ce   8.50182331212882   9.46574754547593   14.99658033841225
Ce   11.02405695875898   6.86581229095239   14.93912778989204
Ce   11.04222116015043   12.07091575500916   14.89248487361485
Ce   8.32442915214181   12.24694996084341   12.23204805118995
Ce   8.35299974393875   6.74024623039999   12.29944279145813
Ce   13.69646616785797   6.71575794478612   12.28786547487610
Ce   13.72523724192103   12.18953457255955   12.25570749974709
Ce   16.25276415236358   9.43398368923645   12.19148977090804
Ce   5.92127046725254   9.56436127509799   12.13955507784184
Ce   10.99438988335643   9.43180939054947   12.15788189607638
Ce   11.03225723008173   14.75842628633885   12.18188537905925
Ce   10.98362656595724   4.12105860453728   12.13439444493793
Ce   13.64254743966007   14.73935098818411   9.50115377225316
Ce   8.37467416894425   14.86573959141843   9.47891162746597
Ce   5.74611486950018   12.37293136092839   9.41638259205536
Ce^{3+} 16.32409224076783   12.14784186861136   9.50694614673327
Ce   10.92233472064871   12.11199498226499   9.48365822011069
Ce   13.60922475841253   9.47953939869943   9.51979400477686
Ce   10.94079621263708   6.82834210648734   9.56032981765889
Ce   16.22323531386568   6.75771179766095   9.50718021383346
Ce   5.87417762266832   6.98993463738331   9.48057711522902
Ce   13.58512279504706   4.14090771097322   9.48543890086930
Ce^{3+} 8.30076107564136   4.12266368722039   9.47283045222627
Ce   11.01525060598715   14.77360642416000   6.80198939851811
Ce   10.94041585707393   4.23632090306142   6.83797385481777

Ce 10.93770949354100 9.52640277438996 6.90434391563869
Ce^{3+} 5.65800151566960 9.64279115116299 6.60999749982666
Ce 16.22352641500143 9.45476608946383 6.82953452628086
Ce 13.72330989044176 12.22395148613793 6.73760053603846
Ce 8.21993148862360 12.32942086830585 6.58475669767469
Ce 13.65868572691536 6.76594693677531 6.75612608324251
Ce 8.24842944797639 6.86536281509544 6.69598519228661
Ce 11.06274244934800 12.11101622069905 4.07919788932867
Ce 10.99865558303722 6.93506355195273 4.11995452350627
Ce 8.45844196967002 9.56785175238913 4.06788279418908
Ce 13.57808394959994 9.49957787146228 4.11888106727447
O 12.58992315983800 7.88598596697237 16.01636755420446
O 12.59952517697501 11.08486760186412 15.99676077270559
O 9.41038349915437 7.89399651614345 16.08833194059659
O 9.45232397637310 11.07701709339382 16.01008388247488
O 6.90054755350684 11.00610855527625 13.42752190610721
O 9.61682551295250 13.59648822824046 13.52626772213878
O 12.39856154643262 13.56698097164113 13.56141132367501
O 15.09830347252828 10.84159987170126 13.57165094478910
O 15.09006119966731 8.06757504446736 13.57051882790842
O 9.62683847743395 5.40010203895511 13.59074742830186
O 12.39796307978362 5.37035002002738 13.56536381444823
O 6.92249192983245 8.17802447654082 13.50984280040871
O 9.69408934546601 8.18207971255565 13.61620802117621
O 12.33637089394130 8.12138521341508 13.49803712655356
O 12.34106241835219 10.79194692547093 13.48295910390063
O 9.67536330463795 10.84968835635125 13.44176928715414
O 12.45213786817160 16.13131458545167 10.96626851869875
O 9.57909322069248 16.14632759916866 10.97010180599050
O 12.44121888759272 2.82703258721253 10.88599656635079
O 9.67910631429844 2.76166582512220 10.92144756555986
O 6.81984827196663 5.62310039393167 10.89980979463013
O 6.91934471367806 13.59497150069710 10.89460964214632
O 14.99430062468721 13.59898177837824 10.93472036320805
O 4.40826070769434 10.96996508043983 10.93091832358921
O 17.63787609808346 7.92483913978330 10.90699535610873
O 15.04937270516726 5.36959322283808 10.88180282653931
O 17.71336994838776 10.71331847005311 10.97821044541823
O 7.06146854284081 10.87142574773053 10.50122638873924
O 4.47046272875985 8.23688168501597 10.92784541936433
O 9.80224234057942 7.88811543176298 11.11363625926361
O 12.40990032888806 8.11118752417403 10.86248963863256
O 12.29917630873188 10.84938727943184 10.85236434409038
O 14.98626536844309 10.79350759901796 10.87838066466128
O 9.63909319321712 13.44357172618672 10.82942991152331
O 15.00137143017692 8.10904182847890 10.85492073232452
O 12.30310535999624 13.44750794350972 10.84366044145699

O	9.61144122662733	5.38025963897128	10.86408178676721
O	12.30940277013714	5.45514608222288	10.86137023622468
O	9.54643416659658	10.63056258472104	10.73283906962902
O	7.28581223705424	8.32743303608031	10.75060592490364
O	9.29833510723794	10.85118296461988	8.17071853799265
O	7.02449115936298	8.56101334043975	8.18717789127316
O	12.29880171774995	13.45574229885175	8.14716355553943
O	9.60825103111473	13.48259809380192	8.09686472065261
O	9.59741294581481	5.58021135566567	8.11830290179297
O	14.95606512260440	10.81078476348958	8.14646198265594
O	12.29820692457156	5.49578065595344	8.17155923984796
O	12.26055922510427	10.84953889829151	8.17710036264129
O	14.95046830036932	8.12185838670497	8.17909007089495
O	9.52194869582695	8.29584666664644	8.41153707591712
O	12.28013753489824	8.13187391597582	8.16862070203386
O	6.62998441542565	11.32151727939318	7.79712318381694
O	17.69829245865451	10.72277731728830	8.03485673438721
O	14.99997271630439	13.61484352183127	8.07526469474599
O	4.25330256138977	8.23501339158795	8.21304416187593
O	6.86185134413967	13.91230698915875	8.17513827812669
O	17.60159029474918	7.93881419614644	8.10021147124564
O	4.00423351135866	11.07370297447575	8.23930226349455
O	15.03566049185507	5.39121756640882	8.11523508222717
O	6.79875512824862	5.67893757216210	8.02972245549160
O	9.58570810517039	16.18284173489084	8.04949117122810
O	12.44946812619172	16.14032527412887	8.05620801389944
O	12.41685543898009	2.88057367740358	8.07739038691923
O	9.65436291116517	2.86233319739325	7.99884557771721
O	9.70196018049587	10.92680882850033	5.55658767384785
O	12.33120244621258	10.83434682494973	5.55161623890830
O	12.29851789057367	8.19429851336301	5.56055522438192
O	9.66787020871588	8.22949673367694	5.59110511259784
O	6.94157737082194	8.11646782197890	5.39339276824016
O	15.10658255871233	10.87443587646935	5.45710901371009
O	12.41528483677384	13.60975854965245	5.44383958621709
O	15.06641493728931	8.09047190391480	5.46827555965174
O	9.61980533479810	13.68708349830048	5.38804131554104
O	9.57112924178807	5.48311340024885	5.42361147241659
O	12.36552358925422	5.43089664919343	5.45387207831243
O	7.00691517144106	10.99406077394441	5.16803112899685
O	9.52309044252773	11.12534618707825	2.98050222554533
O	12.66922054472697	11.11988268728861	3.02250596406646
O	9.43241964589001	7.95147259752669	3.02973203338789
O	12.59304116561217	7.91661743256050	3.03049017097289
Pt^{2+}	11.02108002087652	16.26149878584556	9.50945900646862
Pt^{4+}	8.30893110929345	9.59160462914586	9.44156054769465
Sn'^{2+}	11.00216279996547	1.71620773033090	9.45226058034406

$Sn^{2+}Pt^{2+}/Pt^{2+}(Ce^{3+})_2(Ce^{4+})_{37}O_{80}$ E = -957.93405

Ce^{3+} 18.71376198462901 9.36118391246020 9.46653574192244
Ce^{3+} 3.27052078013669 9.63831110232726 9.52754764273257
Ce 13.56296709745595 9.44315378451947 14.92248262545445
Ce 8.44172813351815 9.51719653272764 14.96466010175092
Ce 10.96386141888006 6.89210362788052 14.91434963429430
Ce 11.03662169195387 12.05768594710700 14.93999428290125
Ce 8.29877173384474 12.28436619668436 12.36576372805476
Ce 8.27697655537129 6.83382100084230 12.23571954171577
Ce 13.64667110304514 6.70904589054720 12.24933680633474
Ce 13.73259887380889 12.15824489193171 12.27961025119082
Ce 16.22577025718011 9.39259236475704 12.17882850523616
Ce 5.78680544855772 9.49528496295559 12.23030034243108
Ce 10.96536849267980 9.44836722079842 12.14402732890936
Ce 11.06365420437119 14.77354080686345 12.20173543551115
Ce 10.91817693666492 4.20630938233791 12.12246856515724
Ce 13.71795961945212 14.76829727770454 9.50364119183841
Ce 8.40950436718847 14.78982570064549 9.51798333270176
Ce 5.72543641825278 12.26652998023976 9.52466132037391
Ce 16.27586884854359 12.07755314222037 9.49740074402162
Ce 11.04904741726021 12.17057432596725 9.50552926778590
Ce 13.60349884465646 9.45295896147850 9.48587717810534
Ce 10.87160970082977 6.91529382443941 9.48166324123549
Ce 16.18964843183485 6.73573259679704 9.45967826938638
Ce 5.80517748835409 6.97928915286764 9.51106478389848
Ce 13.49849921069293 4.16142611239513 9.45785909137458
Ce 8.28008806082477 4.20815081641349 9.47268008340140
Ce 11.05097369374662 14.78711262131386 6.81807719350435
Ce 10.90621391640984 4.23336742191426 6.80449705906810
Ce 10.95364467115555 9.47178957722060 6.83836685687841
Ce 5.76382009398386 9.51331323785302 6.80692881092100
Ce 16.20791036498589 9.42328466121578 6.77528801717052
Ce 13.71509315533346 12.17927071225721 6.71216840761843
Ce 8.28632561896509 12.29908648571200 6.66130404461706
Ce 13.63213164169240 6.73506083166585 6.68935433940696
Ce 8.25742490985492 6.86096776779363 6.75342718583965
Ce 11.01260813087032 12.08862481256756 4.06123813644349
Ce 10.91963412635084 6.94035791439790 4.04161153805056
Ce 8.40159425033247 9.55546138986210 4.04248783626436
Ce 13.53524688670707 9.47587198936258 4.04251838836904
O 12.58532319381556 7.87313903906092 16.00137020010811
O 12.63120092583830 11.05259029463476 16.01195597485864
O 9.42555508049323 7.90511447694789 16.04535109393154
O 9.46784305517616 11.09994511139324 16.05412168290099
O 6.94860093333793 10.94600638690994 13.61795012787085
O 9.65150507850275 13.63312925595114 13.59879768040850

O	12.43834074041481	13.55226782950592	13.55301278847471
O	15.12324053816424	10.81313094992461	13.53424396487033
O	15.08918337027879	8.03937563796768	13.52254910016481
O	9.61066071224901	5.42684352774119	13.53507078260292
O	12.37422630564070	5.36247949843964	13.51685007593536
O	6.90016828879768	8.17313122458073	13.62801146157983
O	9.65448651673569	8.19843008293551	13.54860442167202
O	12.31359060292092	8.11697548250876	13.47745038214082
O	12.35117243296973	10.78758387314553	13.48560449458144
O	9.69070085784487	10.85827627287542	13.52595291097805
O	12.54637260404341	16.13378903161742	10.95401198844385
O	9.64946426896585	16.16681752380686	10.96025629963013
O	12.30714643594304	2.86365974694631	10.85117541339016
O	9.51024851044379	2.86263965170976	10.86803837330503
O	6.89051526347064	5.46398586155841	10.85752167680330
O	6.94907909453120	13.66817300590233	10.90630382212468
O	15.14732359493428	13.49867293806301	10.87118249853171
O	4.36117088766856	11.06032450539914	10.92018892670619
O	17.62360076696957	7.95819161150610	10.87525957768010
O	15.00925999623532	5.35920153491955	10.82757529106967
O	17.65854966415626	10.79621494642736	10.89243733568224
O	6.96408956917560	10.90297413345469	10.94397893229239
O	4.32638902686378	8.21758281212916	10.93893821285914
O	9.67852867004421	8.05572380360777	10.95999475530875
O	12.36158060978324	8.12603097110997	10.83628467715783
O	12.37590893922033	10.84733466015623	10.84642590668164
O	15.01130006294214	10.77310083254296	10.83569807612544
O	9.70780588816821	13.51749923101833	10.89330093160499
O	14.98340066814196	8.08267228290799	10.81458078010816
O	12.41881258053338	13.47896352634011	10.85889609195092
O	9.58537221338902	5.46775905438184	10.81325633336753
O	12.26660047834589	5.49176598233389	10.81599295711486
O	9.68282662883012	10.89733931374631	10.94562592418323
O	6.96020301551494	8.16394410487514	10.94513777464380
O	9.67384039689627	10.90382087881161	8.06534456193677
O	6.94854806363714	8.17306941455472	8.07087061725212
O	12.41041902227540	13.48650326405535	8.15007302470332
O	9.70130836419451	13.52433081322683	8.13188230413324
O	9.57908166505912	5.48144891894276	8.14320704115702
O	15.00703476652931	10.78780842829834	8.14219156482719
O	12.25983482820688	5.50512625985014	8.12099395679162
O	12.36969596141267	10.85929429673903	8.13796901287930
O	14.97670050266177	8.09883824043748	8.12783471443843
O	9.67080816786019	8.07143731155975	8.02004576857292
O	12.35662600822323	8.13791115936541	8.12741477504317
O	6.95405603231127	10.91092159491238	8.08724292880597
O	17.65389804641073	10.81071073666035	8.08121494350329

O   15.13754341127037   13.51351462065147   8.13757422961407
O   4.31491546221440   8.22758996621970   8.09796167761228
O   6.94433055175629   13.67383774243860   8.13550790629927
O   17.61829140128234   7.97426805432869   8.05570221583926
O   4.35056290033804   11.06618108987484   8.13044736504214
O   15.00445373569780   5.37458660411996   8.08556590188018
O   6.88274995824799   5.47469280051610   8.12997337266840
O   9.64163400273884   16.17425314423159   8.07484644911007
O   12.54108448351414   16.14168085410859   8.06720073086253
O   12.29956068572917   2.87620445682314   8.05429745213714
O   9.50055829146347   2.87719218818612   8.05565447281220
O   9.66985169188946   10.88700481949160   5.48052317476559
O   12.33205369344276   10.81454422975615   5.49715282203771
O   12.29001008003613   8.14822405166357   5.48239338791429
O   9.62884984138944   8.23273159926218   5.43289778878868
O   6.87165137503240   8.20143265174752   5.38682620437810
O   15.10228876472497   10.84875776776800   5.44007502706150
O   12.42512565670511   13.57919120262395   5.45445333259519
O   15.06797159506016   8.07858894041368   5.41797287359831
O   9.63635175253611   13.65592905652362   5.42588185133184
O   9.58485664037221   5.46276861865151   5.42069238377989
O   12.35444388434587   5.39858310188524   5.41594498472442
O   6.92597254828438   10.97172933910853   5.41326166459273
O   9.43526350489966   11.13706725193703   2.95597518994598
O   12.60318201045856   11.09677515323569   2.97191558910428
O   9.36899777005002   7.95121181556958   2.93340728336518
O   12.53881984563169   7.92838234098911   2.95326390628190
Pt^{2+} 11.09198431604923   16.26014017554769   9.51381340579554
Pt^{2+} 8.33163719361522   10.80215150938087   9.51015147257534
Sn'^{2+} 10.86167770200543   1.74684849123944   9.44774332196587

$Sn^{2+}Sn^{4+}Pt^{2+}/Pt^{4+}(Ce^{3+})_8(Ce^{4+})_{31}O_{80}$ E = -964.46207

Ce^{3+} 18.75319986797973   9.36859531442615   9.50044541047436
Ce^{3+} 3.25013109545434   9.66033229982117   9.52725515065084
Ce   13.66647907041909   9.48195473581009   14.83501842364688
Ce^{3+} 8.34705057284410   9.53220296186681   14.90739958352089
Ce   11.00211166484855   6.86412488445185   14.88155480140495
Ce   11.03162490667939   12.11201683653304   14.81458916659773
Ce   8.27937537541654   12.25631215694679   12.17846840295983
Ce   8.29461522554277   6.73795832652776   12.28238808780668
Ce   13.71093491473770   6.67487921003894   12.27235516223901
Ce   13.72920994612638   12.19608458434530   12.22812932169268
Ce   16.27539139792336   9.43222264663556   12.17441079692799
Ce   5.85347367278211   9.59318690519441   12.06173576806695
Ce   10.97429895015811   9.40540070342431   12.17260917678484
Ce   11.01603874701669   14.76792293659030   12.15472530291657

Ce^{3+} 10.94862269260340 4.07174193677361 12.27655386003995
Ce 13.64735890492095 14.73592858173502 9.49024667275746
Ce 8.35703681794245 14.83833061073467 9.44814921265249
Ce 5.69204278121859 12.31647325572317 9.36580686821588
Ce^{3+} 16.33416355163380 12.15166652368976 9.50310503225966
Ce 10.92777883536943 12.08983443734822 9.48624756627541
Ce 13.60835769764603 9.45477395083266 9.53018793096691
Ce 10.96075460369002 6.73430374728553 9.59147140807307
Ce 16.24646320464300 6.76536632549364 9.50597995958819
Ce 5.77918039832774 7.07062770647626 9.45072994687967
Ce^{3+} 13.69188262147246 4.08069205288610 9.47641400612241
Ce^{3+} 8.13139199924535 4.21097578832424 9.43986631624853
Ce 11.02718966998482 14.75917279717642 6.79824448487306
Ce 10.89317523061607 4.30333031960623 6.72236406506187
Ce 10.94582053404043 9.49840821485639 6.92021519830874
Ce^{3+} 5.62711986779987 9.69089631857362 6.64861352590848
Ce 16.23730231997315 9.45874203253984 6.83935531292256
Ce 13.73578429995175 12.21009307692949 6.74548794539342
Ce 8.22636696522467 12.33600877966478 6.58364920233937
Ce 13.67719362293282 6.75842096713292 6.76787176004552
Ce 8.21257905738391 6.91916236048081 6.71551859676392
Ce 11.06899509165908 12.10110208570673 4.09173001324078
Ce 10.99536087116639 6.96890508622647 4.09536366444094
Ce 8.44335972051638 9.59295570973254 4.08526605225874
Ce 13.59177034979046 9.49964911403293 4.12666003473223
O 12.51439200465458 8.11516385460091 16.16770539067318
O 12.51455310797937 10.91529884613460 16.11617641671321
O 9.74382883192630 8.08979038283914 16.26587607613287
O 9.75420013418863 11.00113547772603 16.17577885087905
O 6.88356120162651 11.03993827192978 13.39789852109679
O 9.62755167346645 13.61651640119204 13.52897906422532
O 12.40319626339930 13.55803171425778 13.57146909443943
O 15.11398645701970 10.84469852910384 13.58730545105665
O 15.10459318650955 8.07762379234755 13.59088918616735
O 9.56458353230889 5.46688450924915 13.70548072149310
O 12.42310987655080 5.43777458205040 13.68196409664846
O 6.85229181297373 8.15991871244058 13.44513542170243
O 9.64691636155607 8.17025434864712 13.62862337925252
O 12.35634696232176 8.12819384905291 13.53556450102731
O 12.36633250900548 10.80858018746174 13.50730997464464
O 9.66715281451905 10.89804643168780 13.45425733414868
O 12.45659836540766 16.12607970131833 10.96220227745130
O 9.57964067063853 16.14096612936351 10.94680746244085
O 12.26940442338997 2.57402583375621 10.78015690021360
O 9.44454777981760 2.51916781371836 10.74219959206907
O 6.76655930699762 5.63454322439110 10.86916477318830
O 6.93393908678118 13.60592855478661 10.86658138169541

O	15.00137215967267	13.59464209076397	10.94333411641326
O	4.45240464909874	11.03333952816928	10.92308333819865
O	17.65650094095252	7.95837624992620	10.92079539841105
O	15.14011508738058	5.40567731953522	10.92462240397649
O	17.72587978816413	10.72930196193767	10.98477731272088
O	7.09278917568609	10.87396496501258	10.47659966507370
O	4.46137200132435	8.23379811465339	10.92675156993750
O	9.82714159496815	7.82095712659327	11.12665572651576
O	12.41149524558919	8.06628090901602	10.88631090978820
O	12.30280086983951	10.82005699149631	10.87663366603385
O	14.99465940163741	10.79237066259852	10.88344864635763
O	9.64732362967714	13.43375765932262	10.81826009621439
O	15.00890251469430	8.11473713358160	10.86546100753199
O	12.31123032547921	13.43833262036488	10.84651830079945
O	9.55432055034260	5.27304112973413	10.87927478357030
O	12.34674269456484	5.36712154825867	10.90946784570770
O	9.56537705023654	10.59754336457489	10.75209465973528
O	7.28414445256015	8.33073801228315	10.72495903603749
O	9.36059445149150	10.82739711238588	8.18305262983282
O	7.05279522704066	8.57375265185451	8.16777024107847
O	12.31423397063871	13.43572496956486	8.15527880279838
O	9.63324963427729	13.46642876203622	8.09215783877223
O	9.57962962758404	5.54704276054613	8.10123184898742
O	14.96744687998508	10.80907637490421	8.15997227704055
O	12.29693299475539	5.39628897687581	8.12824492880803
O	12.27779698150699	10.81672282701437	8.19345224213705
O	14.96238807804468	8.13802643361894	8.18633589467831
O	9.54844310421749	8.24899486157617	8.43372975465064
O	12.29240947266983	8.06757263300305	8.19085435315501
O	6.73158363524315	11.29291133151451	7.79898549090476
O	17.71598936968341	10.74223209031387	8.04194475327979
O	15.01696366828603	13.61933122564141	8.07940087269042
O	4.29341836948462	8.22106270026270	8.15151624303958
O	6.89574910343333	13.90665603046891	8.11653653059280
O	17.63023482674129	7.96199028269194	8.10558837353993
O	4.11087890418238	11.16694123056064	8.17160186507839
O	15.11024231356276	5.44904428934518	8.06264425225653
O	6.77259539169059	5.71454701338155	7.99976803212280
O	9.60488271454522	16.17106284669075	8.03141226022223
O	12.47219600289536	16.13014974553089	8.05166497256082
O	12.23177382709680	2.66642513938426	7.96128040701553
O	9.52280530762278	2.81918761590745	7.93865650965796
O	9.71690118937528	10.91417089476203	5.56315186225696
O	12.34665296276431	10.81854018528120	5.56612533930742
O	12.31085027370456	8.18113491936618	5.56581507972618
O	9.66965260971378	8.22768548455150	5.59530139626702
O	6.94720548888632	8.14912307795637	5.38798975929250

O 15.12784307198999 10.87285725483647 5.46631264642369
O 12.43938710934690 13.59221190069705 5.45083850147686
O 15.07722939545903 8.08736543451906 5.48002219214192
O 9.65145028623543 13.69032266989636 5.37260605038598
O 9.55881486529792 5.47971657406604 5.39554303895749
O 12.34143866865876 5.42619173782473 5.41426946225236
O 7.01573549379851 11.03019860524270 5.14639765568894
O 9.52820408874096 11.13871982310741 2.98572119315118
O 12.68811449967566 11.11154237917465 3.03060432556187
O 9.43561019012943 7.97195349033863 3.02385341411774
O 12.60396754138601 7.93015106789286 3.01934330722485
Pt^{2+} 11.02976907276880 16.25246681574482 9.49571961649036
Pt^{4+} 8.33429187619516 9.57333507073951 9.44123024266679
Sn^{2+} 11.10715180763189 9.50974521624359 17.30492968809241
Sn'^{4+} 10.86095113064944 2.18189973050583 9.37488631117579

$Sn^{2+}Sn^{2+}Pt^{2+}/Pt^{2+}(Ce^{3+})_4(Ce^{4+})_{35}O_{80}$ E = -964.88770

Ce^{3+} 18.74316232055596 9.29952173658644 9.47943137739971
Ce^{3+} 3.26275716904598 9.64285849189318 9.48878618076282
Ce 13.61028387769905 9.44630596169826 14.83694974843414
Ce 8.38429485800141 9.52267986570999 14.88643276488498
Ce 10.95412319268467 6.81960264048357 14.82468749290473
Ce^{3+} 11.05361413635300 12.16875676704254 14.91214910001486
Ce 8.29471196330738 12.32574998234209 12.32953828823817
Ce 8.25672675526714 6.84460735353925 12.19904586853385
Ce 13.66003829083959 6.73639696162348 12.22034694086172
Ce 13.75542342339420 12.23267517072592 12.23574560085048
Ce 16.26563692246044 9.43023127604879 12.16897264973624
Ce 5.75538959340940 9.52058760238555 12.19424682855625
Ce 10.97280429468391 9.49403058872083 12.16239300345550
Ce 11.05191264144867 14.81704044975050 12.15329133436373
Ce 10.90690001673361 4.20012492758471 12.10427651706037
Ce 13.70973406551337 14.78300097559692 9.46991806123503
Ce 8.39671625684818 14.81833718194012 9.47577260410052
Ce 5.72773097849708 12.27735491162515 9.49762097645571
Ce^{3+} 16.37114974011102 12.14784701277678 9.48093406789578
Ce 11.04241279735497 12.18869311243238 9.48791199113778
Ce 13.64010348531393 9.50613642924683 9.49693782992412
Ce 10.88221777027604 6.94542519483073 9.48911886545759
Ce 16.20943078051210 6.74406559736932 9.47107805284833
Ce 5.79451807376709 6.99098283879903 9.47749869021517
Ce 13.50325826978812 4.18097526317657 9.47063992629522
Ce 8.27644048175389 4.22185114363326 9.45916422677433
Ce 11.04759229761195 14.79622309008895 6.78523880065127
Ce 10.91326828219661 4.24813568859189 6.81127843713763
Ce 10.97044814951089 9.48431631604715 6.84764428239641

Ce 5.78371313301374 9.52512981272654 6.78009710866527
Ce 16.24008395618188 9.43940297755898 6.79728024856942
Ce 13.74689063476060 12.21151443721097 6.72204972599407
Ce 8.29508726206283 12.30797302056250 6.63963883875885
Ce 13.64767364877805 6.75712296943212 6.71575538935046
Ce 8.27021630283592 6.86770350893642 6.74503494108004
Ce 11.04133357875440 12.08757816356364 4.06377385740717
Ce 10.95309050683684 6.93785795192172 4.05719900866461
Ce 8.43488430533013 9.55324733426128 4.02623756270857
Ce 13.56601305308871 9.48306512059416 4.07536329808316
O 12.39119925998759 7.95920888344818 16.12236149890145
O 12.49435097689956 10.71936235520751 16.21227288742759
O 9.58754250572094 7.98135448643657 16.17178595523938
O 9.58116493073111 10.74862738906136 16.24359971580082
O 6.92119678689514 10.93693781174812 13.62680884544224
O 9.60065190414327 13.69089937802989 13.54233785038790
O 12.46795286416220 13.63748617997671 13.50404698053970
O 15.12943866495602 10.80475570581385 13.58815101886647
O 15.04519488466495 8.04435357987227 13.55766774100193
O 9.58104397479503 5.42876325043862 13.54826468511182
O 12.33684152808374 5.38587314337933 13.54381747937192
O 6.89819406144555 8.17868968999566 13.62660430258360
O 9.63185255079296 8.18457796167578 13.57658989129411
O 12.30930531020328 8.11823911985353 13.51056507570118
O 12.39060136671207 10.82674600091284 13.52405338035837
O 9.63774340353596 10.88649001697585 13.56428845679194
O 12.50105947635704 16.17041680455227 10.91487954897095
O 9.61290202796410 16.19674498018777 10.91454985305722
O 12.29859802144120 2.87688099203430 10.87802114649605
O 9.50425973779699 2.87573605039816 10.87795112431336
O 6.88328654338515 5.48029657208737 10.84591081813574
O 6.92673277513416 13.67229982426969 10.88467881675207
O 15.05499260513279 13.60661505668923 10.91167213153306
O 4.35318776699138 11.04859424306561 10.90519371666261
O 17.61624887797726 7.89735368533411 10.89135388207147
O 14.98128542183649 5.36717542345089 10.86573318599180
O 17.74592833390625 10.67901864277246 10.97363809910692
O 6.95843148667550 10.91265192791970 10.93951584843126
O 4.31211561663746 8.23008913885326 10.91414075997976
O 9.68631017080688 8.08378664051502 10.98929127770398
O 12.36548197864793 8.16225012746330 10.86678127420863
O 12.37249151684408 10.87382015319426 10.86655785874115
O 15.03673746662361 10.80441778368477 10.87392053062805
O 9.67889582408327 13.53873984115338 10.85457154251287
O 14.99035161717927 8.10290962319391 10.83441743021551
O 12.37503187494242 13.50011102697996 10.82388675718214
O 9.57774227102966 5.48482152031773 10.81576829242628

O	12.25402965562414	5.51093997845751	10.83067566975241
O	9.67901164301206	10.90910430521662	10.95865400630753
O	6.94339503053411	8.17636505923637	10.93106916510482
O	9.67021080441394	10.90670547819864	8.06320223773503
O	6.95212627585482	8.17761404233383	8.06052749497486
O	12.36909643843367	13.49600496931411	8.12226688821387
O	9.67652491612959	13.53421019623670	8.10258207940259
O	9.58247196533908	5.49256278711974	8.15156147737873
O	15.02648452579765	10.80619387123823	8.12524014195863
O	12.25859167632092	5.52152866886930	8.13990618246264
O	12.37753787993199	10.87488996751437	8.15376647253441
O	14.98529994122179	8.11263467422268	8.15058121127578
O	9.67658854651468	8.07942506011139	8.03055052204090
O	12.36620016797546	8.16043885635491	8.15580556070807
O	6.96367879364521	10.91168305414021	8.07968007418338
O	17.74307852431801	10.68796925360943	8.01096059569668
O	15.05414962425074	13.61467658591376	8.05725101444870
O	4.31359853112500	8.23108090366041	8.07567783715375
O	6.91969507925475	13.67433128547445	8.11153355330338
O	17.61857587310715	7.88940968663175	8.08570182938798
O	4.35293607534466	11.07242582038726	8.10830089415633
O	14.99423149552476	5.36689037769161	8.11197366630404
O	6.87762503222679	5.47816808689199	8.11846449467271
O	9.60495143965939	16.18600601588556	8.02707431041087
O	12.49699753781874	16.16286674942885	8.01585678139388
O	12.29605080393890	2.88634660583603	8.08103427324284
O	9.49261188027532	2.88350486870401	8.06758603767806
O	9.68150259831787	10.88612964190217	5.47541167962113
O	12.34634916204722	10.81970198925411	5.50828239898580
O	12.29885760616129	8.15242628708094	5.50692914948948
O	9.64414107635282	8.23105559629675	5.43935616125431
O	6.88515253105269	8.20035754840682	5.37898570745347
O	15.11741334264805	10.84672364808242	5.42692656529161
O	12.41688815665269	13.58473386467685	5.42218758200277
O	15.06129892726785	8.07181199484151	5.43699391668267
O	9.62710104193962	13.64956920860742	5.39600598397607
O	9.58825961455053	5.45711093061422	5.43207171414423
O	12.35471284011580	5.39875598522758	5.43845430063637
O	6.92827484392122	10.97349672468409	5.40049570519512
O	9.45360704513407	11.12224288155014	2.94244451655348
O	12.61852936186352	11.08575205293318	2.97489892169846
O	9.39842528675962	7.93903640389626	2.93396748618917
O	12.57001938653851	7.91177472553705	2.98075901465085
Pt^{2+}	11.05673131917611	16.28481577027592	9.46644301340379
Pt^{2+}	8.33292621906918	10.80534225372005	9.51061629512475
Sn^{2+}	11.01333817871101	9.36774701126729	17.30882204952028
Sn'^{2+}	10.86245316723024	1.75536419644499	9.45110673430506

Data for structures displayed in Figure 13

Reference to calculate oxygen vacancy formation energy: E(½ O_2) = -4.9118

$Pt^{2+}/(Ce^{3+})_2(Ce^{4+})_{38}O_{80}$ E = -959.88663

Ce^{3+} 18.69230807884792 9.34353840249128 9.49959493117603
Ce^{3+} 3.28437873823193 9.58997825518872 9.51222709300141
Ce 13.54653720289663 9.42351258796489 14.93904391872757
Ce 8.43696085780908 9.49599295041025 14.93536189720982
Ce 10.96069417894104 6.86212354494084 14.87416819702327
Ce 11.02401473072252 12.06068212806118 14.94667989387315
Ce 8.32185646476723 12.25274424182645 12.29454351141144
Ce 8.27101815672055 6.75126123861083 12.26486641863472
Ce 13.63370475926452 6.67385919584450 12.26270556360933
Ce 13.73993932038969 12.16922125315632 12.29406168543000
Ce 16.20591521511011 9.37897998718549 12.20042059235656
Ce 5.77644049682846 9.53616773877847 12.21002764421436
Ce 10.99222858159013 9.49203696215681 12.16114439335769
Ce 11.06234803317932 14.76825475815808 12.21063239798405
Ce 10.91537179923760 4.09289652256264 12.09774669458044
Ce 13.71412335759627 14.75839925802769 9.51561780529829
Ce 8.41531307924639 14.83810111332960 9.51341322610369
Ce 5.77146667500996 12.22892395429124 9.51100770779089
Ce 16.28133756782175 12.06639540870088 9.51194758362196
Ce 11.02652429921525 12.16114632884589 9.51078951837688
Ce 13.60189094506685 9.44986476772047 9.50315204742778
Ce 10.95296692672804 6.84512491549792 9.49740706132392
Ce 16.14854329629916 6.70164340361159 9.49266887007699
Ce 5.74824255255522 6.85882033485971 9.50398987724624
Ce 13.46021618437783 4.02798019319752 9.49078510285024
Ce 8.37013101332809 4.10332233567327 9.48588102529894
Ce 11.06552282632468 14.77932280754454 6.81780695693820
Ce 10.91278831104991 4.12286767945533 6.87553667676194
Ce 10.98928166213042 9.50529767638118 6.85194863491982
Ce 5.76638939162204 9.54841022412832 6.80611639256075
Ce 16.20168750713686 9.40676560736465 6.80170264225585
Ce 13.73702819693515 12.18844240917782 6.72397025523792
Ce 8.31919749869375 12.26284039754853 6.72829510590187
Ce 13.63378517133614 6.68991403485939 6.72671297189458
Ce 8.25957954899344 6.77241811953158 6.73453670691484
Ce 11.02489031389699 12.08115999157093 4.07446683181214
Ce 10.93745920763469 6.89295488864125 4.12051857530247
Ce 8.42709330410116 9.52513394227069 4.07534488976285
Ce 13.54252760158868 9.44466250520917 4.06824319445915
Ce 8.37138910178196 9.53091947002640 9.50898711636468
O 12.57215539459184 7.85590251889920 15.99845499675129
O 12.58623146229027 11.04571688301390 16.02428896256881

O 9.39675782663256 7.91961483620364 16.00211131710499
O 9.42948807740117 11.09993410655032 16.02126160244452
O 6.92587443875533 10.93162920474580 13.55766746044513
O 9.64647097999142 13.61580598897219 13.56576121180936
O 12.43165435081620 13.57146140092153 13.56702353040282
O 15.09855367017716 10.81579646067515 13.55331768478170
O 15.07375624771642 8.04003683125299 13.55280047199027
O 9.54203626882483 5.44880720955616 13.56436967242582
O 12.33386637248505 5.40951702727507 13.56272767736888
O 6.87430495917109 8.16024860630701 13.56209225505451
O 9.64585822622197 8.18128198391260 13.48158772211078
O 12.30207579966865 8.14525398300460 13.48245189747651
O 12.34248965185810 10.80887769577179 13.49754219740660
O 9.68134930875513 10.84849370754715 13.49670486329156
O 12.53177996911668 16.13118057355823 10.95921356470028
O 9.63669737718716 16.17299150292634 10.95811706231153
O 12.48766958586813 2.96745603389282 11.08878616663370
O 9.32116854605605 3.01306446310153 11.08004201541301
O 6.84270916053393 5.50310385437607 10.88394317757571
O 6.96241106645506 13.63271534491878 10.88035134736717
O 15.13267606213408 13.50714486733284 10.88479059118783
O 4.37466760339143 11.00693099767418 10.91159443353281
O 17.59991193864570 7.97095855903241 10.91611999004146
O 15.02156643495416 5.37924690410846 10.87804978701733
O 17.64320964828274 10.80190885100522 10.90644407576164
O 7.01592017264009 10.90140767490976 10.85575738030250
O 4.34201752215359 8.17397139873112 10.92871895583848
O 9.61287307655358 8.17255508144499 10.85801130470573
O 12.33008245338278 8.13180975703943 10.85718078866594
O 12.36900461708099 10.85422620891775 10.86076923306275
O 15.00022776023390 10.77814976122168 10.85232990966854
O 9.68925306680598 13.51735020179189 10.86970411316370
O 14.95053875458495 8.08843168222031 10.84002141064438
O 12.40441317556314 13.47599550552402 10.87130926829961
O 9.60718319847045 5.53543749164255 10.82446969547768
O 12.25914318072602 5.49568004447816 10.82436390347561
O 9.64920727783855 10.89536959602883 10.86320677997538
O 6.99179376673438 8.20986663082382 10.84907693905381
O 9.64758509528698 10.90160053116607 8.15520254092581
O 6.98535240684080 8.21834568431576 8.16035608198515
O 12.40236277185912 13.48125522324928 8.15570371974127
O 9.68815684442177 13.52254872360899 8.15773022341821
O 9.60817711081627 5.54746735906732 8.15957479581615
O 14.99730270877364 10.78968533259661 8.16081721099750
O 12.26546208042308 5.51035373379645 8.15907511703980
O 12.36672810939330 10.86117145864559 8.15414884323671
O 14.94903137476520 8.09864808907115 8.15203018837885

O 9.60903972541991 8.18022507506154 8.14706920191539
O 12.32796794999937 8.14009527168310 8.14353993040469
O 7.01366446468013 10.90715004487522 8.16370335498294
O 17.64031817628135 10.81682995333454 8.10691125513321
O 15.12960544875244 13.51860219409784 8.14597925089506
O 4.33558811242795 8.17934895216604 8.08598173275818
O 6.95971617202509 13.63873490779235 8.14312923341171
O 17.59928120225102 7.98709171473017 8.07229508948859
O 4.37116163212197 11.01249462108901 8.10965362703426
O 15.02669415317670 5.38745074738178 8.10071594408369
O 6.84093919764137 5.51245947340529 8.10902398008420
O 9.63759725935255 16.17759421135117 8.07016343417841
O 12.53495296529662 16.13628091047055 8.07375128118995
O 12.47320187440882 2.98352337838646 7.89484265445392
O 9.31308250938244 3.02846819381874 7.88734092385061
O 9.67715283631553 10.86779613972271 5.51865159315633
O 12.34060766829183 10.82701191235798 5.51716890998769
O 12.29483042050502 8.16222388742148 5.51925599773612
O 9.63637188701862 8.20620084463184 5.52092100922580
O 6.86094491537699 8.18026422685456 5.44708011040253
O 15.09973185798751 10.84858217766746 5.45896997045456
O 12.43677956874574 13.59215040280420 5.45808253609278
O 15.06606694147305 8.07198892817729 5.43680217712146
O 9.65186077652992 13.62993909226892 5.46096398466831
O 9.52866215425089 5.47514648753682 5.42193307947776
O 12.32130750333714 5.43123304108841 5.42168055019186
O 6.91895339488537 10.95096364770997 5.46024963615362
O 9.43427401777762 11.12022410043377 2.99285407784250
O 12.58894646355920 11.07764112966982 2.99196919487223
O 9.36075684079810 7.93914333704917 3.00096927395133
O 12.54515663252630 7.89754734876082 2.99838476272461
Pt 11.08678669836029 16.26258454457044 9.51521649413476

$Pt^{2+}/(Ce^{3+})_4(Ce^{4+})_{36}O_{79}$ E = -953.11330

Ce^{3+} 18.71668721971986 9.24743861212067 9.50117576939244
Ce^{3+} 3.28002006237020 9.45323587226229 9.51587176773142
Ce 13.58535129712260 9.45363930368596 14.94376784181987
Ce 8.42022681317536 9.55096431691712 14.84019002886117
Ce 11.00090310235979 6.87920915189343 14.81550407042032
Ce 11.03480670579441 12.09297593367748 14.94836717942816
Ce 8.29552837394310 12.30440559229559 12.27265519782816
Ce 8.08680564926875 6.57608476951802 12.46422302791933
Ce 13.67667353279912 6.71074326607047 12.24738345147157
Ce 13.79334712970526 12.22242285447005 12.28053010219782
Ce 16.23657721961722 9.39505731304017 12.18472915405123
Ce 5.79930907548898 9.52976125795516 12.17423711445224

Ce 11.07489486750450 9.58816744496511 12.20157224637370
Ce 11.06456706221388 14.77224566745393 12.20384587833637
Ce 10.94724824009640 4.18162100217181 12.11447906139158
Ce 13.71861983917410 14.75956804121502 9.51604141051859
Ce 8.39428444978791 14.82662839648091 9.50899047017370
Ce^{3+} 5.63579281736447 12.29750840781084 9.50439637601167
Ce^{3+} 16.37829507877890 12.11168160978436 9.50189380502758
Ce 11.03259972068279 12.16839484821462 9.50185825642079
Ce 13.63886379846792 9.49414704183967 9.49230074836573
Ce 11.03033303566933 6.81116173409323 9.42549635535186
Ce 16.15903035767719 6.68762197452989 9.49012000993627
Ce 5.81018322871688 6.85940502719951 9.50794761475195
Ce 13.47423869566342 4.01605814435452 9.48901747276013
Ce 8.35980013594998 4.18594109694853 9.46767674842197
Ce 11.05997409373487 14.79114517858633 6.83362325328381
Ce 10.91246887462461 4.11246601974152 6.86282911032738
Ce 10.99189563040952 9.52762191117719 6.87427638460180
Ce 5.71175444066685 9.53956736545286 6.77763762626182
Ce 16.23749634162523 9.39659674795473 6.81613946265802
Ce 13.76578343553585 12.21429795651422 6.73393713957463
Ce 8.28143862772275 12.31988892952508 6.70977837666131
Ce 13.66671814271698 6.69836763968148 6.70840417910594
Ce 8.24429542758456 6.76725792539465 6.69349931145332
Ce 11.02437109948375 12.09457331985759 4.09919141465356
Ce 10.96897578666967 6.91192176022535 4.13057771550109
Ce 8.41704530374018 9.55889127233175 4.10362330781430
Ce 13.55636747599642 9.46752980216352 4.09601955479193
Ce 8.26094581923632 9.66944430604494 9.41241438252741
O 12.58367483750129 7.85000244888503 15.97253830496236
O 12.58281662498169 11.04962897183146 16.03336162165569
O 9.41498665554246 7.94491255032255 15.81849075637373
O 9.40993281851858 11.09812518477896 15.98702489690026
O 6.94265201560680 10.91731116544909 13.55600785181199
O 9.62863273298869 13.61613897599770 13.57019173594409
O 12.42245754284338 13.58313909571532 13.58766065887592
O 15.11843018150816 10.80733982609707 13.58411091933524
O 15.06387636123026 8.02141010669607 13.55012294571070
O 9.53247197722911 5.37807329000806 13.65665767767799
O 12.34152773385774 5.43933996277978 13.54788536995656
O 6.80874126880708 8.12326242546902 13.62325446872633
O 9.58634821638012 8.14360505161454 13.15187161254775
O 12.31680145656609 8.18585375777564 13.47138819581135
O 12.37067194724335 10.84140330346464 13.51553622184144
O 9.70840441523215 10.87630211022703 13.47615815785945
O 12.50893249344672 16.13649330167239 10.97684216233436
O 9.62730855647130 16.14193391528274 10.98040438067808
O 12.47219099446315 2.99712244891041 11.11155389144824

O	9.32934193809556	3.19517245983750	11.09050393970718
O	6.79046459102035	5.42248554437268	10.92459856846063
O	7.01765531665139	13.70341773622392	10.95674730696545
O	15.07069380615124	13.59020625287230	10.95193303012669
O	4.29210759451362	10.82465154181681	10.99646599386296
O	17.54906725401903	7.86980171688592	10.90673687867483
O	14.99885248680619	5.36842504228582	10.9025726860630l
O	17.73725891617629	10.64188372563958	10.98483170829319
O	6.98571824127316	10.92917856559834	10.84798151144331
O	4.49907299206729	8.06934156103009	10.92441420244415
O	12.16565772157308	8.18249377509570	10.82280664331774
O	12.40606464795799	10.89042870920502	10.87622629550002
O	15.02877346188298	10.78525851686893	10.87636013593298
O	9.69760333636390	13.44499228590605	10.86813353594900
O	14.88831683876209	8.09241292668055	10.84105963228689
O	12.38601998555409	13.47721038673989	10.87315444429078
O	9.55835942746531	5.85774305679060	10.89027099168565
O	12.27269272920319	5.51372283397164	10.80373605234740
O	9.66605655141847	10.71290319782433	10.82117337640359
O	7.26061334889208	8.16812139374816	10.88795701748308
O	9.60423094886585	10.95067622985621	8.12303757412442
O	6.99656975739483	8.23666356207868	8.12614917508555
O	12.38142869826437	13.47210384296603	8.16084746560381
O	9.69834396655173	13.52415014406188	8.15378000740220
O	9.62805161603153	5.57682798132519	8.14448948187670
O	15.01015933166669	10.78793860618752	8.13800622095006
O	12.27725504250298	5.48791714319848	8.14281708369791
O	12.35431643648377	10.85270656404728	8.17615616000598
O	14.96181292629234	8.09658462222231	8.15869338202066
O	9.65262962261744	8.25408928918955	8.39197524365415
O	12.36878798346843	8.12948582690900	8.11974538238750
O	6.93694584378003	10.93637802789569	8.10527912191726
O	17.72959340065780	10.64823821136109	8.03374480083400
O	15.06066598043911	13.59457717369259	8.08722087742539
O	4.36207350561483	8.03298507710909	8.12073413436800
O	7.01008168055707	13.72033030400342	8.08779100867122
O	17.59192465337982	7.86128771589660	8.09013197942202
O	4.24694256842955	10.82741735569626	8.03070851565637
O	15.00286598078328	5.35559106462360	8.10671790472793
O	6.87918924312180	5.49486543109027	8.10796345808915
O	9.62999146328743	16.18198103938186	8.07257825669842
O	12.50816245047718	16.14555317154184	8.07764845846972
O	12.44772646336989	2.96819805208892	7.90611034217298
O	9.28830174524569	3.06092965532769	7.90390462439131
O	9.67491202108607	10.86771640411626	5.52804882461731
O	12.33751351850759	10.83334522467164	5.54631628484876
O	12.28350263666806	8.17809229354905	5.52826033542318

O 9.62098936086534 8.19854934687941 5.62371176404757
O 6.84895409457177 8.14855511055857 5.42978640953416
O 15.10652344357634 10.82692947277370 5.44441893951628
O 12.42549524020494 13.59375519542897 5.45867218726785
O 15.05639055916928 8.04157433975908 5.44053684249943
O 9.63418876563192 13.63528493439496 5.44710331410215
O 9.51048555977568 5.46128362919249 5.42263784419558
O 12.31347663731042 5.42942013061591 5.42243995423578
O 6.89526847910832 10.92853550900158 5.42526216693890
O 9.41452455458949 11.13272406066873 3.00943306661374
O 12.58593417518516 11.08429570628983 3.01536065126785
O 9.39758413339814 7.96512805603444 3.05761866933825
O 12.57376392467871 7.88882845502934 3.01673806727569
Pt 11.06929585346386 16.25730672641988 9.52728503902575

$Pt^{2+}/(Ce^{3+})_6(Ce^{4+})_{34}O_{78}$ E = -945.92821

Ce^{3+} 18.69248555977089 9.34688894708679 9.50010210016056
Ce^{3+} 3.29955354750247 9.47940886852288 9.51106940879801
Ce 13.55093546360360 9.40274091731766 14.96666928379042
Ce 8.40424281538997 9.49468393942238 14.75671282853418
Ce 10.97118179681383 6.83007981579041 14.86899848326242
Ce 11.05240963099956 12.06207822703839 14.83679719603203
Ce^{3+} 8.04750348758492 12.55812195337760 12.55968793242402
Ce 8.08325319171562 6.52308171714365 12.49128099620479
Ce 13.69564202776506 6.64811704324751 12.28541813750698
Ce 13.78578722781382 12.15416512800831 12.27948176387118
Ce 16.21703676995520 9.36409669070365 12.19863850782204
Ce 5.78956201524633 9.50900583203274 12.20208350220025
Ce 11.05674040769086 9.42396131141250 12.16686103990661
Ce 11.09905920397510 14.70498807179003 12.19028428831085
Ce 10.93950621854247 4.11224857073788 12.14826867900101
Ce 13.77216536893187 14.79086980300166 9.51039323628991
Ce 8.42051718755424 14.76916273825734 9.52774470679953
Ce^{3+} 5.68052472615400 12.31586111503568 9.52731802761072
Ce 16.29162563377051 12.07110057095021 9.50237606471850
Ce 11.13324237388776 12.24115805932108 9.40643658239544
Ce 13.59818744583139 9.46900612009523 9.48212283540682
Ce 11.01082330843216 6.83146834198771 9.55672324114359
Ce 16.16248556195774 6.70126926597272 9.50147968958949
Ce 5.79321257040847 6.83529483958682 9.53180611553805
Ce 13.46505543722212 4.00866211330523 9.51167953178072
Ce 8.35509044205661 4.24666778153033 9.50838136686016
Ce 11.06217595977655 14.81923072965440 6.77606065309495
Ce 10.94216382592811 4.20457390763552 6.87664703947124
Ce 11.04111894652988 9.60744547706152 6.80352511735636

Ce   5.71548918338955   9.54859300090663   6.78553706868247
Ce   16.20146858684249   9.39196104427758   6.77953049810200
Ce^{3+} 13.84827840717064   12.28664408277011   6.61304893070935
Ce   8.28414324427354   12.33425490082483   6.71886753908521
Ce   13.67467795874964   6.70728187749972   6.73813729646443
Ce^{3+} 7.98006340277607   6.51544800339820   6.45663527901370
Ce   11.00335292195252   12.12687761716130   4.04942470303821
Ce   10.96065574240048   6.89011753507938   4.21356386911288
Ce   8.37451380044545   9.56447175270476   4.19391479205197
Ce   13.53133779696093   9.47475917311590   4.03275032444744
Ce   8.16819030396108   9.61687008675446   9.46100285639755
O   12.57486646564214   7.82170892284287   16.01783742200587
O   12.56933118864870   11.02277696318340   16.00045107087750
O   9.40777832850467   7.94532757110255   15.85476459913091
O   9.38458412025373   11.11594882340546   15.79488849737858
O   6.76628186148380   10.81864955513945   13.68892049859659
O   9.78485762357812   13.74288832409045   13.70494778925345
O   12.51760762154895   13.50067807467308   13.54857696280973
O   15.13037836018452   10.77911699461721   13.56084712078452
O   15.06766964508643   7.99811992338640   13.56477557311826
O   9.51114621727860   5.31835953636723   13.70148509987208
O   12.32752981156666   5.39119912082689   13.58432151580286
O   6.82272020176260   8.04881502168667   13.62794348860036
O   9.59097421092702   8.06441346900261   13.23975489650608
O   12.28827216140250   8.11812066344613   13.51620573354438
O   12.36935669424792   10.75482076477489   13.47333019207908
O   9.64153271484522   10.84529589374982   13.17565719776097
O   12.56700123445267   16.10411436801920   10.98069081412547
O   9.66604749908960   16.04313494053051   11.00513724225945
O   12.49511664149285   2.96362148825317   11.13219282181385
O   9.34064129172303   3.14511073750177   11.07854461864242
O   6.80130340265002   5.40681850274440   10.98940020544316
O   6.91237515720047   13.87443282912983   10.89063362228985
O   15.16912824550280   13.47476469532535   10.93104666409762
O   4.38573457375100   10.83608550124059   10.97854098162263
O   17.61525518094441   7.94602192958691   10.93186528009607
O   15.01671619144447   5.36511478565075   10.91274982416293
O   17.62595283919699   10.76683554182069   10.92170737967450
O   7.16680093664123   10.95878384648246   10.91860777628669
O   4.43322954733451   8.06352312753602   10.93427790186315
O   9.96956484968756   8.76372156980567   10.25474151579811
O   12.42955319984117   8.06477366890476   10.91875452485927
O   12.23244256169253   10.83443025213651   10.82562833594089
O   14.95456029721106   10.76043952553551   10.84843791028483
O   9.69281443027429   13.24596039168009   10.89083893166643
O   14.97856448341811   8.08607133607067   10.83850527055571
O   12.46893587112439   13.45430004705852   10.83420540982918

O 9.56259360577861 5.76338851664328 10.94644715519044
O 12.24716078771129 5.47108494509464 10.84864807187643
O 7.14190936163571 8.14811567579467 10.90593381135500
O 9.63509657865999 10.83492869578584 8.26958303083309
O 7.14784910113831 8.17447871123820 8.11267457600287
O 12.45079532436723 13.54759682528995 8.12957766582537
O 9.74630955965233 13.49123804230099 8.11960463074876
O 9.55827137848493 5.84710275956510 8.17019875608936
O 15.04175266926185 10.77663549675377 8.16119200496390
O 12.28047892090570 5.52742257777393 8.20280237029103
O 12.40393079763890 10.88865957326583 8.12308865239122
O 14.89481092632461 8.09615322058417 8.16629939354378
O 12.18744498539496 8.17902367604309 8.17081946591668
O 6.92904388927040 10.92651959708147 8.13196219609648
O 17.68473361634153 10.81192683837886 8.10846155923148
O 15.25635361135069 13.60529437149935 8.22186479109253
O 4.42815091599832 8.07102232575386 8.12799531132878
O 7.04421472748892 13.70138443848905 8.07084673632572
O 17.56833458782326 7.96378564956755 8.09715672314241
O 4.23908833771110 10.85331914122915 8.03322554182867
O 15.04540463647665 5.37603514322899 8.13423146189339
O 6.70920558222605 5.36552942931811 8.20991749779275
O 9.66488307768375 16.15831858945280 8.09800604330076
O 12.55160098344338 16.17577488792701 8.10208777513677
O 12.46516871354616 2.99825123899356 7.91804235196365
O 9.28801921829236 3.22146494413011 7.89254579813032
O 9.67331298423334 10.91983429317179 5.56625502345180
O 12.31604899202724 10.84269989579460 5.45506659750263
O 12.30434133060247 8.19699106912377 5.54261602866628
O 9.56324488902805 8.20296034838932 5.86413423896288
O 6.66840851095044 8.24436829304610 5.32317961321395
O 15.18785328642866 10.70243931696658 5.34840453970084
O 12.31274586569591 13.70232159533386 5.34534595708953
O 15.07313476870926 7.97202956900667 5.46884906258551
O 9.56794418120320 13.67075630127603 5.44305734961094
O 9.66080459762757 5.32663026467224 5.30231464111510
O 12.39184016710122 5.44514996577303 5.46020304345109
O 6.91069963811315 10.97864106892630 5.45112723361920
O 9.40489365186709 11.10283576241116 3.03440524237367
O 12.59161483927166 11.11162105335581 2.95896033088344
O 9.31067143582647 7.91431038307878 3.22933800823889
O 12.50675372932338 7.90027574016679 3.01538776569287
Pt 11.10812410010353 16.21975796839767 9.54245539225906

$Pt^{2+}/Sn^{4+}(Ce^{3+})_2(Ce^{4+})_{37}O_{80}$ ***E = -952.95205***

Ce^{3+} 18.68504079137204   9.34844531312048   9.50131021907403
Ce^{3+} 3.30053822222516   9.58617338641890   9.51615117555172
Ce   13.53992720427328   9.41759550116254   14.94065264307496
Ce   8.44397578713314   9.49035265551239   14.92675499511339
Ce   10.96629326937694   6.85114207394451   14.86461959696419
Ce   11.02296674843074   12.05918831996207   14.94543516790797
Ce   8.34340318683845   12.23086016173502   12.25484190538645
Ce   8.25678637315114   6.73800505881538   12.27672585935311
Ce   13.63579532847750   6.67462201288902   12.25927904090627
Ce   13.73532781685809   12.16746720137072   12.30098209049745
Ce   16.20003528491361   9.37888954856603   12.20163992187648
Ce   5.81448518037928   9.51649546219984   12.17062936476845
Ce   10.98307529540198   9.49275406629306   12.15420932267837
Ce   11.06281918662926   14.76392385092678   12.21364216061336
Ce   10.92898431545245   4.09810905988220   12.10660876588812
Ce   13.70359203935339   14.74855959403159   9.51859821591088
Ce   8.41807788631069   14.82888131199940   9.49894597772130
Ce   5.79704020768305   12.21034993449957   9.47839517328211
Ce   16.27144331189248   12.06518967351001   9.51270571371267
Ce   11.00559167182256   12.14193482994163   9.51881811075782
Ce   13.59138307129221   9.44651000079145   9.50509888116913
Ce   10.94235391121295   6.85327057084113   9.49671634012711
Ce   16.14570655383076   6.70171326912521   9.49106288026512
Ce   5.78083809624308   6.90181810938100   9.52336341768223
Ce   13.45810621543609   4.03069733041658   9.49077025544410
Ce   8.37568339696775   4.11689682735837   9.49863016444515
Ce   11.06446476907352   14.76781562009197   6.81654936557960
Ce   10.91192285385646   4.12360121955574   6.87679903122050
Ce   10.96418751733532   9.50287621163145   6.86932778268384
Ce   5.78558519035087   9.59343531078474   6.82565325815035
Ce   16.19256813031233   9.40291206309664   6.80340688990293
Ce   13.72834140454927   12.17940959344975   6.72811107753126
Ce   8.28779958631891   12.28349415352918   6.70256455098287
Ce   13.62551673574541   6.68538547651428   6.72582353206096
Ce   8.27996204716948   6.80832347339005   6.75923627413537
Ce   11.03258789434608   12.08939781607073   4.08433748797461
Ce   10.93123973531036   6.89017202755707   4.12468706914779
Ce   8.42429107397852   9.53544195815181   4.08573578027561
Ce   13.53252508737307   9.44193730888593   4.07841494498333
O   12.56912801523771   7.84959323036090   15.99925127088412
O   12.57709297134256   11.03765893310201   16.02611565174593
O   9.39713060255202   7.92213088814983   15.98618243937925
O   9.41859089805591   11.09537548915367   16.01742648108870
O   6.93038400105627   10.94912644208477   13.46467347121550
O   9.65964036440661   13.60165012865125   13.55106524659503

O	12.43796389843914	13.57055210048716	13.57581864501782
O	15.09732238107015	10.81192172082495	13.55495624481319
O	15.07491962311335	8.03684132362332	13.55233522813232
O	9.55556757049399	5.43130198552756	13.58435699894086
O	12.34473650716520	5.40525755398069	13.56355742295260
O	6.87942759421804	8.16065806863345	13.58106500903957
O	9.64856686889278	8.15902778177277	13.45254976744856
O	12.30244348415978	8.14334405414261	13.48257429087529
O	12.34102121347900	10.80881356106276	13.49788556355132
O	9.68778191082435	10.83588452296654	13.49992035607539
O	12.52353704028540	16.12736031504440	10.96177479380330
O	9.62849805331816	16.16274513198508	10.95701596379672
O	12.49232235364279	2.96725960444494	11.09148897462074
O	9.32823154664380	3.02881914835759	11.07744483624978
O	6.84575439318904	5.48270614170384	10.88520451860643
O	6.97556369830156	13.56050985241519	10.88091763837822
O	15.12881357049898	13.50395060642431	10.88696152040116
O	4.44275776492081	10.99043369851296	10.90675808743853
O	17.58840722238513	7.96891811338900	10.91106207914972
O	15.02112476029643	5.37908250741949	10.87984283723181
O	17.64382274053841	10.80196108886076	10.90598361202013
O	7.11614690200334	10.75988602033855	10.67444353643661
O	4.35744735624112	8.16597342324339	10.92732576926318
O	9.55284967535244	8.25117695555702	10.78583948984154
O	12.30992010835085	8.12643107066831	10.86233960433320
O	12.36432639917366	10.84798577929547	10.86173307507730
O	14.99478967149902	10.77507094907711	10.85314647389235
O	9.69687550320012	13.51216913757731	10.85603309585872
O	14.93929230929853	8.08908796212417	10.83925276947718
O	12.39857470245026	13.46669307390120	10.87693669244053
O	9.61276924097908	5.56601430396698	10.85098562232033
O	12.25970943265911	5.49488268116322	10.82384338215141
O	9.64014311278910	10.87543983787180	10.86044130022171
O	7.03964184469564	8.18000233455215	10.87397524580878
O	9.52456769666659	10.80676030346300	8.25361250705078
O	7.07021798586438	8.40145059386365	8.29518214237070
O	12.39879853206236	13.47213164957492	8.16057378972746
O	9.69130169949202	13.48134154071472	8.13633420662014
O	9.61568170127119	5.54782075276996	8.17300033749751
O	14.97489317300602	10.78489728235936	8.16527616416407
O	12.26288141493819	5.51151520077718	8.15846442975823
O	12.32129439356567	10.85789822452326	8.15639197618043
O	14.93790986698141	8.09562269958752	8.15318029347322
O	9.58522493260432	8.18668660411885	8.17025007373297
O	12.31522204511438	8.14020842842244	8.14831153119635
O	6.98588205053721	11.00424281012825	8.06908133514595
O	17.62279535821717	10.81046784112745	8.10623325747704

O   15.12701246196789   13.51061515371055   8.14844130626022
O   4.39073154242432   8.19517084770121   8.09698893485220
O   6.96583259569898   13.70442687388551   8.13855424233327
O   17.59197607582412   7.98381878665392   8.07172935736934
O   4.34236156145496   11.02121294503142   8.11081584756038
O   15.02290710041180   5.38314799873890   8.10341719478876
O   6.85155859364952   5.60458134817181   8.09700513859689
O   9.63490198690356   16.16068870409199   8.06681827848481
O   12.53121293700420   16.12881646375396   8.07708064681308
O   12.46258674176997   2.98190117344603   7.89650573705824
O   9.29357423215134   3.03893161558252   7.89355903968008
O   9.67937247838489   10.89667885978739   5.55939116796115
O   12.33427002322943   10.81970064441241   5.52523750520137
O   12.28471372498139   8.15740799436655   5.52859917688893
O   9.63999067858215   8.22199434605317   5.52601043005693
O   6.87037405651636   8.17765840457599   5.52054751135182
O   15.09366134301467   10.84479911221314   5.46361853333478
O   12.44254290931327   13.58380752044436   5.45809955453833
O   15.05841161654154   8.06916891383273   5.43655806655693
O   9.65777027806600   13.65453581968397   5.44003023679141
O   9.54356070111219   5.49261104050307   5.43570165754165
O   12.32546388637553   5.42206673301618   5.42062321592185
O   6.92119553561739   10.95715012543161   5.39721593920669
O   9.44337144261101   11.11836589299694   3.01220762350196
O   12.59302945009077   11.08096307354327   3.00049118012236
O   9.33614303254808   7.93909665163564   3.01528949945771
O   12.52888225393690   7.89660978769958   3.00269779609900
Pt   11.08086735856969   16.25405172731798   9.51617544546704
Sn   8.42605339432450   9.50671263046344   9.52347828043380

$Pt^{2+}/Sn^{4+}(Ce^{3+})_4(Ce^{4+})_{35}O_{79}$ E = -946.97700

Ce^{3+} 18.67666310457606   9.33293373726624   9.50657244096664
Ce^{3+} 3.33199380059393   9.51431607316933   9.51562594295001
Ce   13.49897234469569   9.40438451453064   14.90948494399866
Ce   8.38896375216299   9.53336950203237   14.85364270213049
Ce   10.95506190354902   6.84581332710566   14.82142214860250
Ce   11.00074420258014   12.04582026516265   14.91658916968671
Ce   8.30165160162435   12.31225674423754   12.27756171172799
Ce   8.08603664248055   6.58809782021589   12.47625539275961
Ce   13.63157894864328   6.67325610541398   12.25763552815940
Ce   13.72697109190091   12.15242500065269   12.30228730077584
Ce   16.18882302478366   9.37018447306785   12.19692405440745
Ce   5.78157833020444   9.53625322467769   12.20168159883032
Ce   10.91908908409189   9.49006393568989   12.05998647577099
Ce   11.09750819557005   14.77035980853342   12.21183799383080
Ce   10.90350590846600   4.10097183883280   12.12501882363342

Ce 13.73518786189471 14.73939738111112 9.51683956429452
Ce 8.43323990501087 14.85493808328687 9.52661309463637
Ce^{3+} 5.65726236908659 12.33354568717865 9.52895360736120
Ce 16.27246824189076 12.05484605850626 9.51841349597955
Ce 11.00501222049351 12.14613672998116 9.51675139791031
Ce 13.57683756707529 9.43606220756941 9.49862936660750
Ce 10.98259603514675 6.78744667793468 9.51973526521732
Ce 16.14178599797386 6.68928721457360 9.49554100775976
Ce 5.92680847442431 6.94306416942390 9.53701294475141
Ce 13.44552692482668 3.98376190106576 9.49994417470538
Ce 8.34686325708061 4.21953473352380 9.49918781771628
Ce 11.09258897224152 14.77979560218990 6.82223136147251
Ce 10.91375488418325 4.15558918392319 6.86543942460908
Ce 11.00118185258036 9.54530833825862 6.84521061659227
Ce 5.82476069167402 9.59013176475612 6.86373014978952
Ce 16.19204958338136 9.40626030791584 6.80262594394713
Ce 13.74731222133501 12.19234523199334 6.71999938806611
Ce 8.30367471780173 12.31897186975386 6.76836524267907
Ce 13.65731873041598 6.68818697852287 6.73282683110501
Ce^{3+} 8.02040319586174 6.53453493802455 6.46960661874571
Ce 11.01869432787135 12.09281645264058 4.08004132720484
Ce 10.97066044784408 6.86754288825471 4.18943332429801
Ce 8.40699792699598 9.55364077837298 4.18764820875150
Ce 13.53924159152312 9.44448791982436 4.05595887207554
O 12.56840091410628 7.80556017797536 15.96691460423029
O 12.54823869879974 11.01359786143250 16.00172749422277
O 9.39523508040109 7.94235059257927 15.85124478938758
O 9.37084995688295 11.10839012222622 15.97814044444152
O 6.92232797785468 10.93543464291313 13.54679234676475
O 9.68441913849892 13.60925143868095 13.57791254828209
O 12.46240234370642 13.55084665966020 13.57295869786768
O 15.08272444949752 10.79102528818415 13.55446161697220
O 15.02709544174910 8.01983737500361 13.54007019761031
O 9.50358857108046 5.34816859828699 13.67756633728114
O 12.31028748865068 5.40229109603340 13.55444296241729
O 6.78217469961559 8.12158604132191 13.65479616050450
O 9.54922600948826 8.11143115871924 13.21682314432917
O 12.22500534987486 8.15266257393892 13.45728663914808
O 12.30503450837194 10.78792830617566 13.44989780833864
O 9.67578600740104 10.81655381154495 13.46390247186368
O 12.58238233854575 16.11399222250352 10.96887752090094
O 9.69883516390755 16.19232399022368 10.97125941473906
O 12.46974365104811 2.94592047456065 11.10936759051818
O 9.31828045068833 3.09932593930890 11.06587187329682
O 6.81567751466392 5.47105131882182 10.99286278478872
O 7.04924975461595 13.72126989497938 10.96192961258914
O 15.15173976153922 13.48356080081448 10.88906348599992

O	4.28565484071336	10.87479021181275	11.00178840286845
O	17.58797322034102	7.95235269439365	10.92407613624088
O	14.99947780961332	5.37486785035576	10.88607625881529
O	17.62260308408844	10.78026768751462	10.91993620666016
O	6.99665333722333	10.87835709866548	10.81889083656221
O	4.49885797693474	8.10368644319746	10.92525506924496
O	9.83835818888357	8.56280914084891	10.03433673991959
O	12.34744075084406	8.08077496117467	10.88570067269140
O	12.32063216996309	10.82194874340087	10.83665785413765
O	14.96756651552301	10.75944885384570	10.85008605278058
O	9.73855837439901	13.53949867996024	10.87634800256650
O	14.94525598708356	8.08496532613955	10.82953827862783
O	12.42114424992491	13.44809907596838	10.87037616523241
O	9.55733452914641	5.69964795466375	10.93012342044701
O	12.24125225344478	5.45250178801458	10.83240047985152
O	9.53491470745479	10.96578661022705	10.86906021183006
O	7.24616087543227	8.25400259418809	10.93105061714224
O	9.57120677611116	10.73230699318018	8.20713815223719
O	7.35550637349570	8.31279235891787	8.20835790118578
O	12.42151423327499	13.46723439847840	8.16108710479142
O	9.73058873472100	13.47358643525430	8.16378552472992
O	9.58126857076874	5.74830327724400	8.12851784840919
O	14.98139200416464	10.77453824279609	8.16622575892872
O	12.29014410243056	5.48655669577560	8.16723331026957
O	12.35153842170674	10.86417760388778	8.14663516578417
O	14.90638060556505	8.09167451059241	8.15424354349661
O	12.22882861864208	8.13440365858882	8.14018060254153
O	7.02579190980651	10.95888777384106	8.22645191031060
O	17.63525646114753	10.80580148618739	8.11379436725742
O	15.15525396261829	13.50078083272714	8.14341294384347
O	4.56332465981932	8.12720824551836	8.12361655707669
O	7.05214345972436	13.76527897551970	8.10700743236970
O	17.57178021164755	7.97750239244788	8.08034916464539
O	4.29727329490590	10.89438748155264	8.05127027034097
O	15.04726605539673	5.37785268343850	8.10381947655923
O	6.75441015198338	5.42198457798399	8.20443251872883
O	9.69361992677682	16.16971836160365	8.07265393699695
O	12.58162728295017	16.12155396061120	8.07114622919661
O	12.43615212547475	2.95970378959221	7.91527137912433
O	9.26360517006055	3.16377796182920	7.88541371860130
O	9.70216572111316	10.89751491580838	5.55963740105448
O	12.33980826550126	10.82243550319903	5.52063207389658
O	12.30434352029178	8.17862807472257	5.52906766690275
O	9.60589254897235	8.16359180979203	5.79839654603599
O	6.74253159363385	8.27712999705451	5.35915337786878
O	15.10599567947462	10.84556654073457	5.45847847661404
O	12.47003233478940	13.58428822361046	5.45400835861479

O 15.08015146360982 8.06729108330602 5.44186733187689
O 9.68642015550492 13.65802262580184 5.46392286261170
O 9.66080671604958 5.31092783068674 5.27796512786756
O 12.39928430029543 5.43595981771140 5.44166155085109
O 6.95312825938869 11.03042172935207 5.50375224053333
O 9.41934941352189 11.11239061733887 3.03758784328930
O 12.58052203313821 11.07383917664902 2.99277943418544
O 9.34934712352817 7.93233424983715 3.18466706985468
O 12.55917041039714 7.87848106607724 3.01246367516446
Pt 11.13746913002013 16.25775150757794 9.52058887633860
Sn 8.24686973772952 9.72590035961698 9.54843796866247

$Pt^{2+}/Sn^{4+}(Ce^{3+})_6(Ce^{4+})_3\ O_{78}$ E = -940.68907

Ce^{3+} 18.70517153303391 9.23282322910580 9.51008208417951
Ce^{3+} 3.35940334278895 9.50765307253916 9.50944736430321
Ce 13.54578564446528 9.41849427616671 14.91867721279070
Ce 8.40742861122906 9.51780234899696 14.78095166142403
Ce 10.97161575476908 6.84895272919056 14.86823116015114
Ce 11.05835272290322 12.07327956827368 14.82881778547574
Ce^{3+} 8.06439406765389 12.56414544976674 12.54198589342151
Ce 8.09156385520289 6.54086334297115 12.48786104041957
Ce 13.65543207650363 6.66556814525252 12.27262209040564
Ce 13.78918313488976 12.17556958505144 12.26571624315805
Ce 16.22005616600176 9.36355742785806 12.19024244535838
Ce 5.86103262603971 9.51050656482997 12.17186273183029
Ce 10.99926944493044 9.44444566255166 12.13991695567830
Ce 11.10197320280954 14.72598685271186 12.19034277340883
Ce 10.92354618748408 4.12169254655397 12.15054228945639
Ce 13.75932046007771 14.75105135927604 9.50783116111841
Ce 8.41386082807231 14.79023669418800 9.52289819717396
Ce^{3+} 5.72627427392180 12.31395300102045 9.50814573250261
Ce^{3+} 16.37225574948734 12.09199274793308 9.50314413731746
Ce 11.06322787112466 12.20468897455808 9.45099014387539
Ce 13.60756392995442 9.47304761043383 9.50379268065163
Ce 10.95874524188884 6.85414807888506 9.54923923042366
Ce 16.14887886131320 6.67625548798275 9.51067271271465
Ce 5.86943338377646 6.87588224904176 9.52952171109622
Ce 13.43706075895848 4.02631186750567 9.51652737611083
Ce 8.34811562237085 4.23287465315912 9.49758156873916
Ce 11.08371027173347 14.80139007744840 6.80110881619320
Ce 10.93074186046427 4.20038982217127 6.87398662236199
Ce 11.00421638225500 9.56880768034298 6.85235078515581
Ce 5.76828963347009 9.57563803370566 6.79852193179442
Ce 16.22428287522541 9.39477509002750 6.82208891156258
Ce 13.79363988429785 12.21916069158308 6.71597615737612

Ce 8.28925114523735 12.31914599886336 6.73627630704514
Ce 13.65957242640502 6.71126980564245 6.75946280233410
Ce^{3+} 8.00367895123742 6.52655672691215 6.45332250706911
Ce 11.03586042235957 12.11437109344911 4.09597654334008
Ce 10.97818607890264 6.88639765104223 4.20511831854597
Ce 8.40713793199155 9.55540944453175 4.18329196258860
Ce 13.55341069620991 9.46215519901987 4.08886881239169
O 12.57074123903569 7.82271426447568 15.98833017393844
O 12.55974302554232 11.02763505350003 15.98152858421873
O 9.39891995702569 7.96431375379551 15.86347991364401
O 9.37210172067683 11.13402727707361 15.80815939523589
O 6.77453122195142 10.84085140020089 13.65637957230365
O 9.78265827979888 13.75287383140139 13.69534500422041
O 12.51495600921982 13.52231227501002 13.54705420453510
O 15.11736732593121 10.78710223580667 13.56653885130459
O 15.02613638689839 8.00801039901301 13.55965266021161
O 9.50608968359675 5.33099559096578 13.69534291140948
O 12.31460983255320 5.40112070703631 13.57651671048503
O 6.81594355100463 8.05510728009853 13.60729268691368
O 9.57185650549730 8.08480797298225 13.24616885270959
O 12.25058416345423 8.13743177248521 13.48759942515034
O 12.34925790149601 10.76924920889464 13.44620050245265
O 9.64485465807121 10.86309922327462 13.20453116363151
O 12.54647787917411 16.10614024198237 10.96345734363632
O 9.65339223340888 16.06729158366431 10.98696081502493
O 12.46781767586065 2.97397407545016 11.13977933442426
O 9.31866987361769 3.14696049674256 11.07298181618798
O 6.79968938080474 5.43461412204405 10.98743942712699
O 6.90911275314623 13.87975181186939 10.87380356222215
O 15.08883007573616 13.57147938959096 10.93490794806292
O 4.46637420353058 10.85079385379697 10.95760868984807
O 17.57636475773058 7.84211087097862 10.92366996317528
O 14.96777094782866 5.34790916927666 10.90921331104809
O 17.69805036831472 10.62579806286483 10.97665569352753
O 7.35085912217000 10.91669092039213 10.84961356483462
O 4.48801865388420 8.07780281336172 10.92393864224739
O 9.86002107900858 8.82038896435665 10.19788784091689
O 12.36103230067904 8.08504165884776 10.89958137172452
O 12.22332862251863 10.82997677175971 10.81976906043088
O 14.96076423663329 10.76178897491363 10.86593121705195
O 9.67568738532756 13.28478795430415 10.89249428232341
O 14.94383132095225 8.08070524502316 10.83911769083996
O 12.41390014539775 13.44262356722303 10.83371017841148
O 9.54485057456762 5.76573088255097 10.93547045611865
O 12.20748658468692 5.48863196311270 10.83927238755569
O 7.22366316951828 8.20673347112148 10.85389888745578
O 9.55715111768389 10.76074490410159 8.34553112812659

O 7.33879931362565 8.23880533881510 8.15111037695987
O 12.40212732405967 13.48227146031589 8.13904368224018
O 9.72945912189893 13.46841112351335 8.12582142036724
O 9.56001313792137 5.81991994623571 8.15627762560284
O 15.00318581901054 10.77755750149930 8.13974590494181
O 12.25010304350427 5.54086537120626 8.20345196754658
O 12.37401505312114 10.88841705760408 8.13442370507283
O 14.89253288620204 8.09464260272219 8.17184848298818
O 12.19028466388911 8.17676433717457 8.17873272767100
O 6.98147137018422 10.87812844302776 8.18535312456696
O 17.72730054661916 10.63938096266788 8.03120040484490
O 15.08723967093146 13.59039718539737 8.07235352548113
O 4.53688468531935 8.11219131293781 8.11741121932227
O 7.04468090243901 13.69387932950989 8.03869586286161
O 17.54920964481029 7.86398992460924 8.10459293118076
O 4.27015406740566 10.88079827776605 8.01757139533017
O 14.99719932602993 5.36399004455379 8.12204027063403
O 6.72355564113467 5.39592827270942 8.19084841285980
O 9.64979408758043 16.14520059293362 8.07701122855105
O 12.52456177476911 16.14592742708692 8.07089188476864
O 12.43659296523349 3.00394852552458 7.91708099512491
O 9.26257309988591 3.20734167186057 7.87825853362746
O 9.68855822723894 10.90756126788658 5.60488049702964
O 12.33127617508467 10.83806270171480 5.53109865284503
O 12.29451385182802 8.20009446900593 5.56227224332529
O 9.59162527847509 8.18531959842581 5.81704563192041
O 6.70683890783380 8.26196730889915 5.33138003213358
O 15.10381128101881 10.83936437954056 5.43777551168954
O 12.45419818584124 13.60450192304285 5.43576433750560
O 15.06183543779152 8.05786980500266 5.45563994313718
O 9.66792485851249 13.65875018044672 5.43879483101041
O 9.66743494877493 5.32845109432885 5.28666068773180
O 12.40197705391690 5.45356698680684 5.46504154675189
O 6.92925578894967 11.01575241494257 5.47100586478508
O 9.42544563503068 11.10632861347880 3.06354878420054
O 12.58895895438478 11.09208510230885 3.01068508947364
O 9.33672879883352 7.92512849715568 3.19513640060628
O 12.55326468782768 7.89007413415389 3.04149270563144
Pt 11.09352888177043 16.21762118642236 9.52491682629291
Sn 8.16082595517236 9.62437555653153 9.45347436019360
